# Are Alternative Strategies Required to Accelerate the Global Elimination of Lymphatic Filariasis? Insights From Mathematical Models

**DOI:** 10.1093/cid/ciy003

**Published:** 2018-06-01

**Authors:** Wilma A Stolk, Joaquin M Prada, Morgan E Smith, Periklis Kontoroupis, Anneke S de Vos, Panayiota Touloupou, Michael A Irvine, Paul Brown, Swaminathan Subramanian, Marielle Kloek, E Michael, T Deirdre Hollingsworth, Sake J de Vlas

**Affiliations:** 1 Department of Public Health, Erasmus MC, University Medical Centre Rotterdam, The Netherlands; 2 Mathematics Institute, University of Warwick, Coventry, United Kingdom; 3 Department of Biological Sciences, University of Notre Dame, South Bend, Indiana; 4 Department of Statistics, University of Warwick, Coventry, United Kingdom; 5 University of British Columbia and British Columbia Centre for Disease Control, Vancouver, Canada; 6 Vector Control Research Centre, Indian Council of Medical Research, Indira Nagar, Puducherry

**Keywords:** lymphatic filariasis, elimination, mass drug administration, mathematical modeling

## Abstract

**Background:**

With the 2020 target year for elimination of lymphatic filariasis (LF) approaching, there is an urgent need to assess how long mass drug administration (MDA) programs with annual ivermectin + albendazole (IA) or diethylcarbamazine + albendazole (DA) would still have to be continued, and how elimination can be accelerated. We addressed this using mathematical modeling.

**Methods:**

We used 3 structurally different mathematical models for LF transmission (EPIFIL, LYMFASIM, TRANSFIL) to simulate trends in microfilariae (mf) prevalence for a range of endemic settings, both for the current annual MDA strategy and alternative strategies, assessing the required duration to bring mf prevalence below the critical threshold of 1%.

**Results:**

Three annual MDA rounds with IA or DA and good coverage (≥65%) are sufficient to reach the threshold in settings that are currently at mf prevalence <4%, but the required duration increases with increasing mf prevalence. Switching to biannual MDA or employing triple-drug therapy (ivermectin, diethylcarbamazine, and albendazole [IDA]) could reduce program duration by about one-third. Optimization of coverage reduces the time to elimination and is particularly important for settings with a history of poorly implemented MDA (low coverage, high systematic noncompliance).

**Conclusions:**

Modeling suggests that, in several settings, current annual MDA strategies will be insufficient to achieve the 2020 LF elimination targets, and programs could consider policy adjustment to accelerate, guided by recent monitoring and evaluation data. Biannual treatment and IDA hold promise in reducing program duration, provided that coverage is good, but their efficacy remains to be confirmed by more extensive field studies.

Lymphatic filariasis (LF) is a mosquito-borne parasitic disease endemic in 72 countries [1], affecting millions of people, with many of them suffering from hydrocele or lymphedema (elephantiasis), associated mental health problems, social marginalization, or reduced productivity [2–4]. The Global Programme to Eliminate Lymphatic Filariasis (GPELF) was set up in 2000, aiming to eliminate LF worldwide as a public health problem by 2020. Two pillars of the program are to (1) interrupt transmission by implementing at least 5 rounds of annual mass drug administration (MDA) of a 2-drug combination (ivermectin + albendazole [IA] in onchocerciasis-endemic areas, and diethylcarbamazine + albendazole [DA] elsewhere), and (2) reduce the suffering of patients through proper morbidity management [1]. In this article, “elimination” refers to the achievement of this first objective via the reduction of infection to below a 1% microfilariae (mf) prevalence threshold.

Great progress has been made toward the 2020 elimination goals. More than 820 million people have been treated at least once [1]. According to the Global Burden of Disease study, the number of infected cases declined from 53 million in 2000 to 29 million in 2016. In the same period, the burden of disease in disability-adjusted life-years declined from 1.9 million to 1.2 million [5]. Eighteen endemic countries have completed their MDA programs and are under post-MDA surveillance, and 25 countries have successfully implemented MDA in all endemic districts and may be considered on track to elimination. In some treated areas, however, the decline in mf prevalence has been slower than expected, possibly due to low coverage, mosquito vector characteristics, high transmission intensity, or differences in efficacy between treatment regimens [6]. Furthermore, another 20 countries are treating only part of their endemic districts, and 9 countries have not yet started [1]. The untreated areas include *Loa loa–*coendemic areas in central Africa, where DA and IA are contraindicated because of safety concerns. The provisional strategy for these areas is to implement twice-yearly MDA with albendazole alone, in combination with vector control [7, 8].

Program acceleration is required to maximize the number of countries achieving the 2020 elimination targets and minimize the additional efforts required after this year. Keeping the programs short is also important to sustain the global momentum for LF elimination among all stakeholders. Therefore, the World Health Organization recently published new guidelines on alternative MDA regimens to eliminate LF [9]. Alternative strategies considered in this report include increasing the frequency of treatment from annual to biannual (every 6 months), using the supposedly more efficacious triple-drug treatment combination of ivermectin, diethylcarbamazine, and albendazole (IDA) [10], and biannual MDA with albendazole alone for loiasis-coendemic areas, where neither diethylcarbamazine nor ivermectin can be used in view of high risk of side reactions. Unfortunately, empirical evidence on the comparative effectiveness of current vs alternative proposed strategies is still limited [9].

Mathematical models provide powerful tools to compare the impact of alternative interventions for infectious diseases when empirical evidence is insufficient [11]. Three mathematical models for LF are actively being used to support policy making for LF elimination programs, named EPIFIL [12, 13], LYMFASIM [14–16], and TRANSFIL [17, 18]. While there are many similarities between the models, there are also important differences in model structure, parameter quantification, and application methods, which can result in different predictions. Recognizing the need for robust policy advice, the 3 respective modeling groups are now collaborating in multimodel comparison studies and providing combined model predictions. They showed that all 3 models can reproduce observed trends in mf prevalence during MDA or integrated vector management [19] and that estimates of the required duration of MDA to reduce mf prevalence below the 1% critical threshold are of the same order of magnitude, although differences remain [20].

In the current article, we use these 3 models to estimate how long MDA programs still have to be continued with current strategies, and to what extent the required MDA duration can be reduced by optimizing coverage, increasing the frequency of treatment to biannual administration, or using IDA. We do this by simulating scenarios for 3 geographic regions and a wide range of baseline endemicity levels, both for treatment-naive areas and for areas with a history of MDA where strategy adjustment is considered.

## METHODS

### Simulated Settings and Scenarios

We performed simulations for bancroftian filariasis endemic settings in India, Africa, and Papua New Guinea (PNG), accounting for regional differences in human demography, local vector species (*Culex quinquefasciatus* in India, *Anopheles* species in Africa, and PNG), the possible range of precontrol mf prevalence levels (1%–15% in India, 1%–40% in Africa, 1%–70% in PNG), and the regional standard treatment regimens (DA in India and PNG; DA, IA, or albendazole in Africa, depending on coendemicity of onchocerciasis and loiasis).

Settings were further defined by the local history of MDA until present (t = 0), considering 4 alternatives: treatment-naive (TN) settings, which have not received any previous treatment and t = 0 is the precontrol situation; recently started (RS) settings, which have had 2 rounds of MDA before t = 0 with 65% coverage per round, and t = –2 is the precontrol situation; failure type 1 (F1) settings that have had 10 annual rounds of MDA before t = 0, but with low coverage (50%); and failure type 2 (F2) settings that have had 10 annual rounds of MDA, but with very low coverage (30%). For F1 and F2, t = –10 is the precontrol situation. Historical MDA rounds always involved the regional standard treatment regimens as given above. Coverage is here defined as the proportion treated per round out of the whole population. Poor coverage in F1 and F2 settings is assumed to be associated with systematic noncompliance, meaning that some people never take treatment, although this is only captured by LYMFASIM and TRANSFIL (see Supplementary Data 1–3 for details).

For each combination of region and history of control, we simulated trends in mf prevalence under alternative future treatment scenarios. Reference scenario is the currently recommended strategy of annual MDA with the regional standard regimen (DA, IA) with 65% coverage per round. Alternative scenarios include an increase in coverage (from 65% to 80%), an increase in frequency (from annual to biannual), or a switch to IDA treatment. For *Loa loa*–coendemic areas, we considered twice-yearly albendazole with 65% or 80% coverage. For F1 and F2 settings, we also examined what would happen if annual MDA were continued with the same low coverage.

### Models and Assumptions on Key Parameters

All 3 models simulate LF transmission in a closed, age-structured human population, tracking trends in various infection indicators over time. They capture the basic processes determining transmission, including characteristics of the parasite life cycle, age-specific rates of exposure to vectors, and vector biting rate and efficiency. EPIFIL and LYMFASIM also consider acquisition of immunity to infections. The models mimic the impact of MDA, accounting for treatment effects on different parasite stages, achieved coverage, and (for LYMFASIM and TRANSFIL) underlying compliance patterns (who is being treated in each round and who is not). They differ in the details of the implementation methods and parameter quantification. EPIFIL is a deterministic population-based model, while LYMFASIM and TRANSFIL are stochastic individual-based. LYMFASIM also models worms individually, while TRANSFIL models them at a population level inside the host.

Here, all 3 models used the same assumptions on treatment efficacy, with treatment regimens differing in the proportion of adult worms and mf killed, and a temporary or permanent reduction in mf productivity by surviving adult worms, as indicated in Table 1.

**Table 1.** Treatment Efficacy Assumptions Adopted

Treatment Regimen	Proportion of Adult Worms Killed, %	Duration of Sterilization, mo	Proportion of Microfilariae Killed, %
DEC + ALB	55%	6	95
IVER + DEC + ALB (optimistic)^a^	55%	Permanent	100
IVER + ALB	35%	9	99
ALB	35%	0	0

Abbreviations: ALB, albendazole; DEC, diethylcarbamazine; IVER, ivermectin; mo, month.

^a^Triple-drug regimen, for which we adopted optimistic treatment efficacy assumptions.

### Simulation Runs and Analysis

We generated 10000 simulations for each setting (defined by region, history of control) and future treatment scenario, varying a selection of parameters such that resulting prevalence levels at t = 0 capture the entire region-specific precontrol prevalence range for treatment-naive settings. LYMFASIM and TRANSFIL varied specific parameters concerning local transmission conditions (mean and variation in vector density or biting rate, external force of infection) with additional variation resulting from intrinsic stochasticity, while all other parameters were treated as fixed. EPIFIL varied 20 parameters, capturing not only variation in local transmission conditions and but also uncertainty in other factors (Supplementary Data 1–3). The corresponding prevalence levels at t = 0 are lower for RS, F1, and F2 settings, depending on the predicted impact of past interventions. LYMFASIM has not been calibrated for PNG and was not run for that region.

Our simulations start at t = –10 (ie, 10 years before the present) and end at t = 20 (ie, 20 years into the future). Future alternative treatment strategies start with their first treatment at t = 0 and are continued 20 years thereafter with treatment every 6 or 12 months. Historical treatments took place from t = –2 (RS) or t = –10 (F1, F2) onward, with the last historic treatment at t = –1. Per run, we stored the mf prevalence in the total population at yearly intervals, with the mf prevalence measured just before a treatment. Dynamic changes in between yearly measurements are not analyzed.

Per run we documented the year in which the mf prevalence first fell below 1%, assuming t = 21 for runs not achieving this target. Per setting and treatment scenario, we then calculated the moving average to show how this required treatment duration varied with the prevalence at t = 0 (window bin-size = 501). Prediction intervals were calculated by bootstrapping; 10 000 samples were taken out of the original simulation runs with replacement, for each sample the 90% interval of time to achieving the threshold was calculated, and finally the 95% range over all the ranges per sample was determined. We identified strategies that lead to elimination within 3 years, which would be sufficient to reach the 2020 target, if adjusted strategies are implemented from 2018 onward.

### Code Availability

The code for the version of LYMFASIM used in this paper is provided in Supplementary Data 4. The code of TRANSFIL and EPIFIL can be accessed via github [21, 22].

## RESULTS

**Figure 1.**
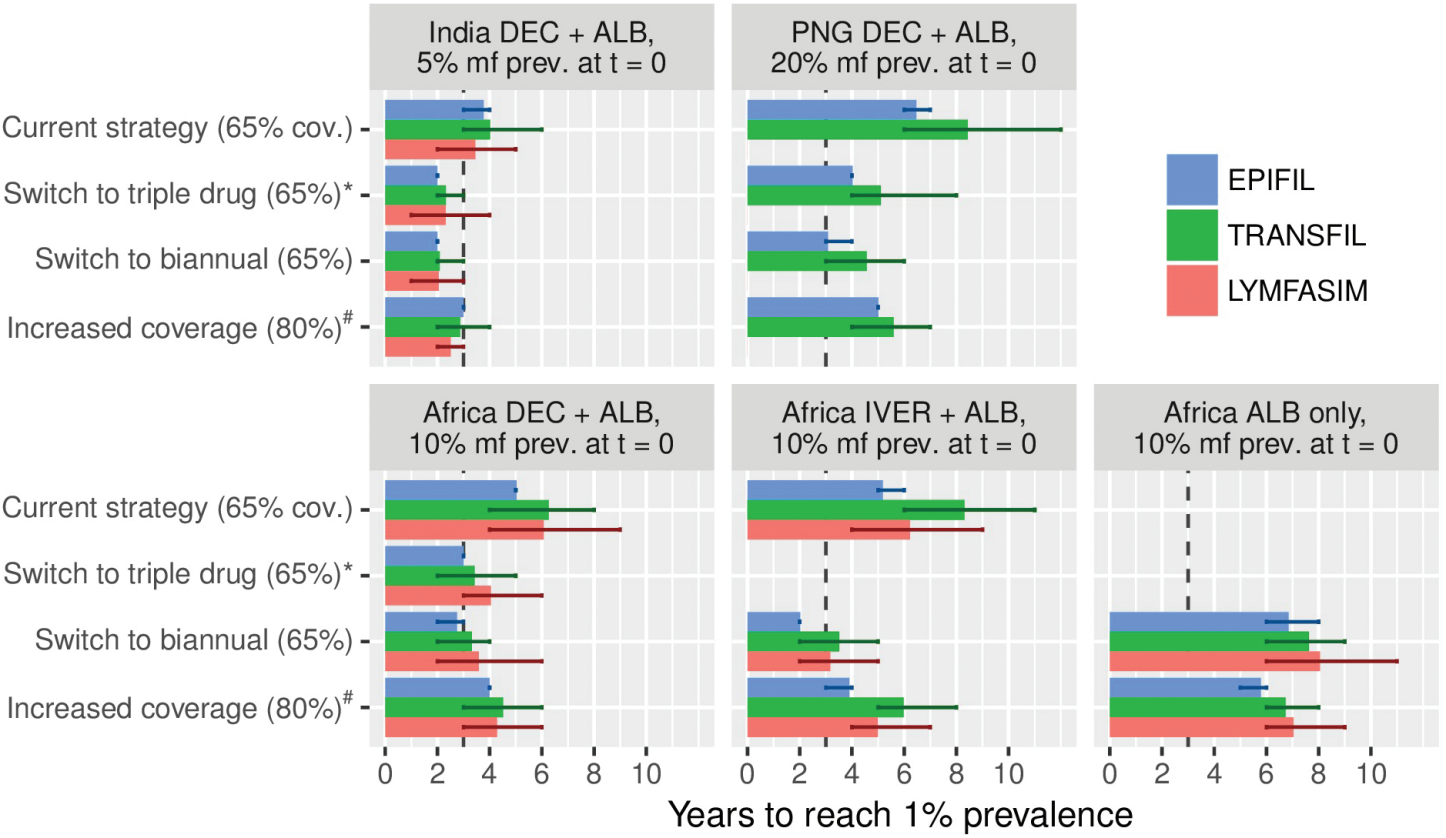
Required duration of mass drug administration (MDA) to bring microfilariae (mf) prevalence below the 1% critical threshold in treatment-naive settings, comparing the result from the 3 different models. The setting under consideration is specified in the caption of each subpanel, indicating the geographical region, recommended standard treatment regimen, and the precontrol mf prevalence considered. The current strategy (annual MDA with the recommended standard regimen at 65% coverage) is compared with alternative strategies differing in one aspect, including a switch to triple-drug regimen (ivermectin + diethylcarbamazine + albendazole; annual, 65% coverage), switch to biannual MDA (standard regimen, 65% coverage), or increased coverage of 80% (annual, standard regimen). Vertical line at t = 3 indicates the target year of elimination (ie, 2020, 3 years from the time of writing this paper). Error bars indicate the 90% prediction interval as calculated through bootstrapping. ^a^Simulations for triple-drug scenarios were done with optimistic efficacy assumptions, as provided in Table 1. ^b^Impact of increased coverage is shown for annual MDA, except for the “Africa ALB only” region, where it is shown for biannual MDA. Abbreviations: ALB, albendazole; cov., coverage; DEC, diethylcarbamazine; IVER, ivermectin; mf, microfilariae; PNG, Papua New Guinea; prev., prevalence.

Figure 1 shows the MDA duration required to bring mf prevalence below 1% for different strategies in treatment-naive settings, comparing the current strategy of annual MDA with standard 2-drug combination and 65% coverage with alternative strategies, that differ from the current strategy in one aspect—namely, the employed treatment regimen (switch to the triple-drug regimen), frequency of treatment (switch to biannual MDA), or the treatment coverage (increased to 80%). Corresponding trends in mf prevalence are presented in Supplementary Data 5, which also shows what happens if some of the above alternative strategies are combined (eg, triple drug or biannual MDA with 80% coverage). The required duration is shortest for the low-prevalence Indian setting, but still exceeds 3 years if MDA continues annually with DA at 65% coverage, meaning that elimination would not be achieved by 2020. Increasing the coverage to 80% results in some acceleration, but a stronger time reduction is obtained with annual IDA. Biannual treatment results in the strongest reduction, but requires more treatment rounds than the other acceleration scenarios. Similar patterns are seen in the other regions. While the 3 models yield very similar results for India, they are less consistent in the other regions (up to 3 years apart), with EPIFIL’s estimates usually being the shortest (Figure 1).

**Figure 2.**
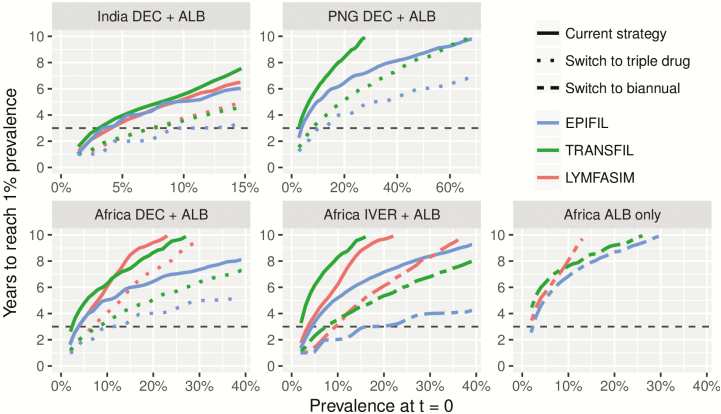
Average time needed to reach the 1% target of microfilariae (mf) prevalence in treatment-naive settings, in relation to the mf prevalence at t = 0. Each subplot represents a particular region as specified in its caption. Within each panel, results are shown for the current strategy of annual mass drug administration (MDA) with either ivermectin + albendazole (IA) or diethylcarbamazine + albendazole in comparison to ivermectin + diethylcarbamazine + albendazole (IDA; for India, Papua New Guinea, and the African DA regions) or biannual MDA (African IA region, where IDA is not recommended because of safety concerns). For *Loa loa*–coendemic areas, biannual MDA with albendazole alone is the recommended strategies and no alternative is shown. Horizontal line at t = 3 indicates 2020. Abbreviations: ALB, albendazole; DEC, diethylcarbamazine; IVER, ivermectin; PNG, Papua New Guinea.

The time needed to achieve the 1% threshold in treatment-naive settings depends strongly on the mf prevalence at t = 0 (Figure 2). For mf prevalence levels <3%–4%, 3 years of annual MDA with currently recommended 2-drug combinations is sufficient to reach the 1% threshold, but alternative strategies are required for higher prevalences. Switching from DA to IDA enables settings with current mf prevalence up to 10%–15% to achieve the goal in 3 years. In African areas with IA treatment, switching to biannual treatment means that areas with prevalence as high as 20% could achieve this time-bound goal, at least according to EPIFIL. The other 2 models predict that only areas with prevalence <10% would achieve the targets within 3 years.

**Figure 3.**
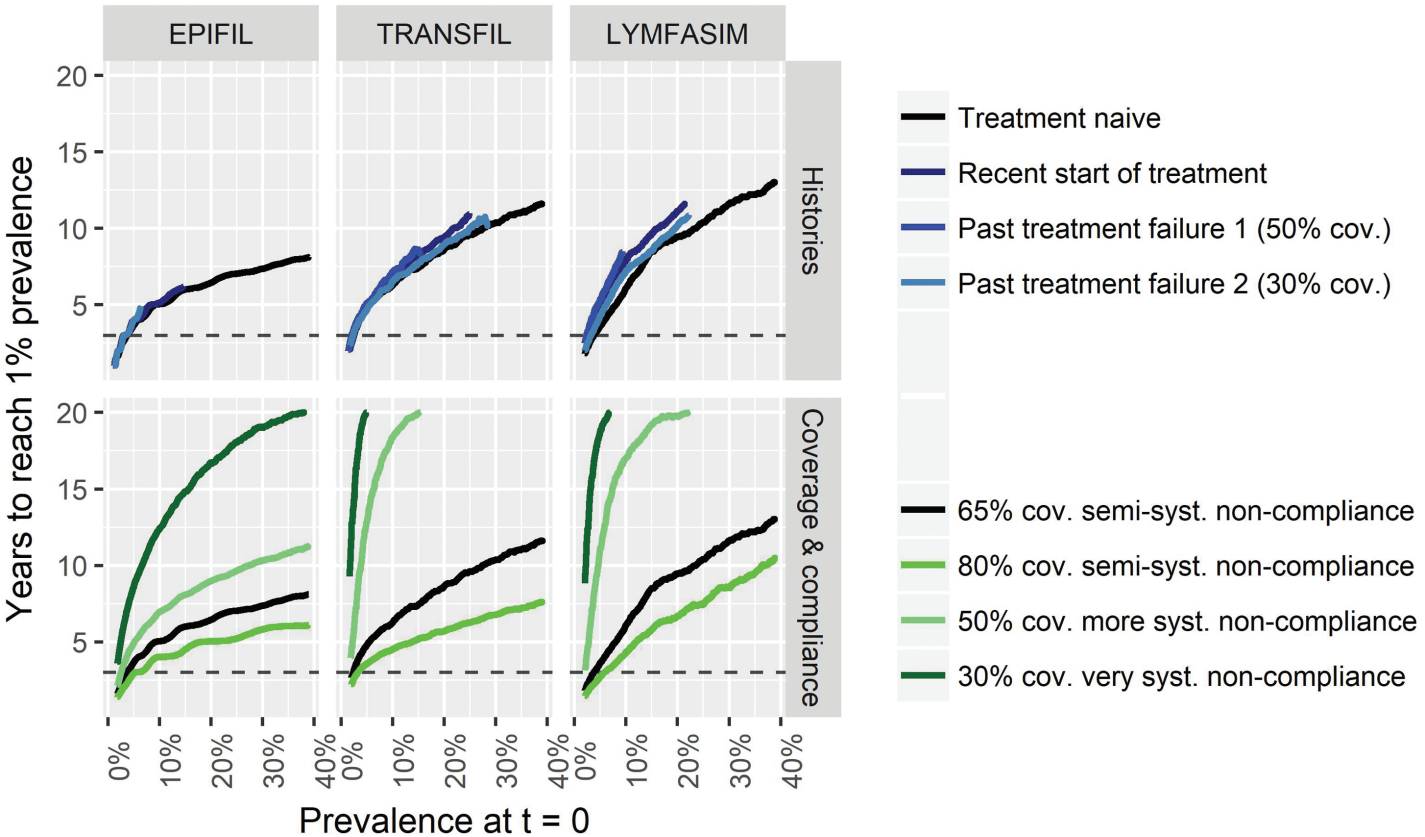
The association between the average time needed to reach the 1% target of microfilariae (mf) prevalence and mf prevalence at t = 0, for programs based on annual distribution of diethylcarbamazine + albendazole in the African region. The upper panels show how the history of control affects this association, assuming that coverage is 65% from t = 0 onward, independent of the history of control. The lower panels show how the program duration in treatment-naive settings depends on achieved coverage and compliance patterns from t = 0 onward. Horizontal line at t = 3 indicates 2020. Note that the black lines in the upper and lower panels per model are the same. Abbreviations: cov., coverage; syst., systematic.

Interestingly, the remaining required MDA duration depends only on current mf prevalence, treatment strategy, and achieved coverage, but not on the history of control, provided that problems with low coverage and systematic noncompliance are corrected. This is illustrated for annual MDA in African settings with different histories of control in Figure 3 (upper panels). Region also does not play a role for a given treatment regimen (Supplementary Data 5). The bottom panels of Figure 3 further illustrate the importance of achieved coverage/compliance for treatment-naive African settings treating annually with DA. The required duration can be reduced by 1–3 years (depending on baseline mf prevalence and model) if coverage is increased from an acceptable 65% to 80%, whereas a low coverage in combination with a high proportion of people never participating in MDA makes it virtually impossible to reach the threshold within a reasonable time frame.

## CONCLUSIONS

The importance of good coverage, with minimal systematic noncompliance, for the success of elimination campaigns is widely recognized [23–25]. Determinants of poor coverage are being studied, and methods for improvement are being developed [26–29]. While a coverage of 65% may be considered acceptable, increasing it to 80% leaves less room for systematic noncompliance and helps to shorten elimination programs. The benefits are even larger for settings with lower coverage or more systematic noncompliance than assumed here.

MDA durations can also be shortened by increasing the frequency of treatment, because a larger number of people will be treated at least once per year, and those treated twice are expected to experience a greater reduction in their worm and mf loads. Unfortunately, empirical evidence to corroborate the model-predicted benefit of biannual over annual MDA is scarce. Clinical trials with IA confirm that the overall reduction in mf count is larger in individuals treated twice, although there was no evidence of a larger adulticidal effect [30, 31], and one community intervention study reported a faster decline in mf prevalence during biannual MDA with diethylcarbamazine vs annual MDA [32]. Additional evidence is anticipated from ongoing community intervention trials. However, outcomes of such trials are notoriously difficult to interpret due to variation between treatment arms in baseline endemicity, between-survey variation in population samples, absence of individual-level longitudinal data, and uncertainty about achieved coverage patterns. The impact of increasing treatment frequency is highly dependent on the assumption that good coverage is maintained; the benefits would diminish if coverage drops due to implementation challenges.

Switching to the triple-drug regimen IDA is predicted to reduce the remaining duration of MDA programs by about a third. The superior efficacy of IDA over DA was shown in a small clinical trial from PNG [33] and confirmed in other, yet unpublished, trials [10]. The difference was attributed to a stronger adulticidal or more sustained embryostatic efficacy and the drug is now seen as a potential game changer for LF elimination [10, 33]. Accordingly, we assumed optimistically that IDA kills about the same proportion of adult worms and mf as DA, and also permanently sterilizes any surviving adults. Yet, the evidence base underlying these assumptions is still limited. If the sterilizing effect is lower or less sustained than assumed, the impact of annual IDA approximates that of DA, with no difference at all in the worst case [20]. More field studies are needed to better understand the efficacy of IDA.

When choosing between alternative treatment strategies, policy makers should also consider the costs, feasibility, and acceptability. While biannual MDA leads to the strongest predicted reduction in program duration, its implementation requires additional financial and human resources in already overburdened health systems [34, 35]. This may be less of a problem for strategies involving improved coverage or better drugs. Therefore, annual IDA treatment may be more attractive to many stakeholders, but its impact may decline with a lower population acceptation of having to swallow multiple tablets. Furthermore, diethylcarbamazine is contraindicated for MDA in onchocerciasis-coendemic areas, making IDA not an option for now. Yet, it could perhaps be used after a pretreatment with ivermectin, as suggested elsewhere [10].

Our study has some limitations. First, we have estimated the required program duration for bringing the mf prevalence below a 1% threshold, without assessing whether this eventually leads to true extinction of the parasite in the population. With a higher threshold of 2%, the required duration would decrease with about 1 year (EPIFIL) or 2 years (LYMFASIM and TRANSFIL), while similar increases in duration are expected if a 0.5% threshold is used. Previous modeling suggests that the threshold depends on local transmission conditions and declines with increasing transmission intensity [36–38]. The probability of true elimination after reaching a predefined threshold could be further investigated using the individual-based models, but even then it will be challenging to properly account for factors such as reintroduction of infection from neighboring areas. Also, some countries use antigen tests (eg, the filarial test strip) for surveys in sentinel and spot check sites, using an antigenemia prevalence <2% as target. The latter target is not necessarily equivalent to the 1% target mf prevalence, which could lead to different program durations than presented in this article. Second, our simulations are for single populations, whereas actual elimination programs typically take districts as their implementation unit. Within-district variation in baseline endemicity and achieved coverage and compliance patterns will lead to variation in the required MDA duration. Unfortunately, coverage data are often of poor quality [39]. Even if the mean reported coverage in a district is acceptable, there can still be sites with poor coverage, which is particularly problematic if it occurs in high-transmission foci. Third, we focused on improving MDA strategies and have not assessed the benefits of other strategies, such as supplemental vector control or diethylcarbamazine-fortified salt distribution. The latter in particular could be extremely effective, but it requires a very different implementation strategy than the current tablet-based programs [40, 41]. We showed previously that the impact of complementary vector control on program duration is limited [20].

All model predictions are subject to uncertainty. The error bars in Figure 1 capture uncertainty about the local transmission conditions underlying a certain prevalence and chance effects in small populations for LYMFASIM and TRANSFIL, and uncertainty in a larger set of parameters without chance effects for EPIFIL. By contrasting 3 models, we also capture variation resulting from structural model uncertainty. Usually EPIFIL is somewhat more optimistic than the other models, which is due to its assumption that treatments are randomly distributed over the population without systematic noncompliance. Between-model differences in assumptions on the parasite life cycle, density dependence in transmission processes, or population structure are probably less influential to treatment duration estimates, given the assumed high efficacy of available treatment regimens and corresponding limited bounce-back of LF infection between annual MDA rounds. However, they can give rise to important differences in estimated elimination thresholds, as shown elsewhere for onchocerciasis [42].

In conclusion, the remaining required duration of MDA for reducing mf prevalence below the critical 1% threshold greatly depends on the current mf prevalence and chosen strategy (treatment regimen, frequency, achieved coverage, and compliance pattern), but not geographic region and past MDA history, provided that any problems with poor coverage are solved. The ambitious 2020 target for LF elimination is unlikely to be met where mf prevalence is still well above 3%–4%. MDA programs can be accelerated by optimizing coverage, switching to biannual MDA, or switching to the supposedly more efficacious triple-drug regimen IDA. Yet even then the 2020 target likely remains out of reach for settings with prevalence >10%–15%, although the elimination threshold could be achieved a few years down the line.

## Supplementary Data

Supplementary materials are available at *Clinical Infectious Diseases* online. Consisting of data provided by the authors to benefit the reader, the posted materials are not copyedited and are the sole responsibility of the authors, so questions or comments should be addressed to the corresponding author.

ciy003_suppl_Supplementary_Material_1

ciy003_suppl_Supplementary_Material_2

ciy003_suppl_Supplementary_Material_3

ciy003_suppl_Supplementary_Material_4

ciy003_suppl_Supplementary_Material_5

## Notes

***Disclaimer.*** The views, opinions, assumptions or any other information set out in this article should not be attributed to the Bill & Melinda Gates Foundation and the Task Force for Global Health or any person connected with them.

***Financial support.*** The authors gratefully acknowledge funding of the Neglected Tropical Diseases Modelling Consortium by the Bill & Melinda Gates Foundation in partnership with the Task Force for Global Health (award number OPP1053230).

***Supplement sponsorship.***  This article appears as part of the supplement "Reaching the 2020 Goals for Nine Neglected Tropical Diseases," sponsored by the NTD Modelling Consortium.

***Potential conflicts of interest.*** All authors: No reported conflicts of interest. All authors have submitted the ICMJE Form for Disclosure of Potential Conflicts of Interest. Conflicts that the editors consider relevant to the content of the manuscript have been disclosed.
