# Supplementary material for: Are Alternative Strategies Required to Accelerate the Global Elimination of Lymphatic Filariasis? Insights From Mathematical Models
Source: Clin Infect Dis. 2018 Jun 1;66(Suppl 4):S260–6. doi: 10.1093/cid/ciy003 (PMC5982795; doi:10.1093/cid/ciy003)
Supplement: ciy003_suppl_Supplementary_Material_1 [file ciy003_suppl_supplementary_material_1.pdf]

EPIFIL model description

Prepared by Morgan Smith and Edwin Michael
August 28, 2017

Description of the mathematical model

We employed a genus specific mosquito-vectored transmission model of LF to carry out the modelling work in this study [1-7]. Briefly, the state variables of this hybrid coupled partial differential and differential equation model vary over age (*a*) and/or time (*t*), representing changes in the pre-patent worm burden per human host $(P(a,t))$, adult worm burden per human host $(W(a,t))$, the microfilariae (mf) level in the human host modified to reflect infection detection in a 1 mL blood sample $(M(a,t))$, the average number of infective L3 larval stages per mosquito (*L*), and a measure of immunity $(I(a,t))$ developed by human hosts against L3 larvae. The state equations comprising this model are:

$$\frac{\partial P}{\partial t}+\frac{\partial P}{\partial a}=\lambda\frac{V}{H}h(a)\Omega(a,t)-\mu P(a,t)-\lambda\frac{V}{H}h(a)\Omega(a,t-\tau)\zeta$$
$$\frac{\partial W}{\partial t}+\frac{\partial W}{\partial a}=\lambda\frac{V}{H}h(a)\Omega(a,t-\tau)\zeta-\mu W(a,t)$$
$$\frac{\partial M}{\partial t}+\frac{\partial M}{\partial a}=\alpha s\phi[W(a,t),k]W(a,t)-\gamma M(a,t)$$
$$\frac{\partial I}{\partial t}+\frac{\partial I}{\partial a}=W_T(a,t)-\delta I(a,t)$$
$$\frac{dL}{dt}=\lambda\kappa g\int\pi(a)(1-f[M(a,t),k])da-(\sigma+\lambda\psi_1)L$$
$$L^*=\frac{\lambda\kappa g\int\pi(a)(1-f[M(a,t)],k)da}{\sigma+\lambda\psi_1}$$

The above equations involve partial derivatives of four state variables (*P* - pre-patent worm load; *W* - adult worm load; *M* - microfilaria intensity; *I* - immunity to acquiring new infection due to the pre-existing total worm load where $W_T = W(a,t) + P(a,t)$). Given the faster time scale of infection dynamics in the vector compared to the human host, the infective L3-stage larval density in mosquito population is modelled by an ordinary differential equation essentially reflecting the

significantly faster time-scale of the infection dynamics in the vector hosts. This allows us to make the simplifying assumption that the density of infective stage larvae in the vector population reaches a dynamic equilibrium (denoted by L^*) rapidly in comparison with the other state variables [1, 2, 5, 8, 9]. This basic coupled immigration-death structure of the model as well as its recent extensions has been extensively discussed previously[1-3, 5, 8, 9]. The effects of worm patency are captured by considering that at any time *t*, human individuals of age, *a*, less than or equal to the pre-patency period, *τ*, will have no adult worms or Mf, and the rate at which pre-patent worms survive to become adult worms in these individuals at $a > \tau$ is given by $\zeta = \exp(-\mu\tau)$. The term, $f[M(a,t),k]$, denotes the functional form relating the L3-stage larvae uptake and development in the vector population, which is well known to depend on the genus of the transmitting mosquito vector. This dependency is captured by the following vector genus-specific expressions:

$$f[M(a,t),k] = \left[\frac{2}{\left[1+\frac{M(a,t)}{k}\left(1-\exp\left[-\frac{r}{\kappa}\right]\right)\right]^{k}} - \frac{1}{\left[1+\frac{M(a,t)}{k}\left(1-\exp\left[-\frac{2r}{\kappa}\right]\right)\right]^{k}} \right]$$ for mosquitoes of *Anopheline* genus;

$$f[M(a,t),k] = \left(1+\frac{M(a,t)}{k}\left(1-\exp\left[-\frac{r}{\kappa}\right]\right)\right)^{-k}$$ for mosquitoes of *Culicine* genus.

In the above, $k[=k_0 + k_{Lin}M]$ is the shape parameter of the negative binomial distribution on the Mf uptake whereas *r* and *κ* are respectively the rate of initial increase and the maximum level of L3 larvae. See Table S1.1 for the description of all the model parameters and functions.

Table S1.1 - Description the basic LF model parameters and functions used in the model.

Parameter	Definition (*units*)	Range	Refs
λ	Number of bites per mosquito (*per month*)	[5, 15]	[1, 2, 5, 10, 11]
τ	Pre-patency period	[1, 9]	[12]
s	Proportion of female worms	0.5	-
μ	The worm mortality rate (*per month*)	[0.008, 0.018]	[1, 2, 5, 13-16]
α	Production rate of microfilariae per worm (*per month*)	[0.25, 1.5]	[1, 2, 5, 17]
γ	The death rate of the microfilariae (*per month*)	[0.08, 0.12]	[1, 5, 15, 17]
g	Proportion of mosquitoes which pick up infection when biting an infected host	[0.251, 0.485]	[1, 5, 18]
κ	Maximum level of L3 given Mf density	[3, 5]	[1, 5]
k₀	The basic location parameter of negative binomial distribution used in aggregation parameter ($k = k_0 + k_{Lin}M$)	[0.000036, 0.000775]	[1, 5, 19, 20]
δ	Immunity waning rate (*per month*)	[0.001, 0.01]	[1, 5]
V/H	Ratio of number of vector to hosts	$MBR^{\#} / \lambda$	data
k_{Lin}	The linear rate of increase in the aggregation parameter defined above	[0.00000024, 0.282]	[1, 5, 19, 20]
σ	Death rate of mosquitoes (*per month*)	[1.5, 8.5]	[1, 5, 20]
ψ_1	Proportion of L3 leaving mosquito per bite	[0.1, 0.8]	[17]
ψ_2	The establishment rate[1]	[0.00003, 0.00364]	[1, 2, 5, 21]
H_{Lin}	A threshold value used in $h(a)$ to adjust the rate at which individuals of age *a* are bitten: linear rise from 0 at age zero to 1 at age H_{lin} in years. $h(a) = a / H_{Lin}$ for $a < H_{Lin}; h(a) = 1$ for $a \geq H_{Lin}$	[240, 360] months	[1, 5, 9]
r	Gradient of Mf uptake[2]	[0.04, 0.25]	[1, 5]
c	Strength of acquired immunity	[0.015, 0.025]	[1, 5]
I_C	Strength of immunosuppression[3]	[0.5, 5.5]	[1, 5]
S_C	Slope of immunosuppression function[4] (*per worm/month*)	[0.01, 0.20]	[1, 5]
MDA drug-related parameters			
ω	Worm killing efficacy (instantaneous)	dependent on drug regimen	[3]
ε	Microfilariae killing efficacy (instantaneous)	dependent on drug regimen	[3]
δ_{reduc}	Reduction in the worm's fecundity over a period of time p	dependent on drug regimen	[3]
p	A time period during which the drug remains efficacious in reducing the fecundity of the surviving adult worms	dependent on drug regimen	[3]
C	Percentage of the population administered the drug	data	data
**Implementing vector control (VC)** such as IVM modifies the V/H ($= MBR/\lambda$)			
MBR_{VC}	$MBR_{VC} = MBR_0 \exp[a_1 t]$, with $a_1 < 0$ for $\forall t$ when VC is ON, otherwise $a_1 > 0$.	data and estimates	[19, 20]

Description	Mathematical expressions of the functions	Parameters	
Probability that an individual is of age *a* $\pi(a)$	$\pi(a) = A_0 \exp[-B_0 a]$	Human age *a* in month, A_0 and B_0 estimated from country demographic data	1, 5, 9
Larvae establishment rate (modified by acquired immunity) Ω(a,t)	$L^* \psi_1 \psi_2 g_1(I) g_2(W_T)$	ψ_1 - proportion of L3 leaving mosquito per bite; ψ_2 - the establishment rate[1]	-
Adult worm mating probability $\phi(W,k)$	$1 - \left(1 + \frac{W}{2k}\right)^{-(1+k)}$	*k* – negative binomial aggregation parameter	2, 5, 22
Immunity to larval establishment $g_1(I)$	$\frac{1}{1+cI}$	*c* – strength of immunity to larval establishment	1, 5
Host immunosuppression $g_2(W_T)$	$\frac{1+I_C S_C W_T}{1+S_C W_T}$	I_C – strength of immunosuppression; S_C – slope of immunosuppression	1, 5

[1]The proportion of L3-stage larvae infecting human hosts that survive to develop into adult worms[2].
[2]The gradient of Mf uptake *r* is a measure of the initial increase in the infective L3 larvae uptake by vector as *M* increases from 0[2, 9].
[3] The facilitated establishment rate of adult worms due to parasite-induced immunosuppression in a heavily infected human host
[4] The initial rate of increase by which the strength of immunosuppression is achieved as *W* increases from 0[23].
Note MBR (monthly biting rate) serves as an input to initialize the model, measured as mosquito bites per person per month, the value of which may be obtained from entomological surveys conducted in study sites. In the absence of the observed MBR value, the model has been adapted to estimate it from the community-level Mf prevalence data.

Parameter quantification and simulation methods for this study

Typically, we employed the Bayesian Melding (BM) procedure to calibrate and estimate the LF models from field data, as outlined in detail in our previous work [1, 2, 5, 6]. In brief, we begin the procedure by first using the known or assignment of a uniform range for parameter values to generate distributions of parameter priors. We then randomly sample with replacement from these prior distributions to generate 200,000 parameter vectors, which are then run using the annual biting rate (ABR) values, if given, for a site to generate model outputs. The model outputs are then melded with age-stratified Mf prevalence data by calculating binomial log-likelihoods for each parameter vector. In the resampling step of the BM method, a Sampling-Importance-Resampling (SIR) algorithm is used to perform 500 draws with replacement from the pool of parameter vectors generated as above, with probabilities proportional to their relative log likelihood values. This step selects the parameter vectors which best describe the given mf age-prevalence data. These resampled parameter vectors are then used to generate distributions of variables of interest from the fitted model (eg. age-prevalence curves, worm breakpoints and infection trajectories following treatments).

In the present work, we extended the above procedure to provide simulations for the scenarios which were chosen to reflect pre-intervention conditions in each of three LF endemic regions of the world investigated here. The explicit aim was to generate 10,000 parameter vectors which resulted in reproducing the range of overall baseline mf prevalence values specified for each region (i.e. 1-15% for Indian scenarios, 1-40% for African scenarios, and 1-70% for PNG scenarios). The BM approach was adapted to accomplish this task by essentially sampling parameter vectors along with ABR values that ranged between 1,000 and 50,000 bites per person per year that were able to produce these overall mf prevalence values, while discarding those that produced prevalence values outside of the range. To ensure that a uniform posterior distribution of prevalences was generated by this modelling approach, a check

was made to confirm that the modeled prevalences fell into uniform bin sizes constructed for each prevalence range. In addition to varying the range of prevalences modeled by region, the demographic parameters A_0 and B_0 also differ by region according to relevant population age structures (Table S1.1). For the African scenarios, demographic parameters used here reflected those describing the population age structure of Tanzania, while the Indian and PNG scenarios shared demographic parameters coming from India's population age structure. Additionally, the mf uptake function $f[M(a,t),k]$ is specific to the genus of mosquito responsible for transmission in a particular region. Here, the *Culicine* formulation was used for the Indian scenarios, and the *Anopheline* formulation was used for the PNG and African scenarios.

Modeling intervention by mass drug administration

Intervention by mass drug administration was modeled based on the assumption that anti-filarial treatment with a combination drug regimen acts by killing certain fractions of the populations of adult worms and microfilariae instantly after the drug administration. These effects are incorporated into the basic model by calculating the population sizes of worms and microfilariae as follows:

$$\left.\begin{aligned} P(a,t+dt) &= (1-\omega C)P(a,t) \\ W(a,t+dt) &= (1-\omega C)W(a,t) \\ M(a,t+dt) &= (1-\varepsilon C)M(a,t) \end{aligned}\right\} \quad \text{at time } t = T_{MDA_i}$$

where *dt* is a short time period (here, 1 month) since the time T_{MDAi} when the ith MDA was administered. During this short time interval, a given proportion of adult worms and microfilariae are instantly removed. The parameters ω and ε are drug killing efficacy rates for the two life stages of the parasite (worm and microfilariae, respectively), while the parameter *C* represents the MDA coverage. MDA coverage is assumed to apply randomly to the population of the appropriate age for taking the particular drug. Treatment non-compliance is not considered. Apart from instantaneous killing of microfilariae, the drug continues to kill the newly reproduced Mf by any surviving adult worms at a rate δ_{reduc} for a period of time, *p*. We model this effect as follows:

$$\frac{\partial M(a,t)}{\partial t} + \frac{\partial M(a,t)}{\partial a} = (1-\delta_{reduc}C)s\alpha\phi(W(a,t),k)W(a,t) - \gamma M(a,t), \quad \text{for } T_{MDA_i} < t \le T_{MDA_i} + p$$

We simulate LF intervention by running the model with the fixed-values of the four drug-related parameters (ω, ε, δ_{reduc}, and p) for MDA coverage levels specified in each scenarios. The first MDA round is implemented into the model by simulating its effects on the population sizes of adult worm and microfilariae from the baseline fits, with subsequent MDA rounds implemented as annual or biannual events acting on the sequential parasite loads arising from each applied MDA.

References

1. Gambhir, M. & Michael, E. Complex ecological dynamics and eradicability of the vector borne macroparasitic disease, lymphatic filariasis. *PLoS One* **3**, e2874 (2008).

2. Gambhir, M. *et al*. Geographic and ecologic heterogeneity in elimination thresholds for the major vector-borne helminthic disease, lymphatic filariasis. *BMC biology* **8**, 1 (2010).

3. Michael, E. *et al*. Mathematical modelling and the control of lymphatic filariasis. *The Lancet infectious diseases* **4**, 223-234 (2004).

4. Michael, E., Malecela-Lazaro, M. N., Kabali, C., Snow, L. C. & Kazura, J. W. Mathematical models and lymphatic filariasis control: endpoints and optimal interventions. *Trends Parasitol.* **22**, 226-233 (2006).

5. Singh BK and Bockarie MJ and Gambhir M and Siba PM and Tisch DJ and Kazura J and others. in *Sequential Modelling of the Effects of Mass Drug Treatments on Anopheline-Mediated Lymphatic Filariasis Infection in Papua New Guinea* (PLoS One, 2013).

6. Singh, B. K. & Michael, E. Bayesian calibration of simulation models for supporting management of the elimination of the macroparasitic disease, Lymphatic Filariasis. *Parasit vectors* **8**, 1-26 (2015).

7. Michael, E. & Singh, B. K. Heterogeneous dynamics, robustness/fragility trade-offs, and the eradication of the macroparasitic disease, lymphatic filariasis. *BMC medicine* **14**, 1 (2016).

8. Chan, M. S. *et al*. Epifil: a dynamic model of infection and disease in lymphatic filariasis. *Am. J. Trop. Med. Hyg.* **59**, 606-614 (1998).

9. Norman, R. *et al*. EPIFIL: the development of an age-structured model for describing the transmission dynamics and control of lymphatic filariasis. *Epidemiol. Infect.* **124**, 529-541 (2000).

10. Rajagopalan, P. Population dynamics of culex pipiens fatigans, the filariasis vector, in pondicherry: influence of climate and environment. *Proc Ind Nat Science Acad B* **46**, 745-752 (1980).

11. Subramanian, S., Manoharan, A., Ramaiah, K. D. & Das, P. K. Rates of acquisition and loss of Wuchereria bancrofti infection in Culex quinquefasciatus. *Am. J. Trop. Med. Hyg.* **51**, 244-249 (1994).

12. Scott, A. L. & Nutman, T. Lymphatic-dwelling filariae. *Lymphatic filariasis.*, 5-39 (2000).

13. Vanamail, P., Subramanian, S., Das, P. K., Pani, S. P. & Rajagopalan, P. K. Estimation of fecundic life span of Wuchereria bancrofti from longitudinal study of human infection in an endemic area of Pondicherry (south India). *Indian J. Med. Res.* **91**, 293-297 (1990).

14. Evans, D. B., Gelband, H. & Vlassoff, C. Social and economic factors and the control of lymphatic filariasis: a review. *Acta Trop.* **53**, 1-26 (1993).

15. Ottesen, E. & Ramachandran, C. Lymphatic filariasis infection and disease: control strategies. *Parasitology Today* **11**, 129-130 (1995).

16. Vanamail, P. *et al*. Estimation of the fecund life span of Wuchereria bancrofti in an endemic area. *Trans. R. Soc. Trop. Med. Hyg.* **90**, 119-121 (1996).

17. Hairston, N. G. & de Meillon, B. On the inefficiency of transmission of Wuchereria bancrofti from mosquito to human host. *Bull. World Health Organ.* **38**, 935-941 (1968).

18. Subramanian, S. *et al*. The relationship between microfilarial load in the human host and uptake and development of Wuchereria bancrofti microfilariae by Culex quinquefasciatus: a study under natural conditions. *Parasitology* **116**, 243-255 (1998).

19. Subramanian, S., Pani, S., Das, P. & Rajagopalan, P. Bancroftian filariasis in Pondicherry, south India: 2. Epidemiological evaluation of the effect of vector control. *Epidemiol. Infect.* **103**, 693-702 (1989).

20. Das, P. *et al*. Bancroftian filariasis in Pondicherry, south India–epidemiological impact of recovery of the vector population. *Epidemiol. Infect.* **108**, 483-493 (1992).

21. Ho, B. C. & Ewert, A. Experimental transmission of filarial larvae in relation to feeding behaviour of the mosquito vectors. *Trans. R. Soc. Trop. Med. Hyg.* **61**, 663-666 (1967).

22. May, R. M. Togetherness among schistosomes: its effects on the dynamics of the infection. *Math. Biosci.* **35**, 301-343 (1977).

23. Duerr, H., Dietz, K. & Eichner, M. Determinants of the eradicability of filarial infections: a conceptual approach. *Trends Parasitol.* **21**, 88-96 (2005).
