# Supplementary material for: Are Alternative Strategies Required to Accelerate the Global Elimination of Lymphatic Filariasis? Insights From Mathematical Models
Source: Clin Infect Dis. 2018 Jun 1;66(Suppl 4):S260–6. doi: 10.1093/cid/ciy003 (PMC5982795; doi:10.1093/cid/ciy003)
Supplement: ciy003_suppl_Supplementary_Material_2 [file ciy003_suppl_supplementary_material_2.pdf]

LYMFASIM model description

Wilma A. Stolk, Periklis Kontoroupis, Anneke S. de Vos

August 30, 2017

Description of the mathematical model

LYMFASIM [1, 2] is an individual-based model for lymphatic filariasis, part of a generalized framework (WORMSIM) for modelling transmission and control of helminth infections in humans [3, 4]. LYMFASIM simulates the life histories of individual worms and their transmission from person to person mediated by a cloud of vectors. Furthermore, the model combines two simulation methods, a stochastic micro-simulation to calculate the life events of individual persons and their inhabitant parasites and a deterministic simulation of the vector population. Several publications describe previous applications of the model to support decision making on control and elimination of lymphatic filariasis (LF) [1, 2, 5-13].

Parameter quantification and simulation methods for this study

The model in this study was parameterized for the India and Africa setting based on previous successful implementations [2, 8, 12]. A key difference between the two was the inclusion of anti-L3 treatment immunity for India, which influenced the parameter quantification [8]. We used the same parameter quantifications in the current study for each region for all parameters, except for three models parameters that determine the local transmission conditions, i.e.: the monthly biting rate, the exposure heterogeneity and the external force of infection. We varied these three parameters between simulation runs, in order to cover the entire region-specific ranges of pre-control mf prevalence levels. Variation in the first two, allowed model predictions to reach target mf prevalence level, whereas the last parameter was included to stabilize the transmission dynamics during pre-control (important for the low endemic situations).

The monthly biting rate and exposure heterogeneity were sampled from a certain parameter space, as depicted in figure S2.1. This space was confined by three multivariate distributions linked to each other with some user-selected weights. Parameters in these distributions were estimated from on-going project results, whereby different areas in the parameter

space are linked to various prevalence levels. Furthermore, in order to produce a near uniform distribution at baseline, weights linked to each distribution were adjusted accordingly in order not to oversample one area versus another. The first panel in figure S2.1 (a) depicts the parameter space for the Indian setting and the second panel (b) the parameter space for Africa. It is noted that for both settings, the external force was sampled from a uniform distribution with minimum at 0.001 and maximum at 0.35. The external force of infection was simulated to decrease during the control period according to the MDA coverage implemented.

Figure S2.1: The parameter space utilized for the two different regions (India and Africa) to achieve the required baseline (t=0) mf, different colors correspond to different prevalence levels, symbols illustrate mf prevalence at different time points.

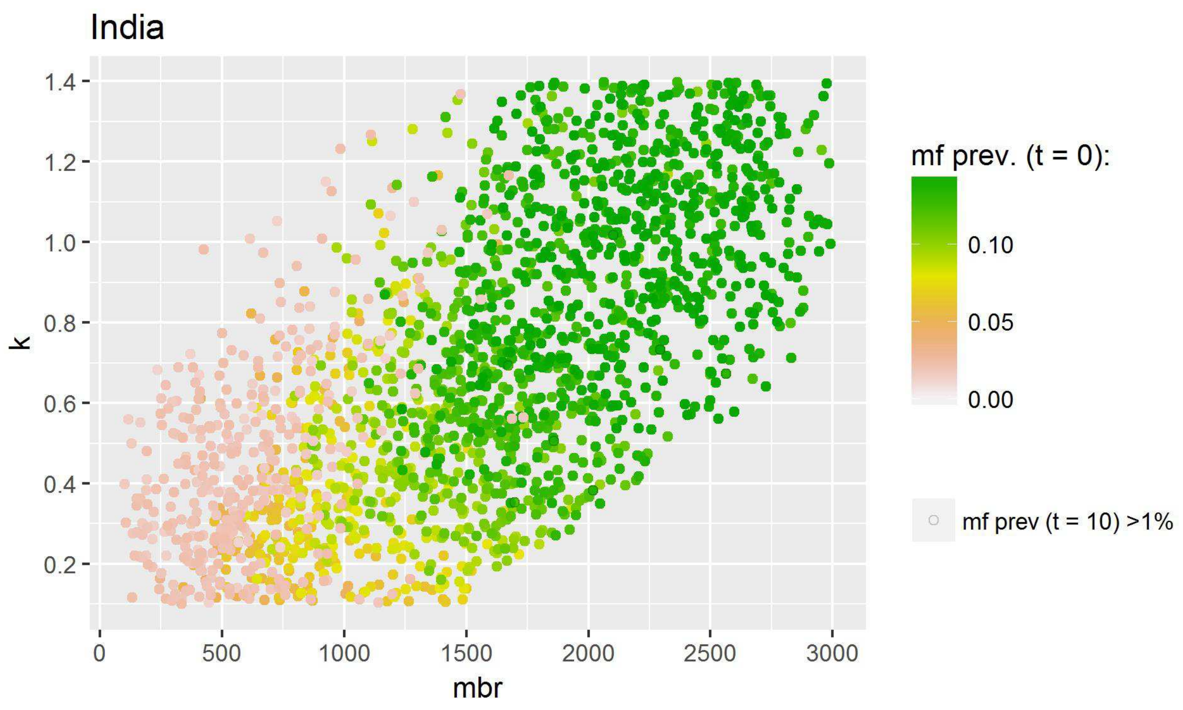

(a) The parameter space used for India, treatment naïve scenarios

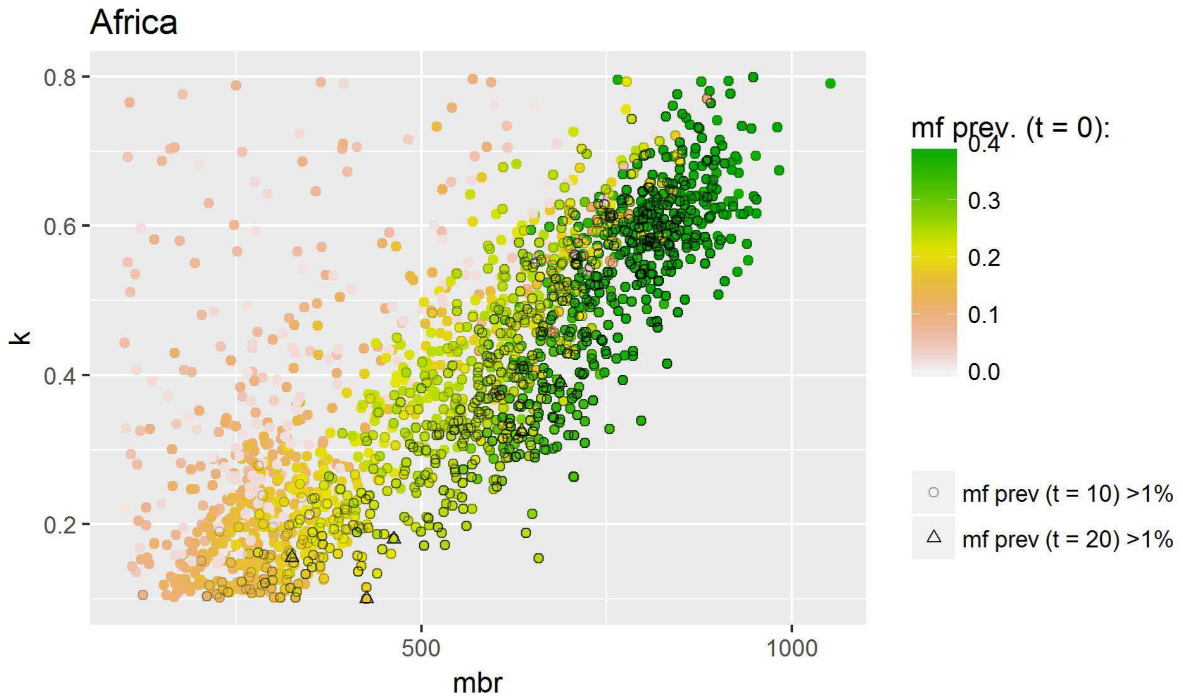

(b) The parameter space used for Africa, treatment naïve scenarios

Modelling intervention by mass drug administration

Parameters related to the local history of control and future treatment scenario were varied between depending on setting type (TN, RS, F1, F2) and future treatment scenarios. Relevant parameters are: timing of MDA rounds, efficacy of employed treatment regimen, and the achieved coverage and fraction excluded from treatment per round. Modelling intervention by mass drug administration at different scenario types, was based on various levels of coverage and the inclusion of systematically non-adherent individuals.

The primary characteristic of a round of MDA is the coverage (fraction of the population treated). A difficulty is that compliance patterns tend to vary by age and sex, sometimes imposed due to exclusion criteria for treatment, and that compliance to treatment differs from person to person. In this study, therefore, we assume that an individual chance of participation per treatment round is defined by three mechanisms. Firstly, a fraction of people will never participate in MDA (e.g. systematic refusal, relate to chronic illness). Secondly, the model allows the relative compliance to vary between age and sex groups; this mechanisms captures transient contra-indications for MDA (e.g. exclusion of young children and pregnant women) and other age- and sex-related behavioural factors driving participation in MDA. Thirdly, each individual has a personal inclination to participate in MDA, which is considered as a lifelong property. A stochastic process eventually defines for each individual whether or not he/she is treated in a given round, depending on the calculated probability. See the previously published formal description of WORMSIM for more information.

Table S2.1 presents the assumed coverage per scenario type and the proportion of the population missed during treatment. We assume that the low coverage in failure 1 and 2 scenario's was associated with a high or very high proportion of the population never taking treatment. Table S2.2 gives the relative compliance by age and sex.

Table S2.1: Parametrization of individual compliance after multiple rounds of treatment

Scenario type	Coverage (% out of total population)	Population systematically excluded from treatment (% out of total population)
Normal (TN, RS)	65/80	5
Failure 1 (F1)	50	25
Failure 2 (F2)	30	60

Table S2.2: Relative compliance by age and sex

age-group	Relative compliance males	Relative compliance females
0-1*	0	0
2*-9	0.75	0.75
10-14	0.80	0.70
15-19	0.80	0.74
20-29	0.70	0.65
30-49	0.75	0.70
50-59	0.75	0.70
60-99	0.80	0.75

* Young children are excluded from treatment, hence their relative compliance is 0. The minimum age for treatment varies between treatment regimen.

References

1. Plaisier AP, Subramanian S, Das PK, et al. The LYMFASIM simulation program for modeling lymphatic filariasis and its control. Methods of Information in Medicine **1998**; 37: 97-108.
2. Subramanian S, Stolk WA, Ramaiah KD, et al. The dynamics of *Wuchereria bancrofti* infection: a model-based analysis of longitudinal data from Pondicherry, India. Parasitology **2004**; 128(Pt 5): 467-82.

3. Coffeng LE, Bakker R, Montresor A, de Vlas SJ. Feasibility of controlling hookworm infection through preventive chemotherapy: a simulation study using the individual-based WORMSIM modelling framework. Parasit Vectors **2015**; 8: 541.
4. Stolk WA, Walker M, Coffeng LE, Basanez MG, de Vlas SJ. Required duration of mass ivermectin treatment for onchocerciasis elimination in Africa: a comparative modelling analysis. Parasit Vectors **2015**; 8(1): 552.
5. Stolk WA, Subramanian S, Oortmarssen GJ, Das PK, Habbema JDF. Prospects for elimination of bancroftian filariasis by mass drug treatment in Pondicherry, India: a simulation study. J Infect Dis **2003**; 188(9): 1371-81.
6. Stolk WA, De Vlas SJ, Habbema JDF. Anti-*Wolbachia* treatment for lymphatic filariasis. Lancet **2005**; 365(9477): 2067-8.
7. Stolk WA, de Vlas SJ, Habbema JD. Advances and challenges in predicting the impact of lymphatic filariasis elimination programmes by mathematical modelling. Filaria J **2006**; 5(1): 5.
8. Stolk WA, de Vlas SJ, Borsboom GJ, Habbema JD. LYMFASIM, a simulation model for predicting the impact of lymphatic filariasis control: quantification for African villages. Parasitology **2008**; 135(13): 1583-98.
9. Stolk WA, ten Bosch QA, de Vlas SJ, Fischer PU, Weil GJ, Goldman AS. Modeling the impact and costs of semiannual mass drug administration for accelerated elimination of lymphatic filariasis. PLoS Negl Trop Dis **2013**; 7(1): e1984.
10. Stolk WA, Stone C, de Vlas SJ. Modelling lymphatic filariasis transmission and control: modelling frameworks, lessons learned and future directions. Adv Parasitol **2015**; 87: 249-91.
11. Jambulingam P, Subramanian S, de Vlas SJ, Vinubala C, Stolk WA. Mathematical modelling of lymphatic filariasis elimination programmes in India: required duration of mass drug administration and post-treatment level of infection indicators. Parasit Vectors **2016**; 9: 501.
12. Irvine MA, Stolk WA, Smith ME, et al. Effectiveness of a triple-drug regimen for global elimination of lymphatic filariasis: a modelling study. Lancet Infect Dis **2017**; 17(4): 451-8.
13. Smith ME, Singh BK, Irvine MA, et al. Predicting lymphatic filariasis transmission and elimination dynamics using a multi-model ensemble framework. Epidemics **2017**; 18: 16-28.
