# Supplementary material for: Are Alternative Strategies Required to Accelerate the Global Elimination of Lymphatic Filariasis? Insights From Mathematical Models
Source: Clin Infect Dis. 2018 Jun 1;66(Suppl 4):S260–6. doi: 10.1093/cid/ciy003 (PMC5982795; doi:10.1093/cid/ciy003)
Supplement: ciy003_suppl_Supplementary_Material_3 [file ciy003_suppl_supplementary_material_3.pdf]

TRANSFIL model description

Joaquín M. Prada and Panayiota Touloupou

August 29, 2017

Description of the mathematical model

The mathematical model of lymphatic filariasis transmission TRANSFIL has been described in detail in Irvine *et al.* [1] and more recently in Smith *et al.* [2], so here we provide a brief overview with the updated aspects of the model. TRANSFIL is an individual−based model of lymphatic filariasis infection in human populations, with each host having their own adult worm and microfilariae burden, as well as bite risk and treatment history. The key aspects relevant to this manuscript are the different scenarios, the drug treatments, the compliance and the external importation of cases.

We defined 3 broad scenarios tied to three regions, India (IND), Africa (AFR) and Papua New Guinea (PNG). The main differences among these three regions are the dominant vector species, *Culex* for IND and *Anopheles* for AFR and PNG and the relative ranges of prevalence that we considered, based on the information available [3, 4, 5, 6, 7].

Parameter quantification and simulation methods for this study

To generate the require range of mf prevalences, we varied two parameters of the model, the vector to host ratio (V/H) and the average population bite risk (k), using parameter sets from a range of plausible values based on previously analysed data for each of the three regions [1, 2, 8]. The graphical representation of the possible values is shown in Figure S2.1.

For stochastic models it is essential that the model includes an importation rate, otherwise the equilibrium distribution (steady state) that is used

Figure S2.1: The shaded areas correspond to the possible values of V/H and k for which the required baseline (t=0) mf prevalence levels are achieved for each of the three regions.

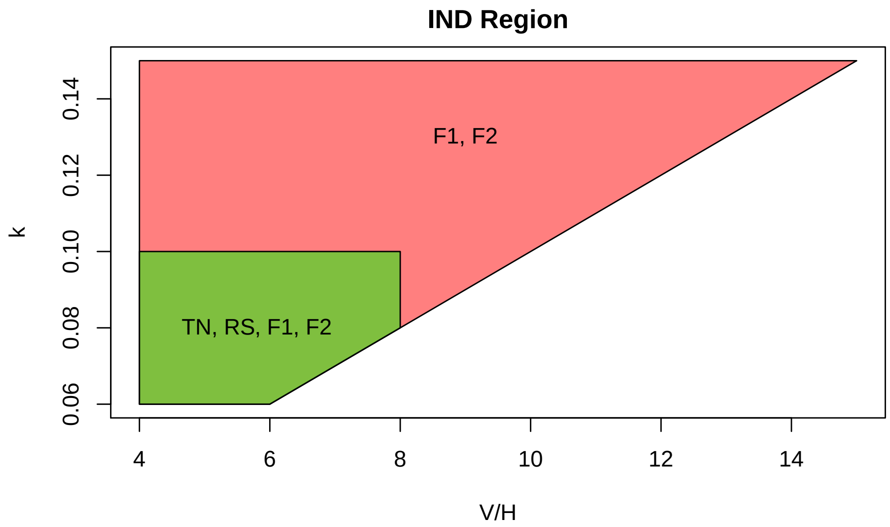

(a) Range of baseline mf prevalence levels for the IND region: $1\% - 15\%$.

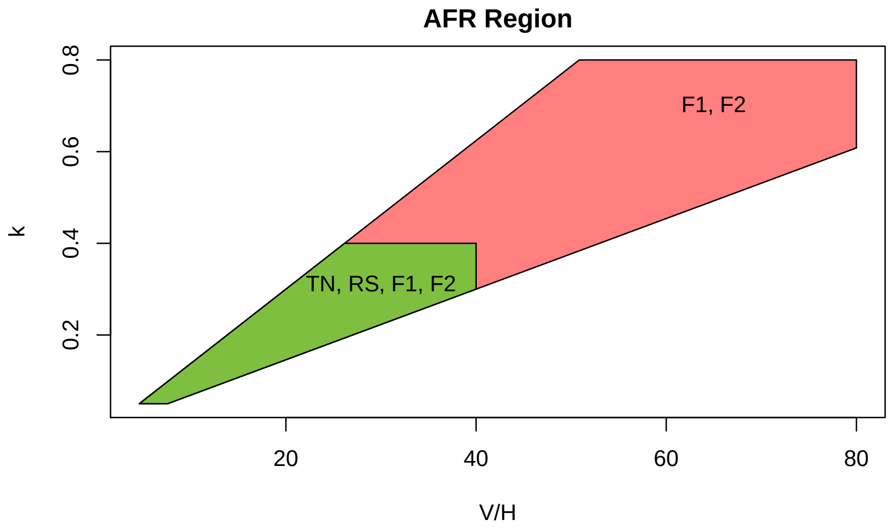

(b) Range of baseline mf prevalence levels for the AFR region: $1\% - 40\%$.

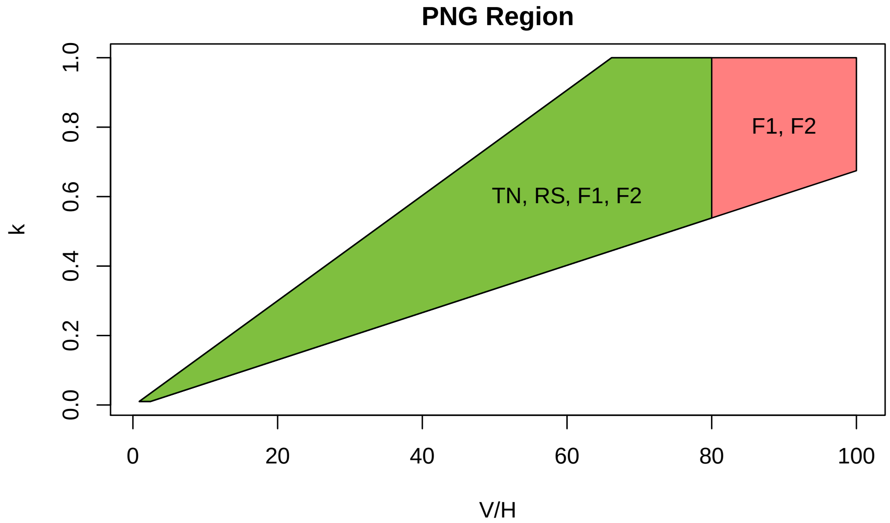

(c) Range of baseline mf prevalence levels for the PNG region: 1% – 70%.

as the starting point of the simulations is just the degenerate distribution where no-one is infected. The importation rate does not need to be large, in fact it should not be driving the infection. For this LF study we used a random number drawn from a uniform distribution with minimum 0 and maximum 0.001 (max 1/1000 infections per month). The interventions reduce the prevalence over time, and therefore as year pass, the importation rate decreases based on some pilot simulations.

Modelling intervention by mass drug administration

The main three aspects that differentiate the treatments are the mortality caused in adults worms and microfilariae, and the sterilization of the surviving adults. These are summarized in the main manuscript.

We modelled individuals' compliance after multiple rounds of treatment based on the paper by Griffin *et al.* [9], where a parameter ρ is used to model the probability of an individual making the same decision than in the previous round of treatment. This approach is different from the other models in this manuscript, and they have been compared recently by Dyson *et al.* [10]. We selected the ρ values for our simulations by using maximum

likelihood to minimize the difference in the distribution of the number of rounds attended after ten treatments when compared to the semi-systematic approach taken by Stolk *et al.* [11], which is defined by the coverage and a proportion of population systematically missed. This is described in detail by Dyson *et al.* [10] and we summarized the results in Table S2.1.

Table S2.1: Parametrization of individual compliance after multiple rounds of treatment.

Scenario type	Coverage (%)	Population missed (%)	ρ
Normal	65/80	5	0.35
Failure 1	50	20	0.66
Failure 2	30	50	0.94

References

[1] Irvine, M.A., Reimer, L.J., Njenga, S.M., Gunawardena, S., Kelly-Hope, L., Bockarie, M., Hollingsworth, T.D.: Modelling strategies to break transmission of lymphatic filariasis - aggregation, adherence and vector competence greatly alter elimination. Parasites & Vectors **8**(1), 547 (2015)

[2] Smith, M.E., Singh, B.K., Irvine, M.A., Stolk, W.A., Subramanian, S., Hollingsworth, T.D., Michael, E.: Predicting lymphatic filariasis transmission and elimination dynamics using a multi-model ensemble framework. Epidemics **18**, 16–28 (2017)

[3] Das, P.K., Manoharan, A., Subramanian, S., Ramaiah, K.D., Pani, S.P., Rajavel, A.R., Rajagopalan, P.K.: Bancroftian filariasis in Pondicherry, South India epidemiological impact of recovery of the vector population. Epidemiology and Infection **108**(3), 483–493 (1992)

[4] Njenga, S.M., Wamae, C.N., Njomo, D.W., Mwandawiro, C.S., Molyneux, D.H.: Impact of two rounds of mass treatment with diethylcarbamazine plus albendazole on Wuchereria bancrofti infection and the sensitivity of immunochromatographic test in Malindi, Kenya. Transactions of the Royal Society of Tropical Medicine and Hygiene **102**(10), 1017–1024 (2008)

[5] Rajagopalan, P.K., Das, P.K., Subramanian, S., Vanamail, P., Ramaiah, K.D.: Bancroftian filariasis in Pondicherry, South India: 1. Pre-control epidemiological observations. Epidemiology and Infection **103**(3), 685–692 (1989)

[6] Singh, B.K., Bockarie, M.J., Gambhir, M., Siba, P.M., Tisch, D.J., Kazura, J., Michael, E.: Sequential modelling of the effects of mass drug treatments on anopheline-mediated lymphatic filariasis infection in Papua New Guinea. PLoS One **8**(6), e67004 (2013)

[7] Subramanian, S., Pani, S.P., Das, P.K., Rajagopalan, P.K.: Bancroftian filariasis in Pondicherry, South India: 2. Epidemiological evaluation of the effect of vector control. Epidemiology and Infection **103**(3), 693–702 (1989)

[8] Irvine, M.A., Stolk, W.A., Smith, M.E., Subramanian, S., Singh, B.K., Weil, G.J., Michael, E., Hollingsworth, T.D.: Effectiveness of a triple-drug regimen for global elimination of lymphatic filariasis: a modelling study. Lancet Infectious Diseases **17**(4), 451–458 (2017)

[9] Griffin, J.T., Hollingsworth, T.D., Okell, L.C., Churcher, T.S., White, M., Hinsley, W., Bousema, T., Drakeley, C.J., Ferguson, N.M., Basez, M.-G., Ghani, A.C.: Reducing Plasmodium falciparum malaria transmission in Africa: A model-based evaluation of intervention strategies. PLOS Medicine **7**(8), 1–17 (2010)

[10] Dyson, L., Stolk, W.A., Farrell, S.H., Hollingsworth, T.D.: Measuring and modelling the effects of systematic non-adherence to mass drug administration. Epidemics **18**, 56–66 (2017)

[11] Stolk, W.A., Subramania, S., Oortmarssen, G.J., Das, P.K., Habbema, J.D.F.: Prospects for elimination of bancroftian filariasis by mass drug treatment in Pondicherry, India: a simulation study. The Journal of Infectious Diseases **188**(9), 1371–1381 (2003)
