# Supplementary material for: Are Alternative Strategies Required to Accelerate the Global Elimination of Lymphatic Filariasis? Insights From Mathematical Models
Source: Clin Infect Dis. 2018 Jun 1;66(Suppl 4):S260–6. doi: 10.1093/cid/ciy003 (PMC5982795; doi:10.1093/cid/ciy003)
Supplement: ciy003_suppl_Supplementary_Material_5 [file ciy003_suppl_supplementary_material_5.pdf]

SUPPLEMENTARY DATA 5:
DETAILED SIMULATION OUTCOMES FOR ALL SETTINGS AND SCENARIOS

Supplement to the paper "Are alternative strategies required to accelerate the global elimination of lymphatic filariasis? Insights from mathematical models" by Stolk et al (Clinical Infectious Diseases, 2017)

TABLE OF CONTENTS

Introduction to the supplement 3

List of used abbreviations 3

1. India, treatment naive 4

2. India, recent start of MDA 6

3. India, failure scenario 1 8

4. India, failure scenario 2 10

5. PNG, treatment naive 12

6. PNG, recent start of MDA 14

7. PNG, failure scenario 1 16

8. PNG, failure scenario 2 18

9. Africa, DEC+ALB (ONCHOCERCIASIS AND *LOA LOA* FREE), treatment naive 20

10. Africa, DEC+ALB (ONCHOCERCIASIS AND LOA LOA FREE), recent start of MDA 22

11. Africa, DEC+ALB (ONCHOCERCIASIS AND LOA LOA FREE), failure scenario 1 24

12. Africa, DEC+ALB (ONCHOCERCIASIS AND LOA LOA FREE) failure scenario 2 26

13. Africa, IVM+ALB (ONCHOCERCIASIS COENDEMIC LOA LOA FREE), treatment naive 28

14. Africa, IVM+ALB (ONCHOCERCIASIS COENDEMIC LOA LOA FREE), recent start MDA 30

15. Africa, IVM+ALB (ONCHOCERCIASIS COENDEMIC LOA LOA FREE), failure scenario 1 32

16. Africa, IVM+ALB (ONCHOCERCIASIS COENDEMIC LOA LOA FREE), failure scenario 2 34

17. Africa, ALB alone (LOA LOA COENDEMIC), treatment naive 36

INTRODUCTION TO THE SUPPLEMENT

In this appendix we show our detailed results on the modelled time to eradication of *Lymphatic filiriasis*, comparing currently recommended and alternative intervention strategies, per setting defined by the region and by past intervention history. Note that Africa is subdivided based on co-endemicity of *Onchocerciasis* and *Loa loa filiriasis*: Diethylcarbamazine use is strongly contra-indicated where *Onchocerciasis* is co-endemic, which is the reason why most African countries are now using ivermectin and albendazole drug regimen rather than the more effective diethylcarbamazine and albendazole combination. Where *Loa loa filiriasis* is co-endemic, both diethylcarbamazine and ivermectin are contra-indicated.

LIST OF USED ABBREVIATIONS

mf = *Lymphatic filiriasis* microfilariae.

Considered future treatment strategy options:
aMDA = annual Mass Drug Administration
bMDA = biannual Mass Drug Administration

Considered future drug regimens:
A = albendazole
DA = diethylcarbamazine and albendazole combination
IA = ivermectin and albendazole combination
IDA = ivermectin, diethylcarbamazine and albendazole combination

Considered future treatment coverages:
65 = 65% achieved
80 = 80% achieved

1. INDIA, TREATMENT NAIVE

Region: India. Parasite species: *Wuchereria bancrofti*. Vector: *Culex quinquefasciatus*. History of control: no prior MDA.

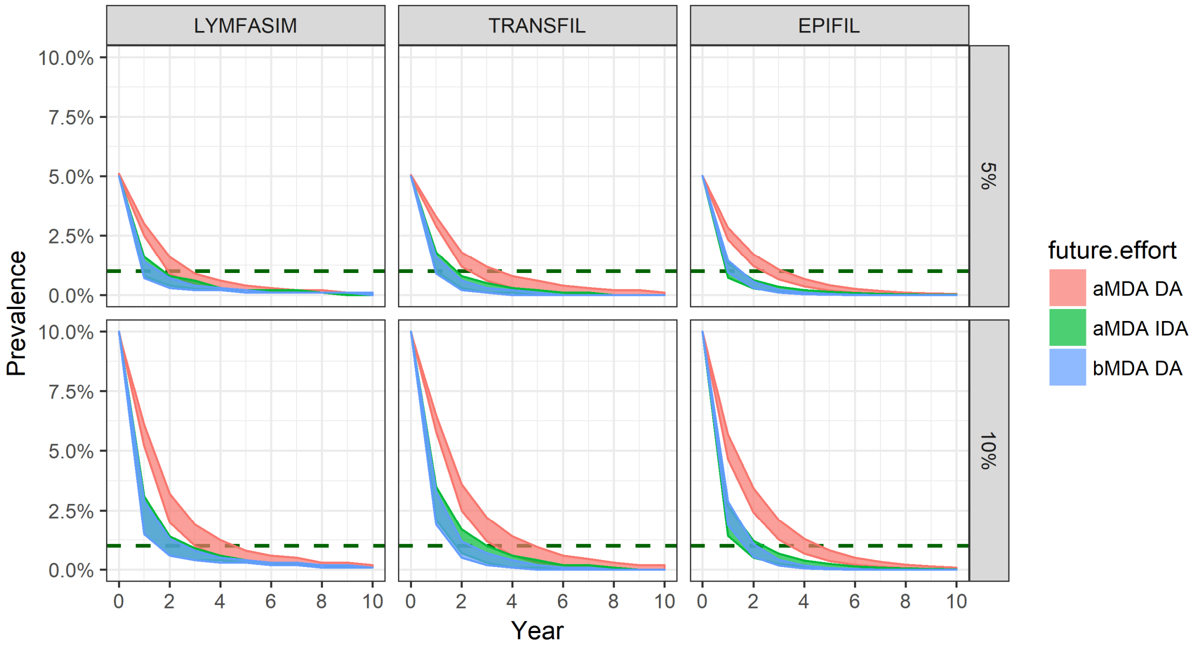

Figure 1.1. Model predicted trends in mf prevalence over time. The median mf prevalence over time is shown over the 501 runs closest to the mf prevalence given on the right. Coloured bands span the 65 to 80% intervention coverage range.

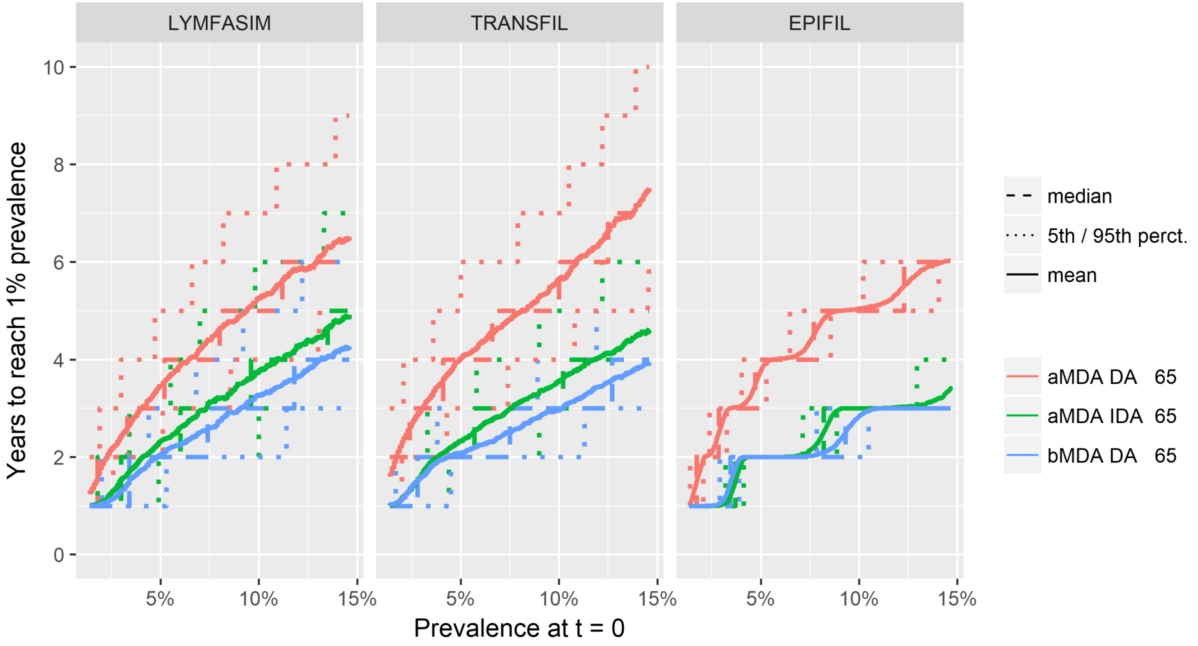

Figure 1.2. Predicted time to reach 1% mf prevalence. Shown are the moving median, the moving 5th and 95th percentile, and the moving average number of intervention years needed to bring mf prevalence below

1% (moving window size = 501. To enable calculation of the average any runs that did not achieve the 1% prevalence threshold by t=20 were set to achieving it by t=21).

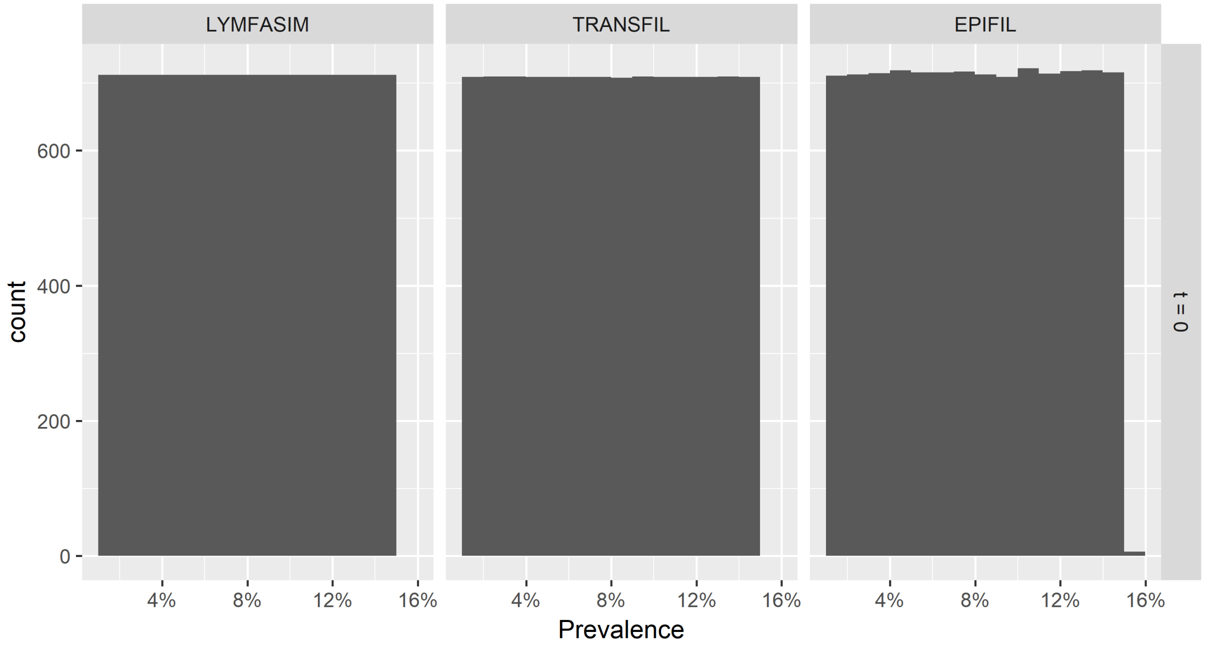

Figure 1.3. Distribution of the mf prevalence. Prevalence shown prior to any control (depending on the past intervention setting this is at $t = -10$, $t = -2$ or $t = 0$), and (if different) mf prevalence at $t = 0$. Binwidth = 1%.

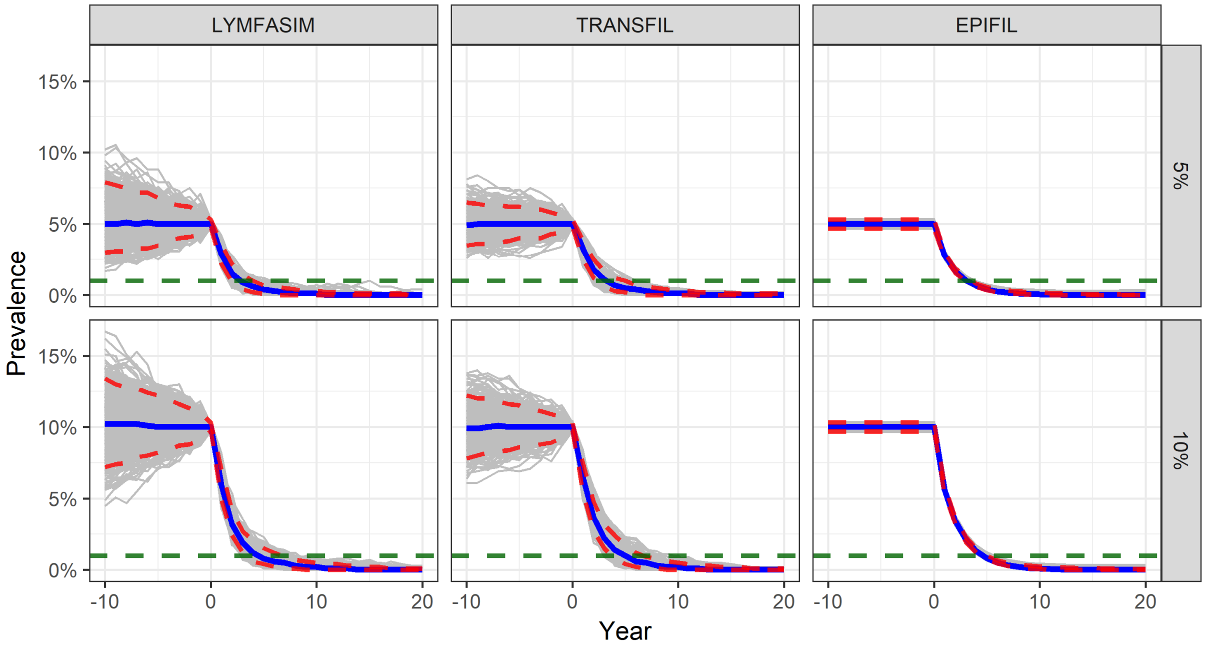

Figure 1.4. Model predicted trends in mf prevalence over time for the reference scenario. Future effort is as currently recommended: aMDA DA 65. In thin grey lines the 501 runs closest to the prevalence shown on the right. Thick blue line: median of these runs. Red dashed lines: 5th and 95th percentile of the runs respectively. Lower green line: the 1% prevalence level goal.

2. INDIA, RECENT START OF MDA

Region: India. Parasite species: *Wuchereria bancrofti*. Vector: *Culex quinquefasciatus*. History of control: 2 years of MDA with DEC+ALB at 65% coverage.

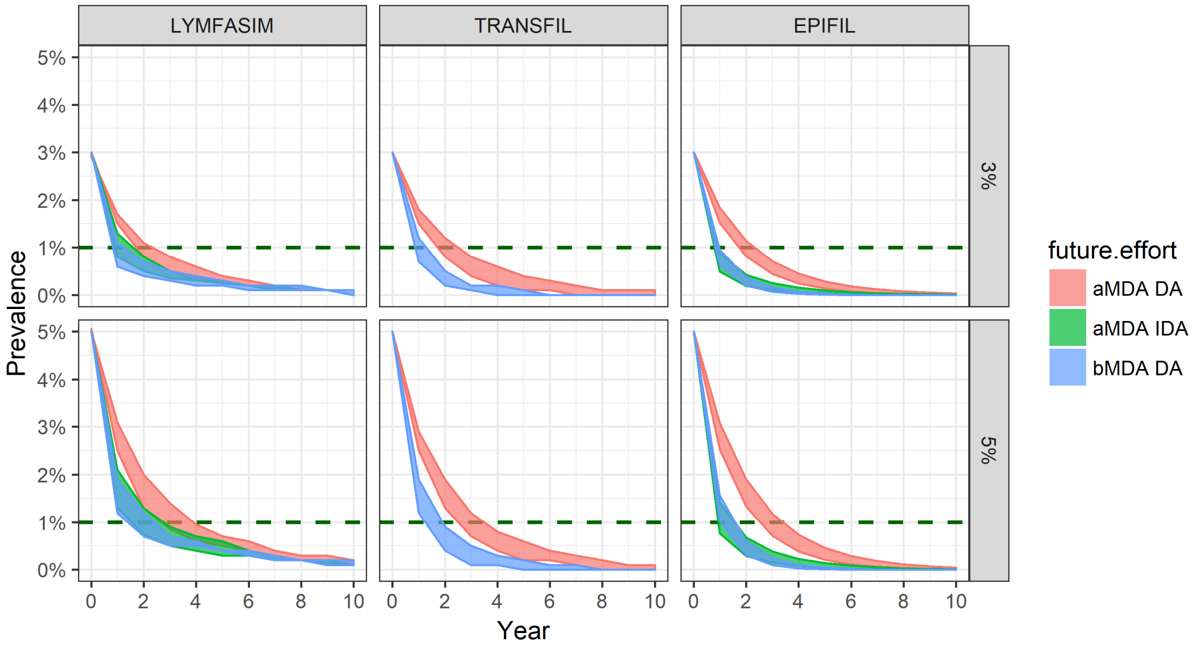

Figure 2.1. Model predicted trends in mf prevalence over time. The median mf prevalence over time is shown over the 501 runs closest to the mf prevalence given on the right. Coloured bands span the 65 to 80% intervention coverage range.

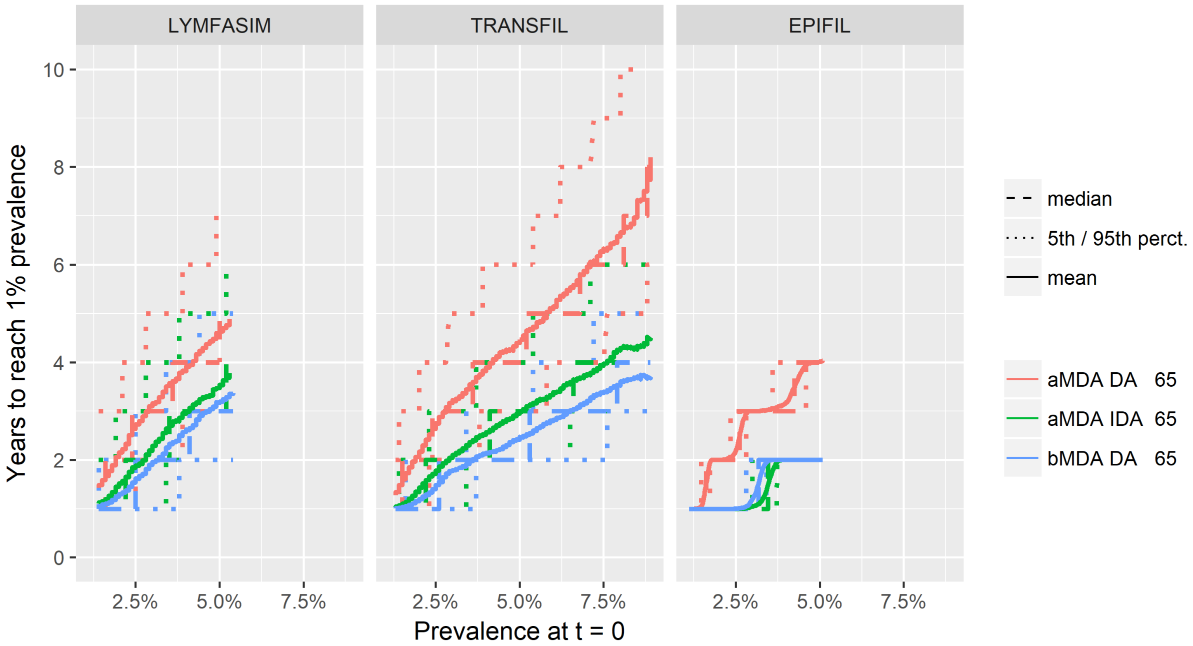

Figure 2.2. Predicted time to reach 1% mf prevalence. Shown are the moving median, the moving 5th and 95th percentile, and the moving average number of intervention years needed to bring mf prevalence below

1% (moving window size = 501. To enable calculation of the average any runs that did not achieve the 1% prevalence threshold by t=20 were set to achieving it by t=21).

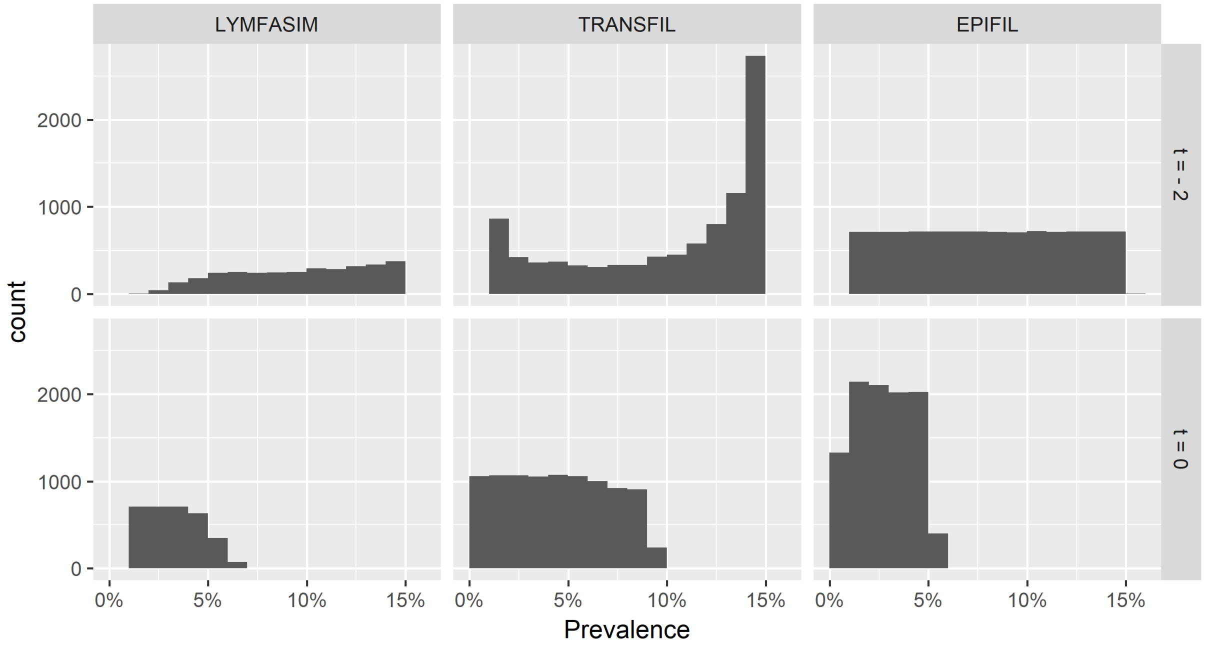

Figure 2.3. Distribution of the mf prevalence. Prevalence shown prior to any control (depending on the past intervention setting this is at t = -10, t = -2 or t = 0), and (if different) mf prevalence at t = 0. Binwidth = 1%.

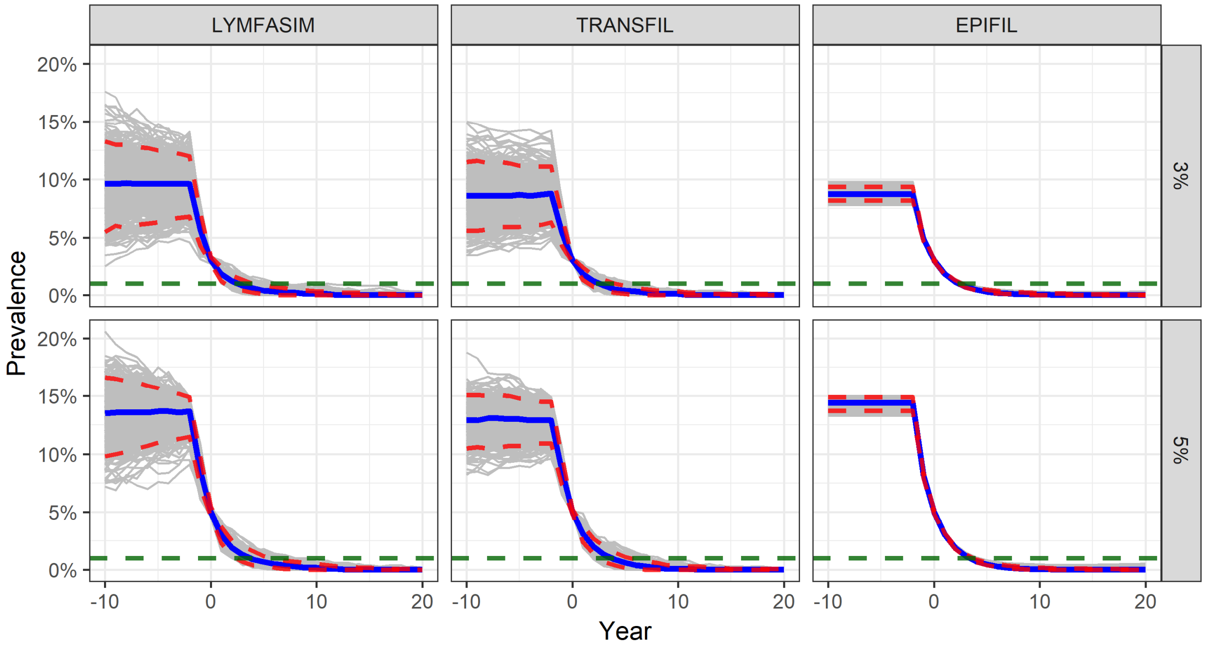

Figure 2.4. Model predicted trends in mf prevalence over time for the reference scenario. Future effort is as currently recommended: aMDA DA 65. In thin grey lines the 501 runs closest to the prevalence shown on the right. Thick blue line: median of these runs. Red dashed lines: 5th and 95th percentile of the runs respectively. Lower green line: the 1% prevalence level goal.

3. INDIA, FAILURE SCENARIO 1

Region: India. Parasite species: *Wuchereria bancrofti*. Vector: *Culex quinquefasciatus*. History of control: Programme failure after 10 years of MDA with 50% coverage.

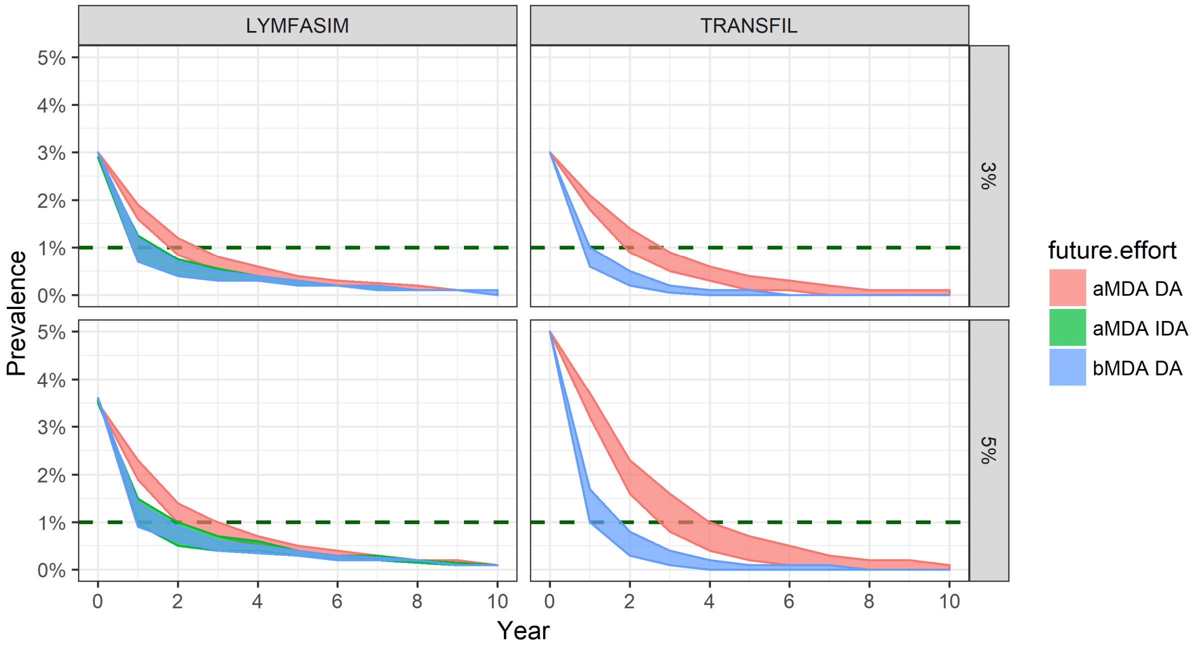

Figure 3.1. Model predicted trends in mf prevalence over time. The median mf prevalence over time is shown over the 501 runs closest to the mf prevalence given on the right. Coloured bands span the 65 to 80% intervention coverage range.

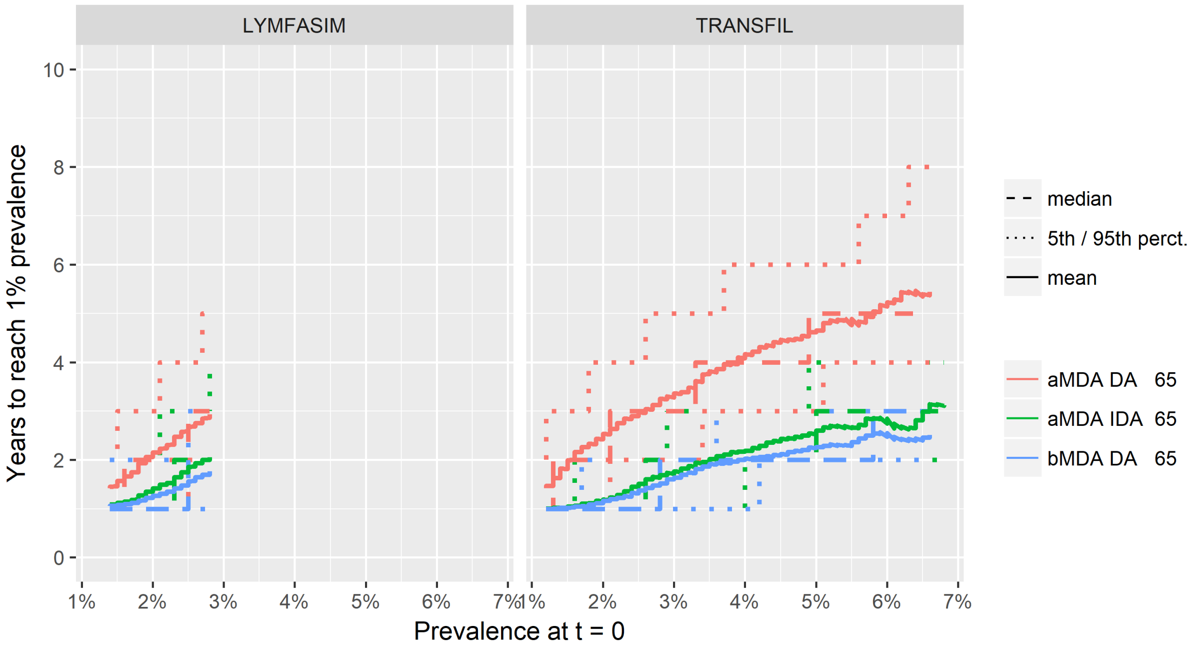

Figure 3.2. Predicted time to reach 1% mf prevalence. Shown are the moving median, the moving 5th and 95th percentile, and the moving average number of intervention years needed to bring mf prevalence below

1% (moving window size = 501. To enable calculation of the average any runs that did not achieve the 1% prevalence threshold by t=20 were set to achieving it by t=21).

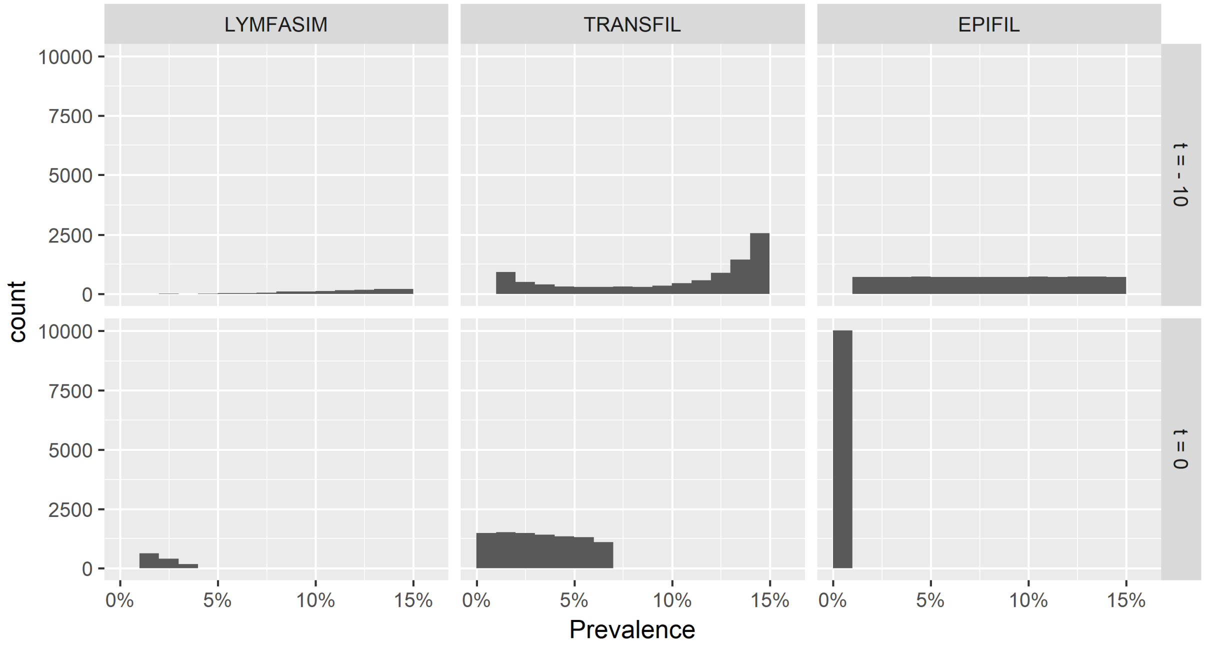

Figure 3.3. Distribution of the mf prevalence. Prevalence shown prior to any control (depending on the past intervention setting this is at t = -10, t = -2 or t = 0), and (if different) mf prevalence at t = 0. Binwidth = 1%.

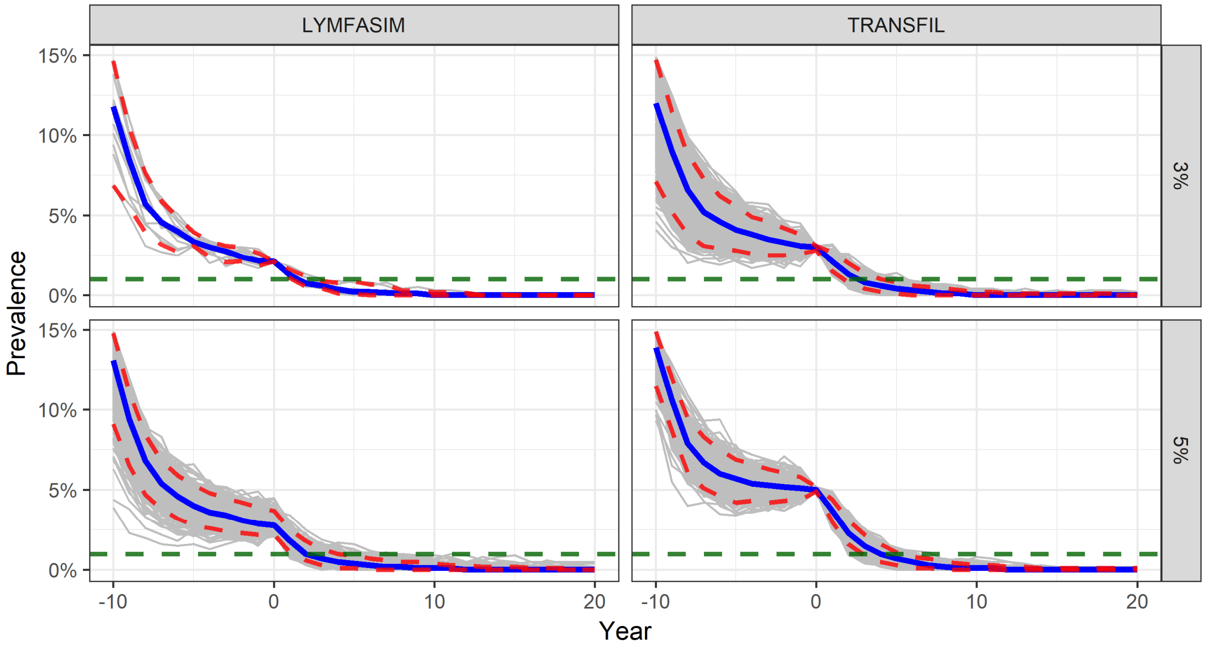

Figure 3.4. Model predicted trends in mf prevalence over time for the reference scenario. Future effort is as currently recommended: aMDA DA 65. In thin grey lines the 501 runs closest to the prevalence shown on the right. Thick blue line: median of these runs. Red dashed lines: 5th and 95th percentile of the runs respectively. Lower green line: the 1% prevalence level goal.

4. INDIA, FAILURE SCENARIO 2

Region: India. Parasite species: *Wuchereria bancrofti*. Vector: *Culex quinquefasciatus*. History of control: Programme failure after 10 years of MDA with 30% coverage.

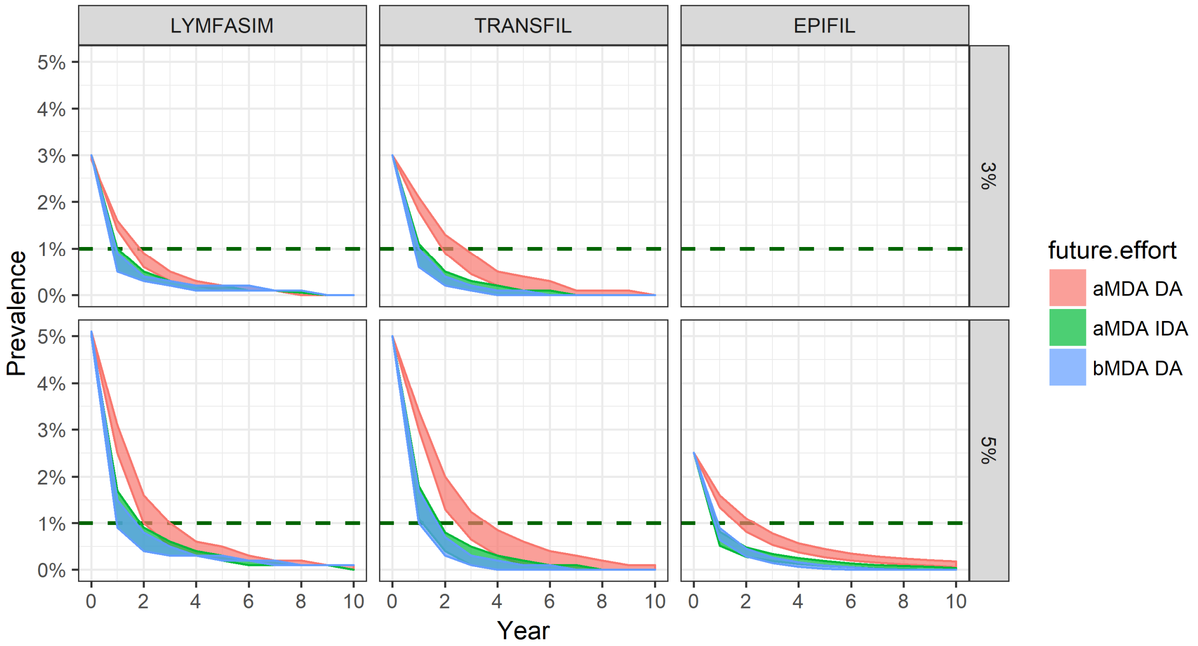

Figure 4.1. Model predicted trends in mf prevalence over time. The median mf prevalence over time is shown over the 501 runs closest to the mf prevalence given on the right. Coloured bands span the 65 to 80% intervention coverage range.

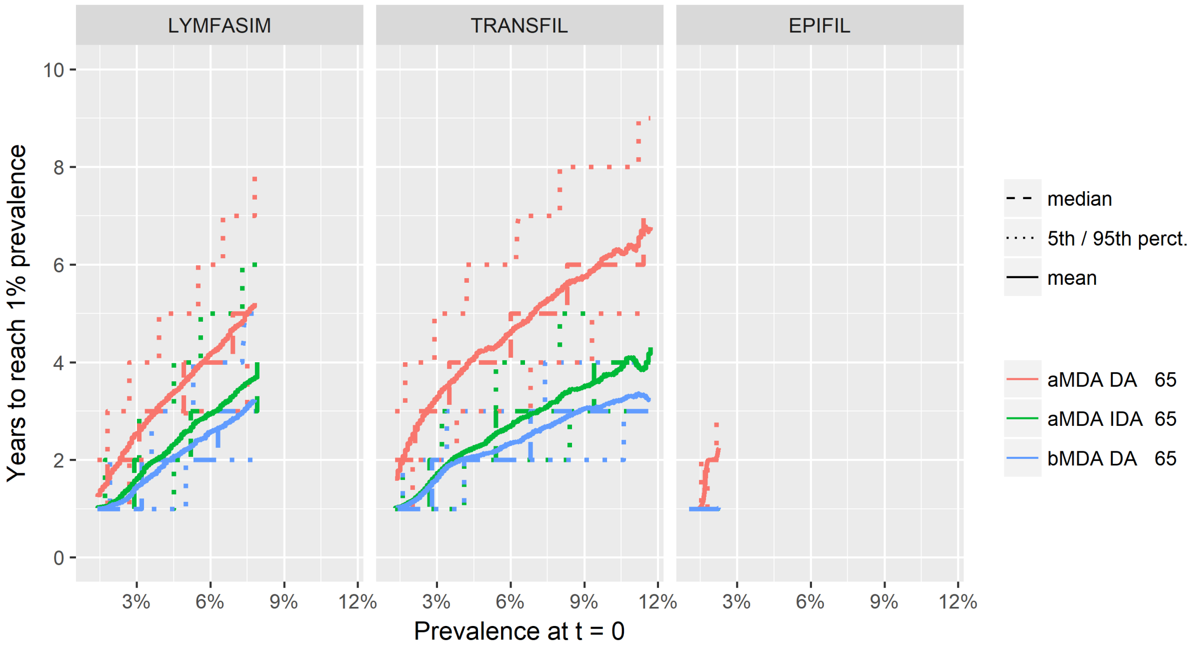

Figure 4.2. Predicted time to reach 1% mf prevalence. Shown are the moving median, the moving 5th and 95th percentile, and the moving average number of intervention years needed to bring mf prevalence below

1% (moving window size = 501. To enable calculation of the average any runs that did not achieve the 1% prevalence threshold by t=20 were set to achieving it by t=21).

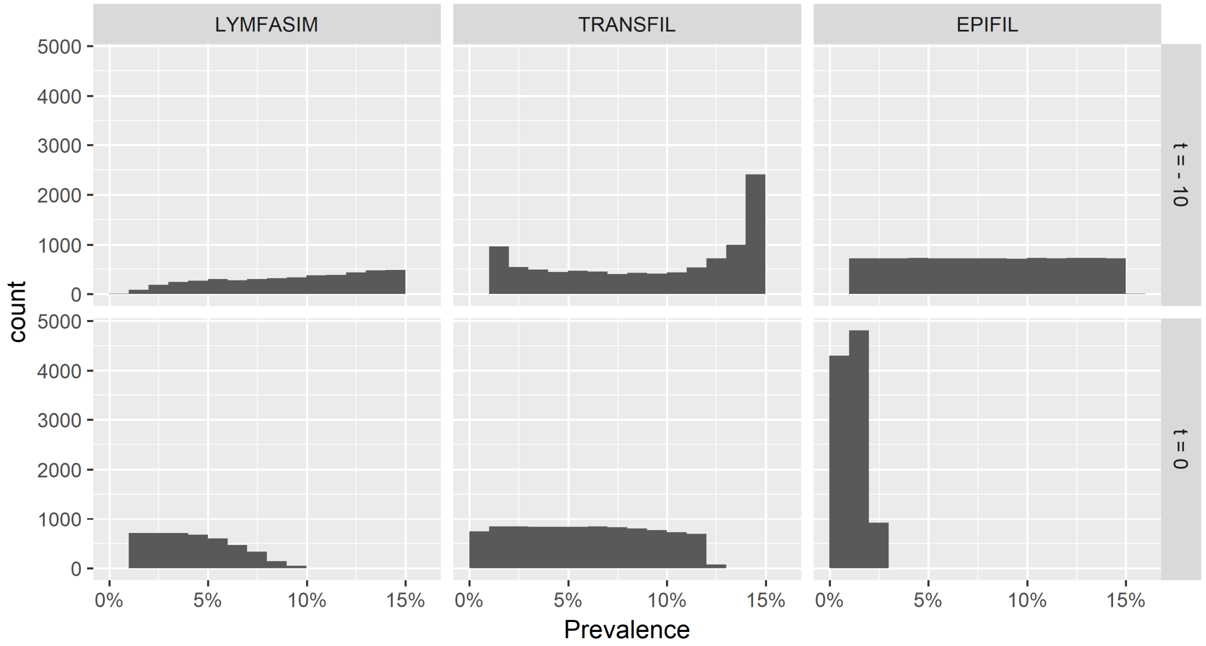

Figure 4.3. Distribution of the mf prevalence. Prevalence shown prior to any control (depending on the past intervention setting this is at t = -10, t = -2 or t = 0), and (if different) mf prevalence at t = 0. Binwidth = 1%.

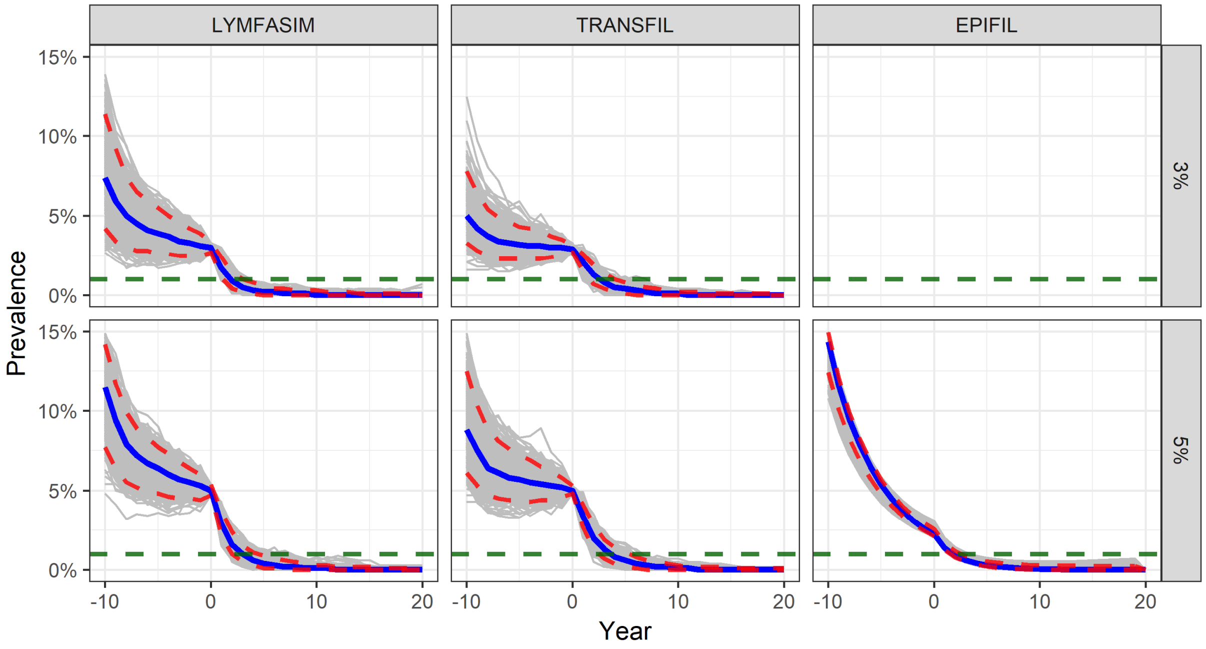

Figure 4.4. Model predicted trends in mf prevalence over time for the reference scenario. Future effort is as currently recommended: aMDA DA 65. In thin grey lines the 501 runs closest to the prevalence shown on the right. Thick blue line: median of these runs. Red dashed lines: 5th and 95th percentile of the runs respectively. Lower green line: the 1% prevalence level goal.

5. PNG, TREATMENT NAIVE

Region: PNG. Parasite species: *Wuchereria bancrofti*. Vector: *Culex quinquefasciatus*. History of control: no prior MDA.

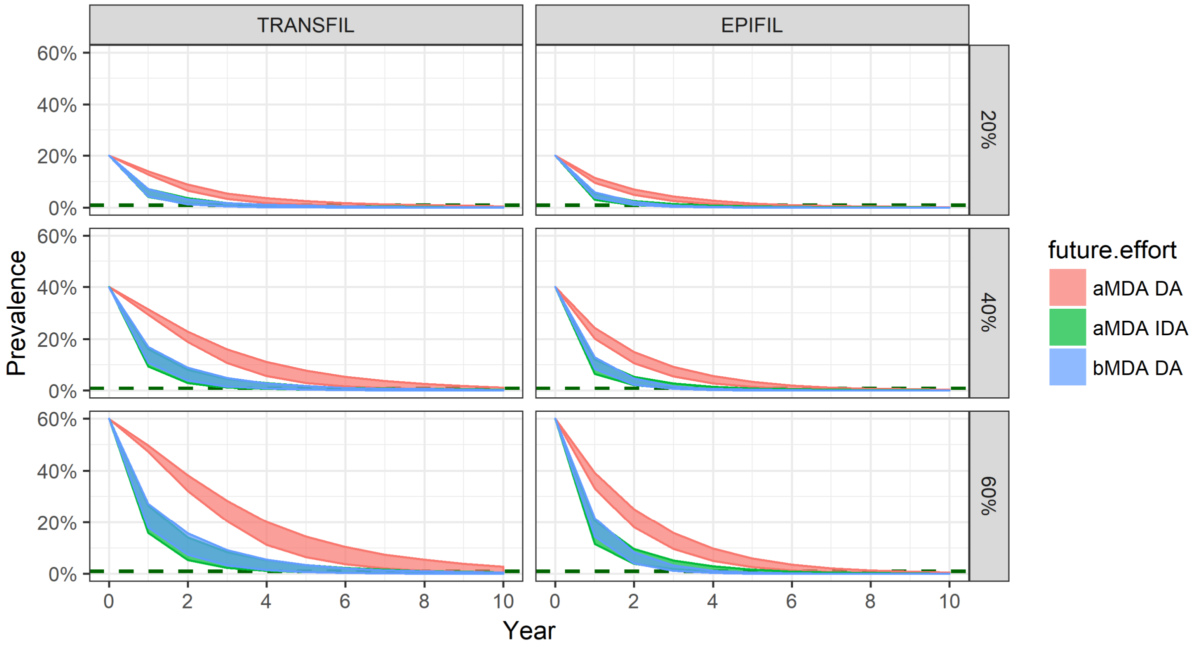

Figure 5.1. Model predicted trends in mf prevalence over time. The median mf prevalence over time is shown over the 501 runs closest to the mf prevalence given on the right. Coloured bands span the 65 to 80% intervention coverage range.

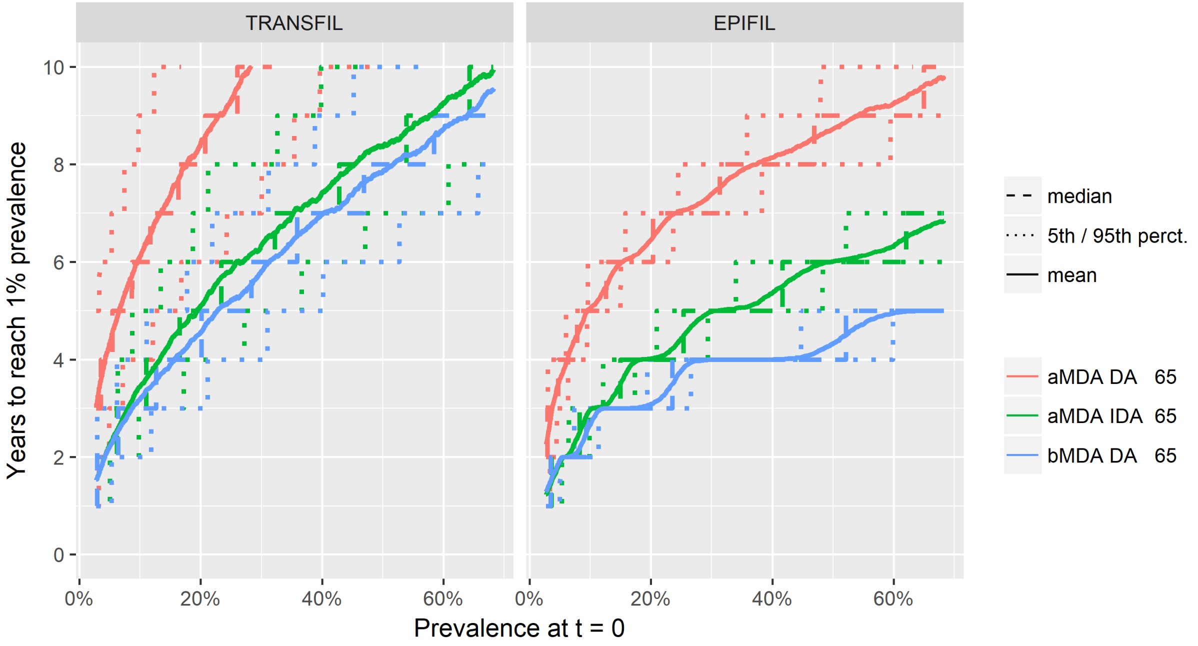

Figure 5.2. Predicted time to reach 1% mf prevalence. Shown are the moving median, the moving 5th and 95th percentile, and the moving average number of intervention years needed to bring mf prevalence below

1% (moving window size = 501. To enable calculation of the average any runs that did not achieve the 1% prevalence threshold by t=20 were set to achieving it by t=21).

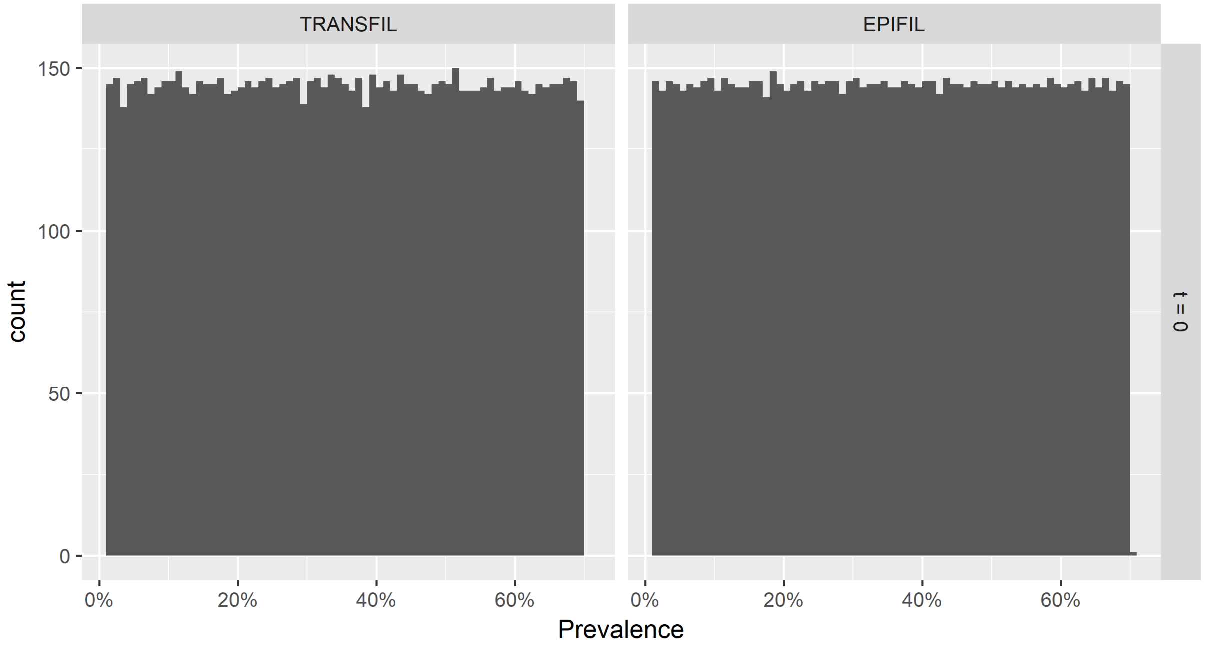

Figure 5.3. Distribution of the mf prevalence. Prevalence shown prior to any control (depending on the past intervention setting this is at t = -10, t = -2 or t = 0), and (if different) mf prevalence at t = 0. Binwidth = 1%.

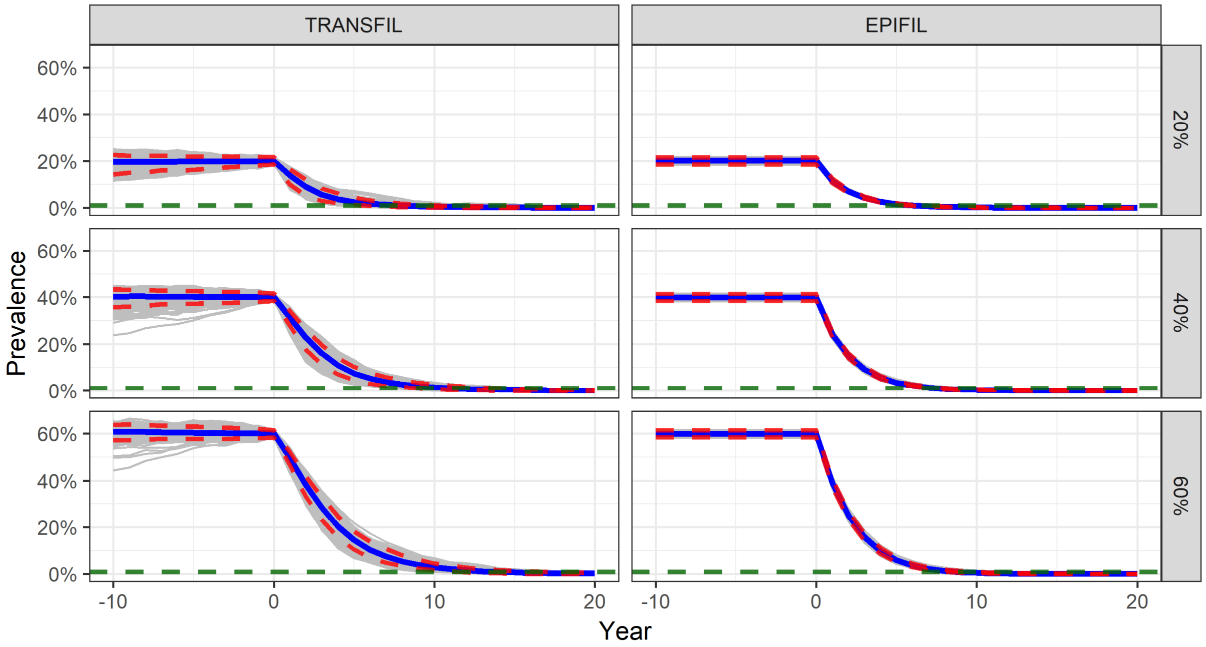

Figure 5.4. Model predicted trends in mf prevalence over time for the reference scenario. Future effort is as currently recommended: aMDA DA 65. In thin grey lines the 501 runs closest to the prevalence shown on the right. Thick blue line: median of these runs. Red dashed lines: 5th and 95th percentile of the runs respectively. Lower green line: the 1% prevalence level goal.

6. PNG, RECENT START OF MDA

Region: PNG. Parasite species: *Wuchereria bancrofti*. Vector: *Culex quinquefasciatus*. History of control: 2 years of MDA with DEC+ALB at 65% coverage.

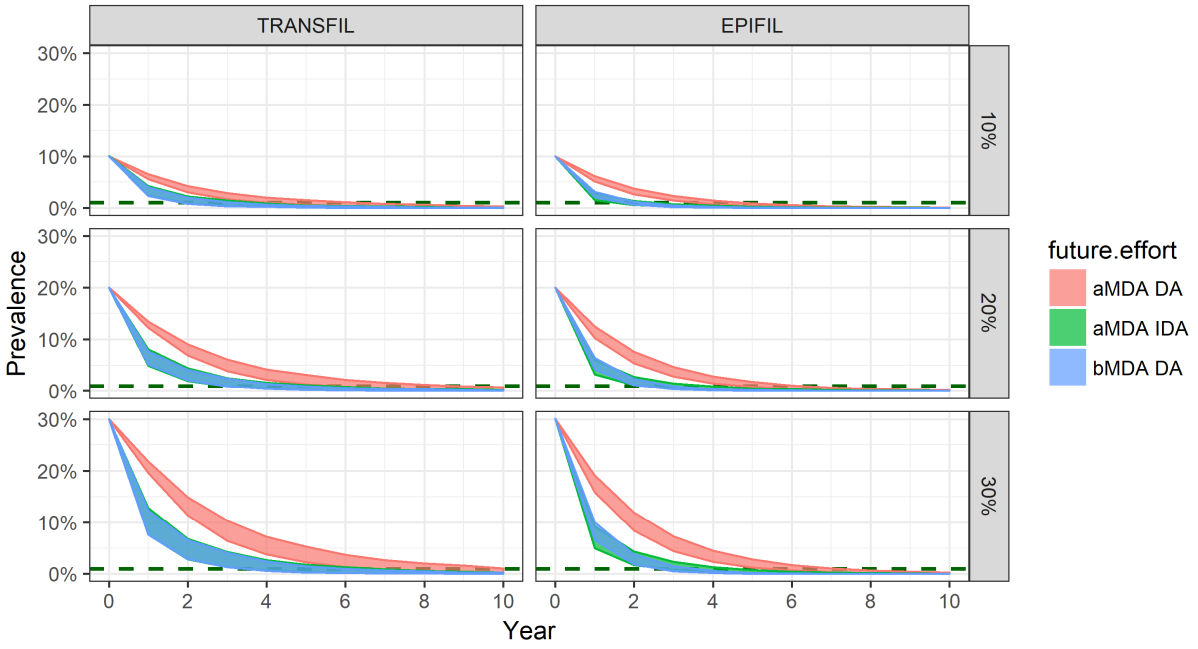

Figure 6.1. Model predicted trends in mf prevalence over time. The median mf prevalence over time is shown over the 501 runs closest to the mf prevalence given on the right. Coloured bands span the 65 to 80% intervention coverage range.

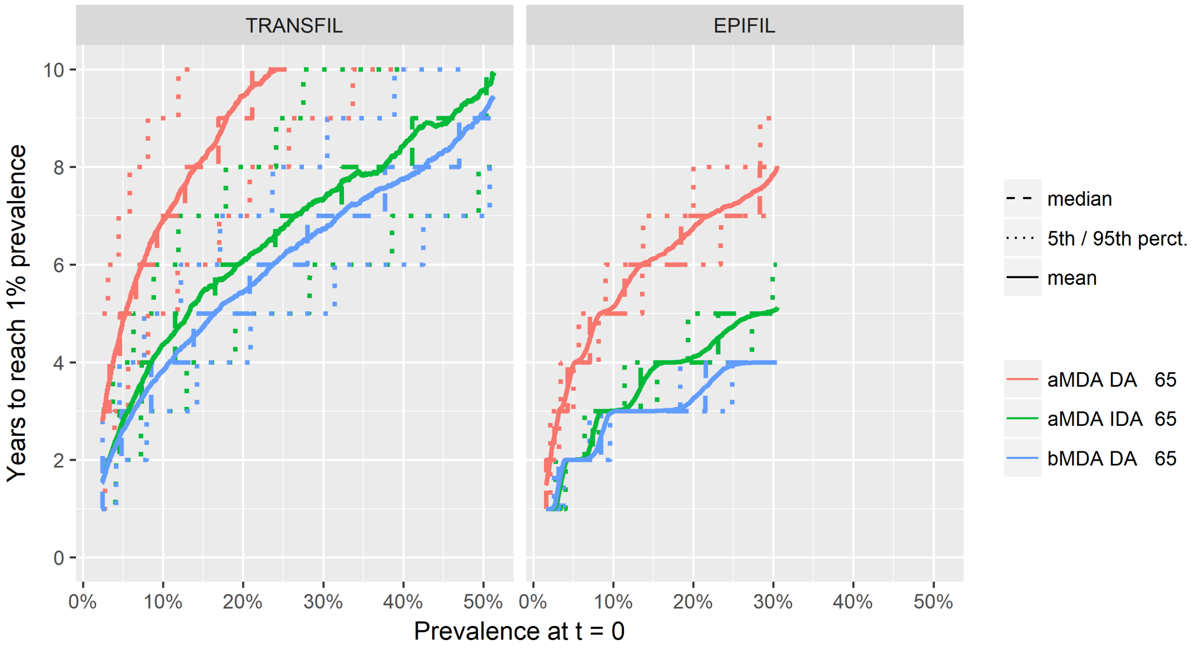

Figure 6.2. Predicted time to reach 1% mf prevalence. Shown are the moving median, the moving 5th and 95th percentile, and the moving average number of intervention years needed to bring mf prevalence below

1% (moving window size = 501. To enable calculation of the average any runs that did not achieve the 1% prevalence threshold by t=20 were set to achieving it by t=21).

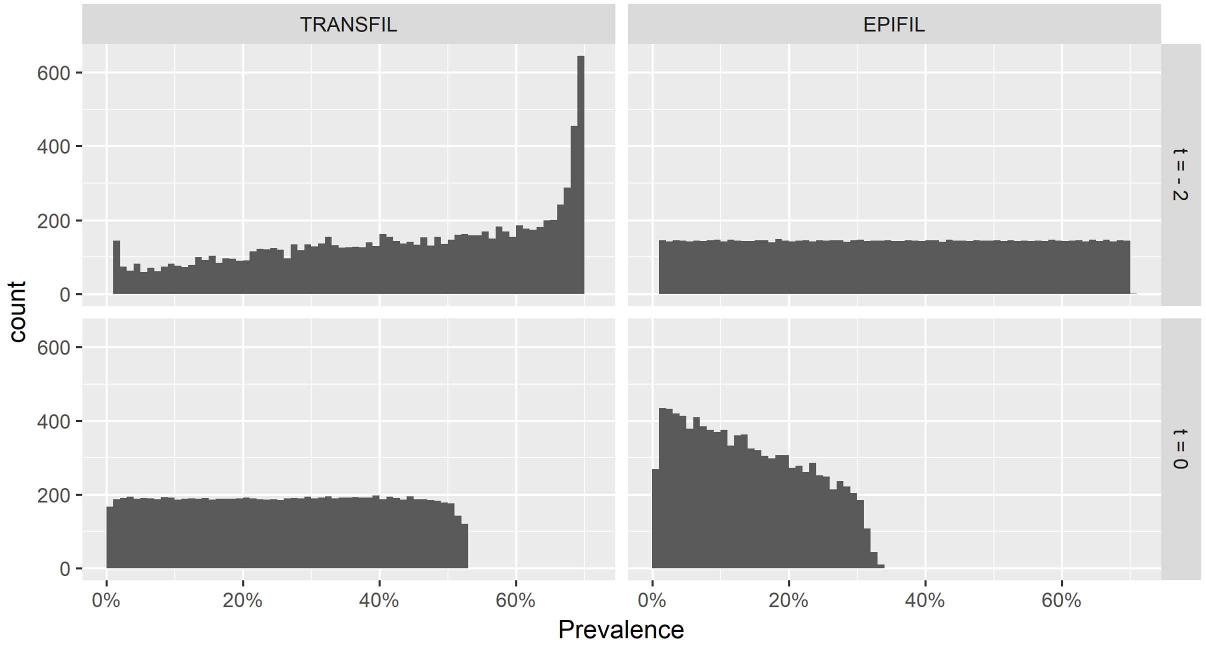

Figure 6.3. Distribution of the mf prevalence. Prevalence shown prior to any control (depending on the past intervention setting this is at *t* = -10, *t* = -2 or *t* = 0), and (if different) mf prevalence at *t* = 0. Binwidth = 1%.

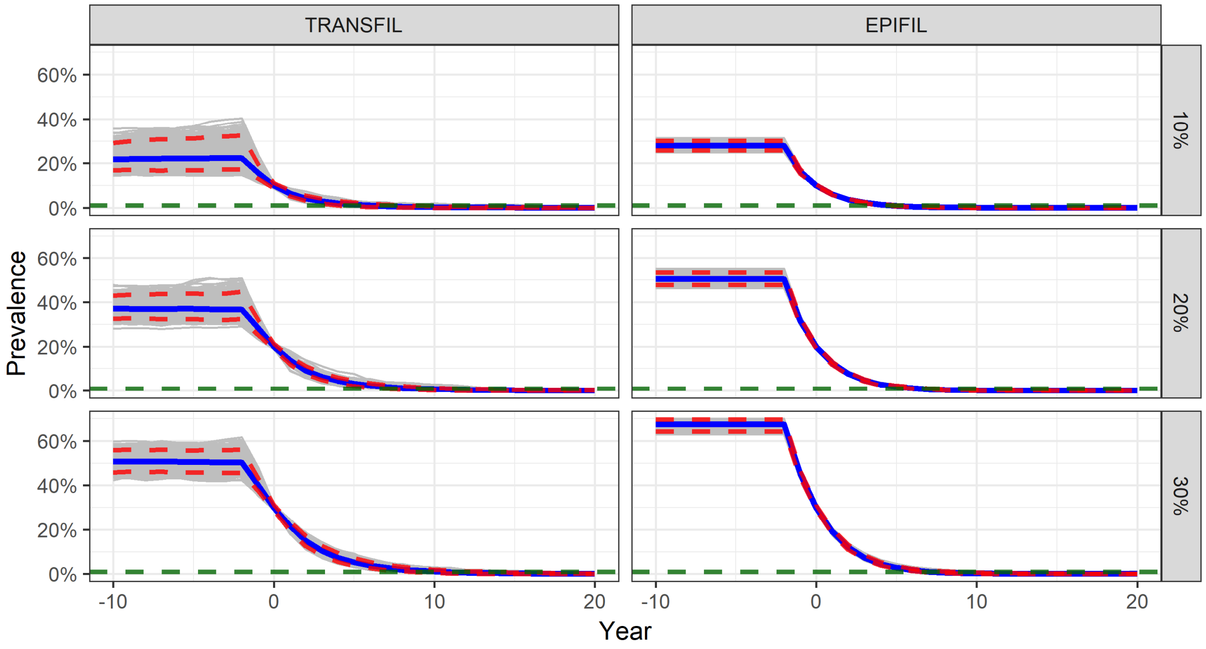

Figure 6.4. Model predicted trends in mf prevalence over time for the reference scenario. Future effort is as currently recommended: aMDA DA 65. In thin grey lines the 501 runs closest to the prevalence shown on the right. Thick blue line: median of these runs. Red dashed lines: 5th and 95th percentile of the runs respectively. Lower green line: the 1% prevalence level goal.

7. PNG, FAILURE SCENARIO 1

Region: PNG. Parasite species: *Wuchereria bancrofti*. Vector: *Culex quinquefasciatus*. History of control: Programme failure after 10 years of MDA with 50% coverage.

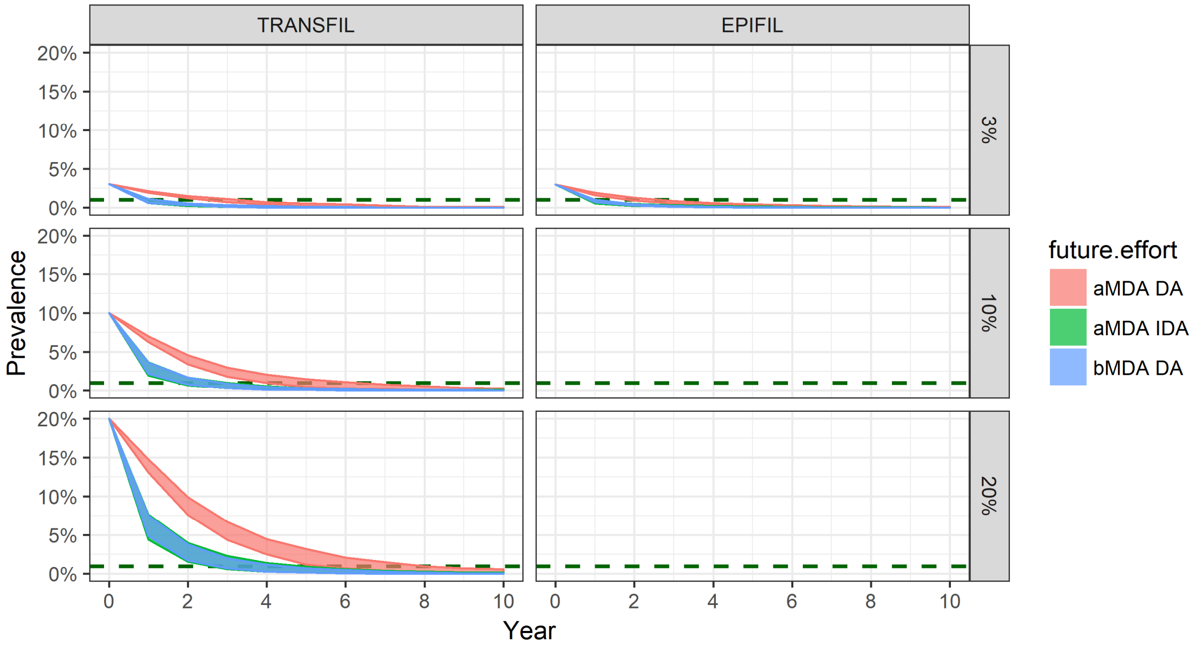

Figure 7.1. Model predicted trends in mf prevalence over time. The median mf prevalence over time is shown over the 501 runs closest to the mf prevalence given on the right. Coloured bands span the 65 to 80% intervention coverage range.

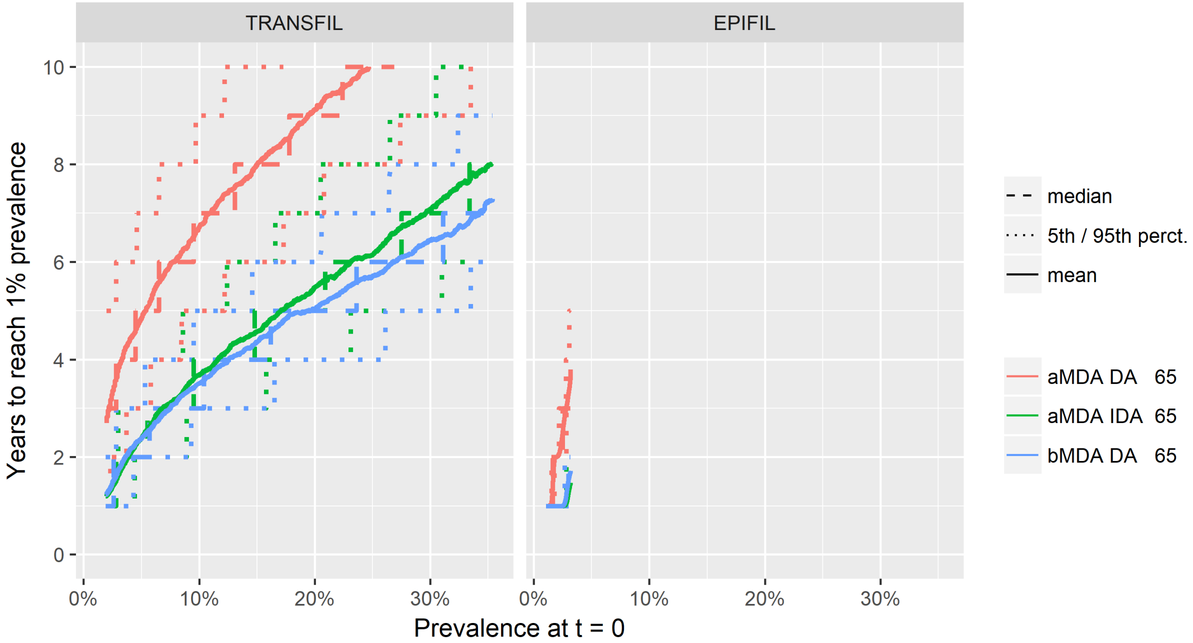

Figure 7.2. Predicted time to reach 1% mf prevalence. Shown are the moving median, the moving 5th and 95th percentile, and the moving average number of intervention years needed to bring mf prevalence below

1% (moving window size = 501. To enable calculation of the average any runs that did not achieve the 1% prevalence threshold by t=20 were set to achieving it by t=21).

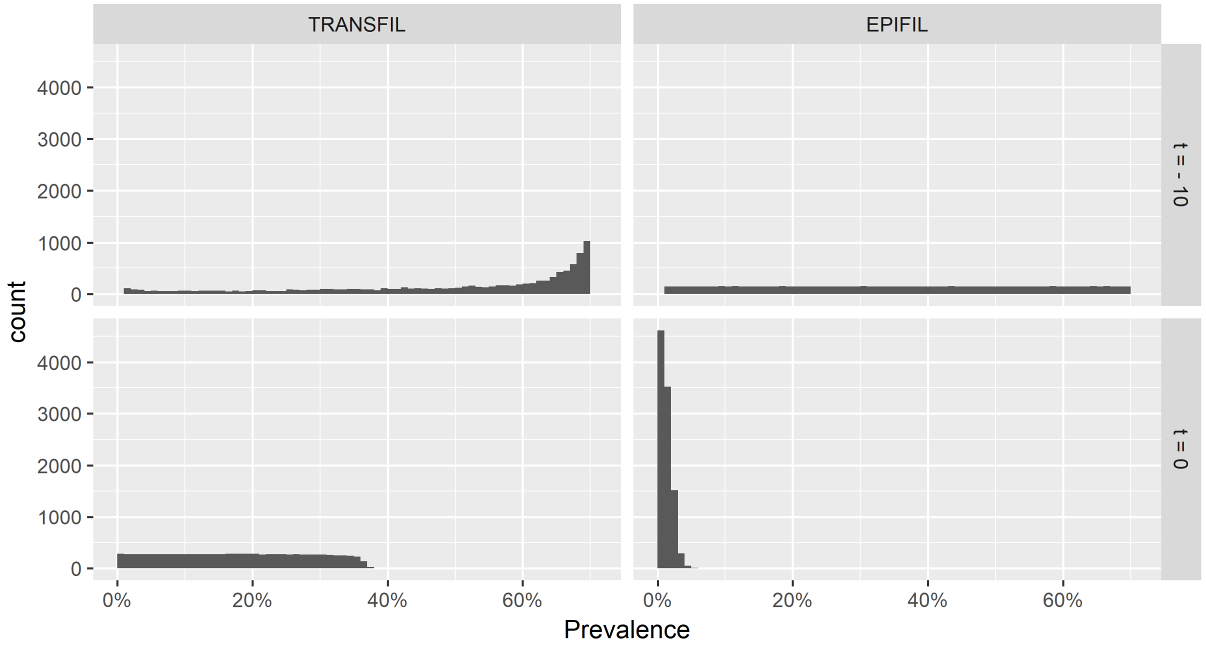

Figure 7.3. Distribution of the mf prevalence. Prevalence shown prior to any control (depending on the past intervention setting this is at t = -10, t = -2 or t = 0), and (if different) mf prevalence at t = 0. Binwidth = 1%.

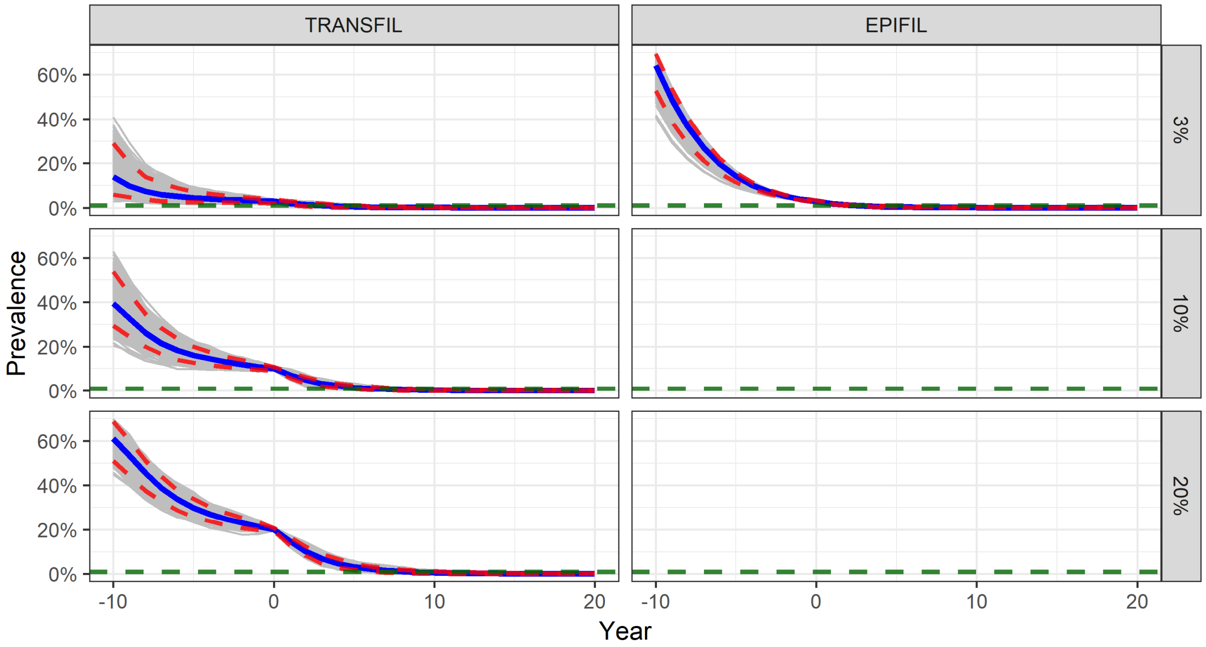

Figure 7.4. Model predicted trends in mf prevalence over time for the reference scenario. Future effort is as currently recommended: aMDA DA 65. In thin grey lines the 501 runs closest to the prevalence shown on the right. Thick blue line: median of these runs. Red dashed lines: 5th and 95th percentile of the runs respectively. Lower green line: the 1% prevalence level goal.

8. PNG, FAILURE SCENARIO 2

Region: PNG. Parasite species: *Wuchereria bancrofti*. Vector: *Culex quinquefasciatus*. History of control: Programme failure after 10 years of MDA with 30% coverage.

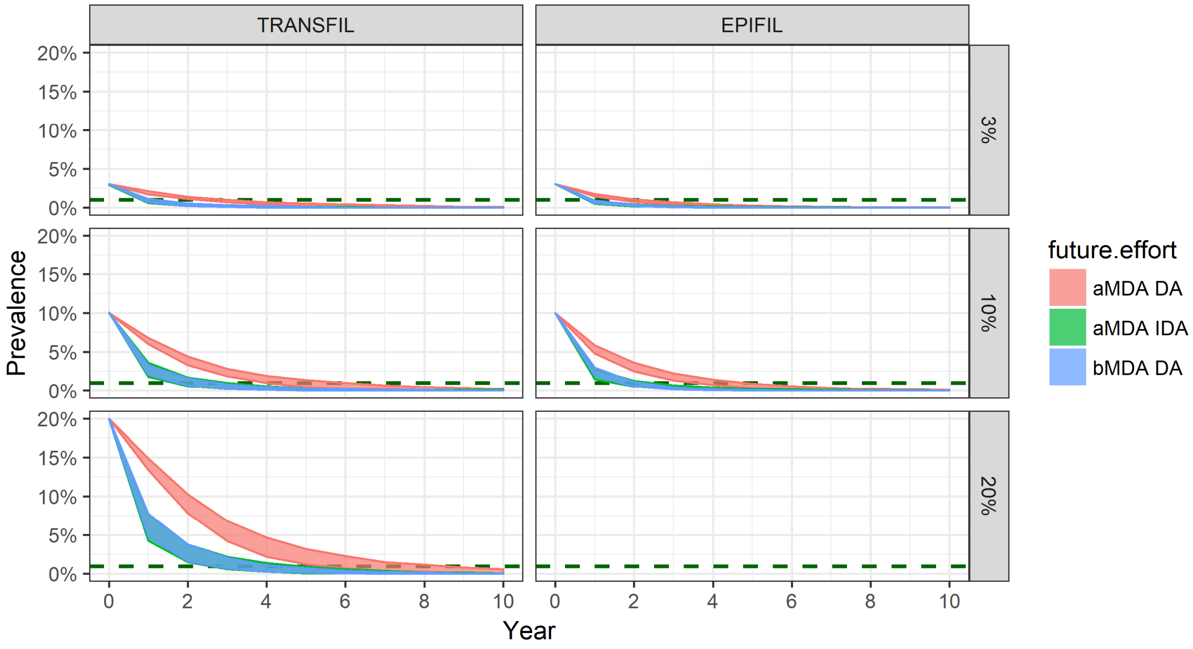

Figure 8.1. Model predicted trends in mf prevalence over time. The median mf prevalence over time is shown over the 501 runs closest to the mf prevalence given on the right. Coloured bands span the 65 to 80% intervention coverage range.

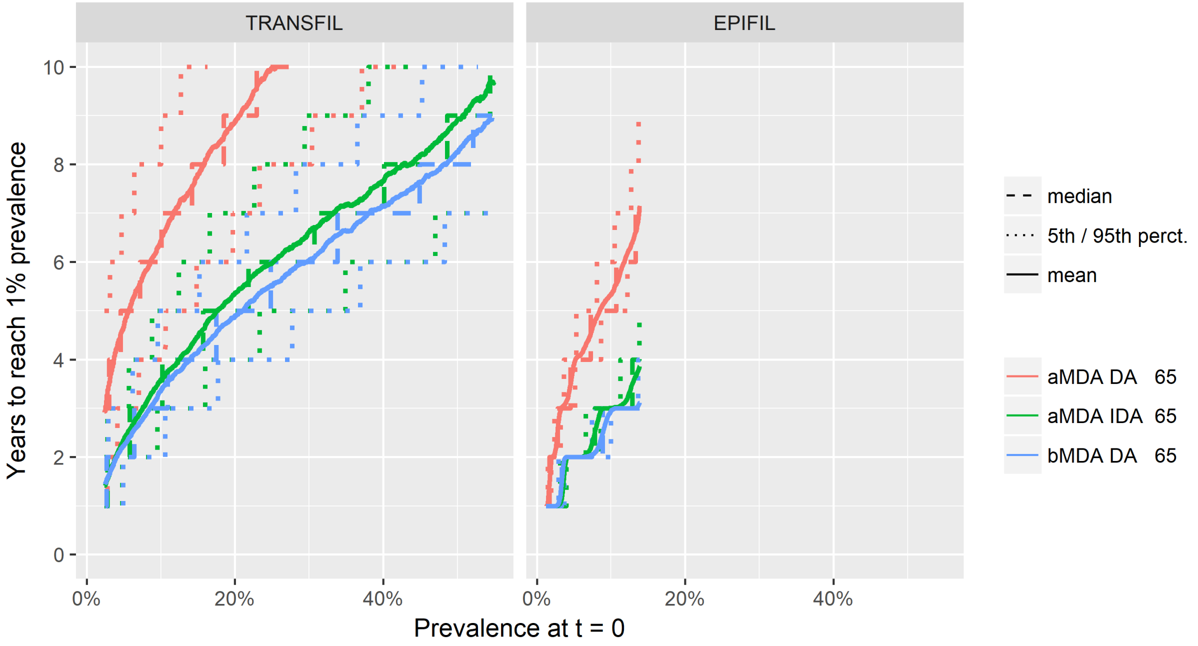

Figure 8.2. Predicted time to reach 1% mf prevalence. Shown are the moving median, the moving 5th and 95th percentile, and the moving average number of intervention years needed to bring mf prevalence below

1% (moving window size = 501. To enable calculation of the average any runs that did not achieve the 1% prevalence threshold by t=20 were set to achieving it by t=21).

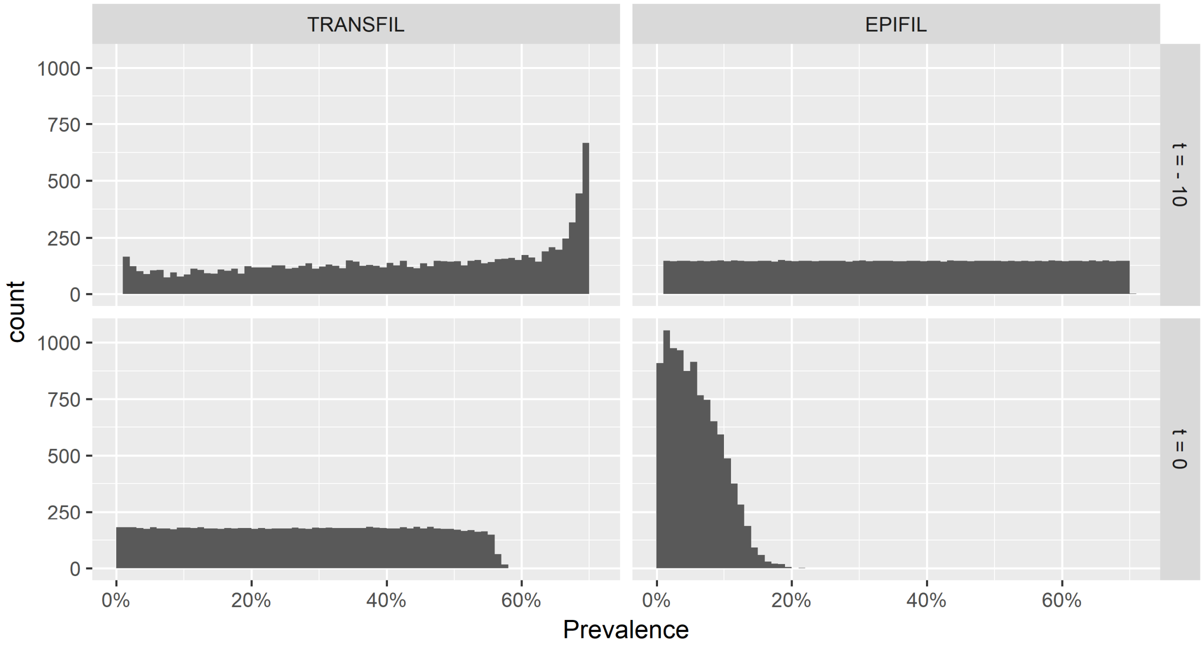

Figure 8.3. Distribution of the mf prevalence. Prevalence shown prior to any control (depending on the past intervention setting this is at t = -10, t = -2 or t = 0), and (if different) mf prevalence at t = 0. Binwidth = 1%.

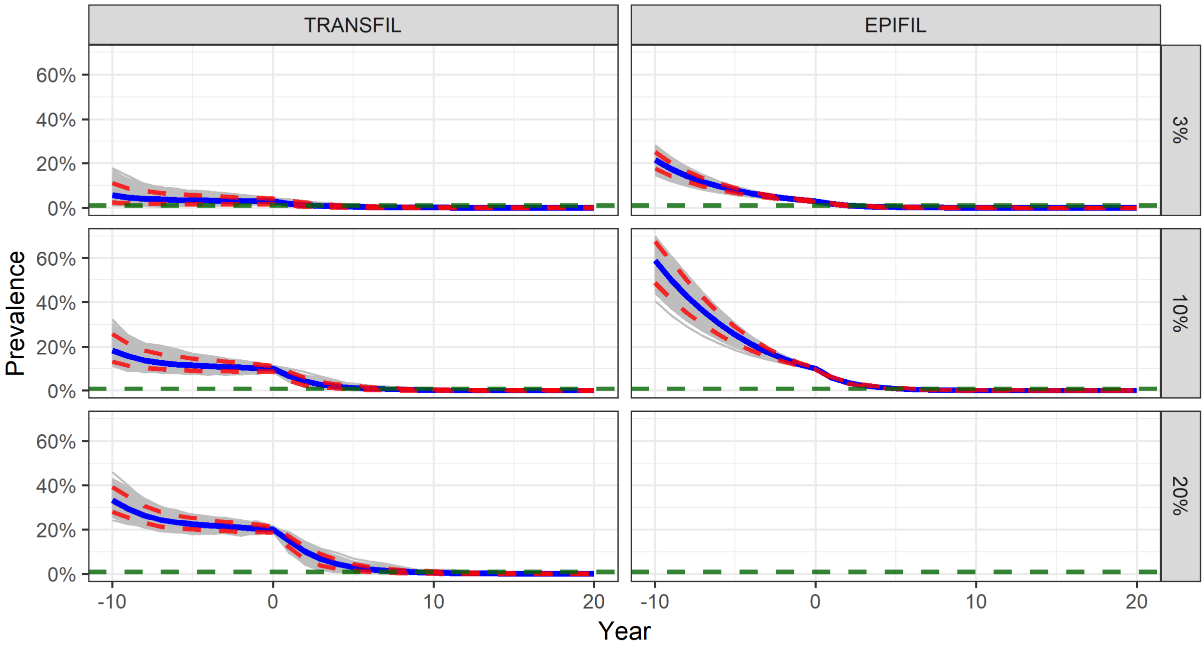

Figure 8.4. Model predicted trends in mf prevalence over time for the reference scenario. Future effort is as currently recommended: aMDA DA 65. In thin grey lines the 501 runs closest to the prevalence shown on the right. Thick blue line: median of these runs. Red dashed lines: 5th and 95th percentile of the runs respectively. Lower green line: the 1% prevalence level goal.

9. AFRICA, DEC+ALB (ONCHOCERCIASIS AND *LOA LOA* FREE), TREATMENT NAIVE

Region: Africa. Parasite species: *Wuchereria bancrofti*. Vector: *Anopheles species*. History of control: no prior MDA.

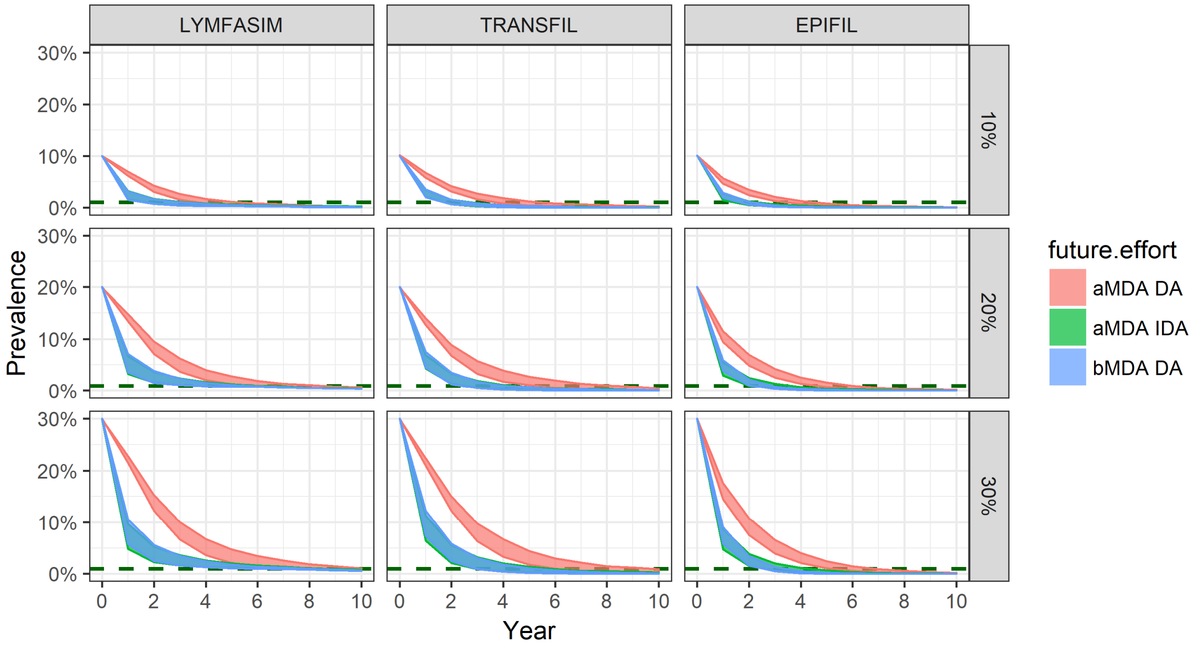

Figure 9.1. Model predicted trends in mf prevalence over time. The median mf prevalence over time is shown over the 501 runs closest to the mf prevalence given on the right. Coloured bands span the 65 to 80% intervention coverage range.

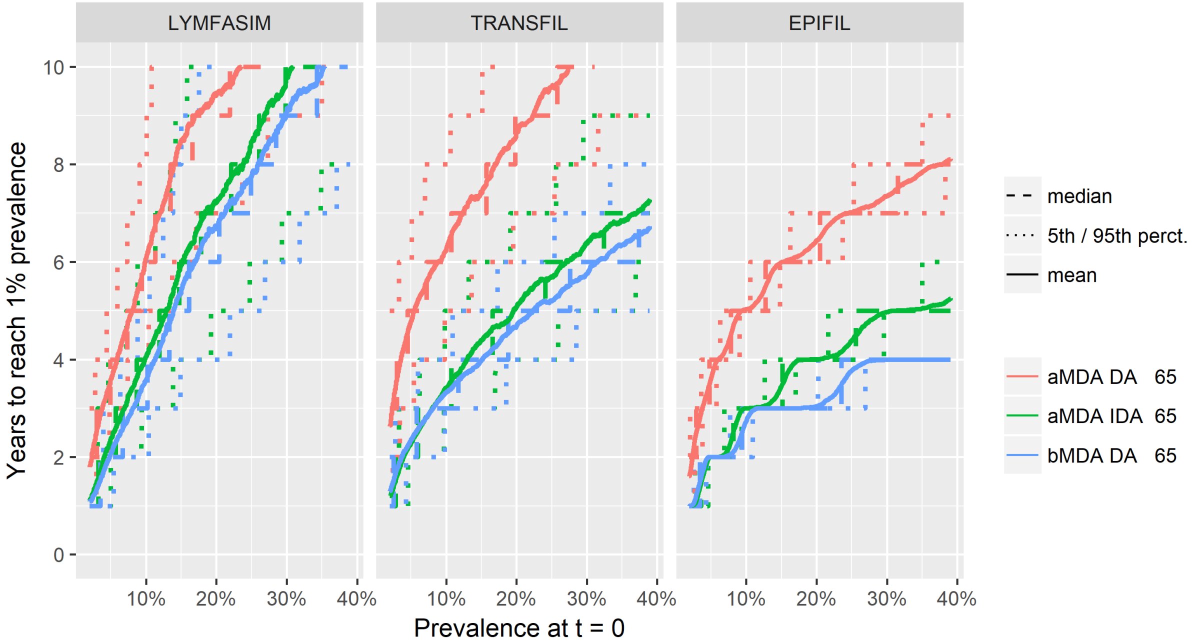

Figure 9.2. Predicted time to reach 1% mf prevalence. Shown are the moving median, the moving 5th and 95th percentile, and the moving average number of intervention years needed to bring mf prevalence below

1% (moving window size = 501. To enable calculation of the average any runs that did not achieve the 1% prevalence threshold by t=20 were set to achieving it by t=21).

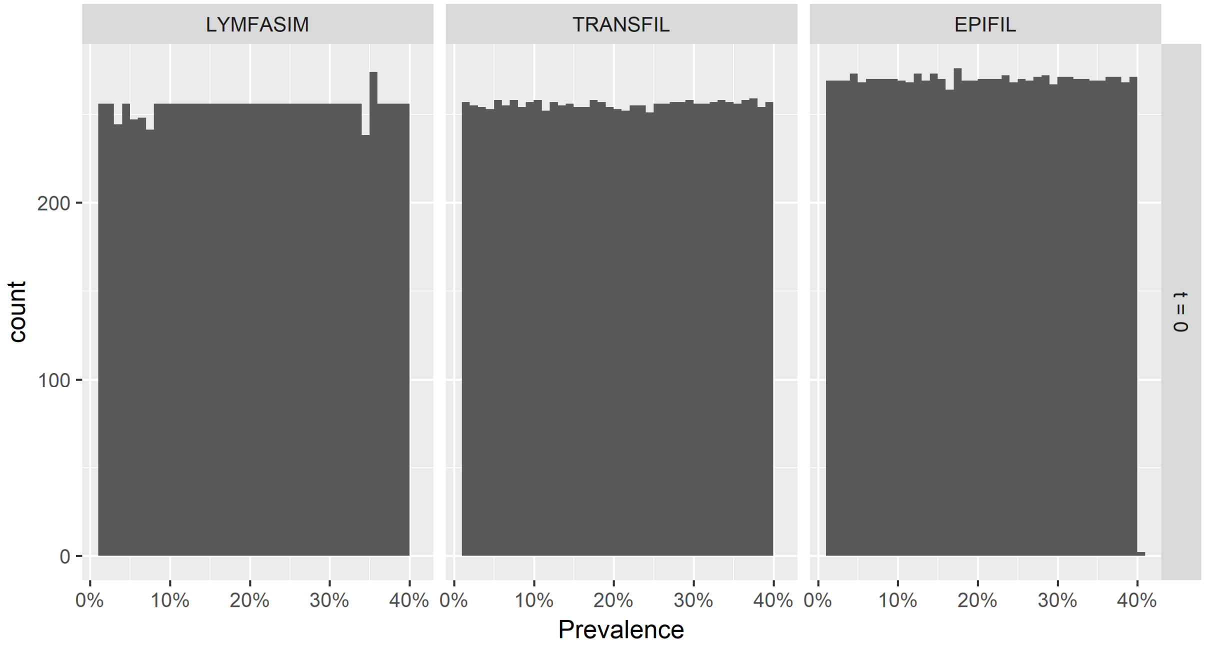

Figure 9.3. Distribution of the mf prevalence. Prevalence shown prior to any control (depending on the past intervention setting this is at t = -10, t = -2 or t = 0), and (if different) mf prevalence at t = 0. Binwidth = 1%.

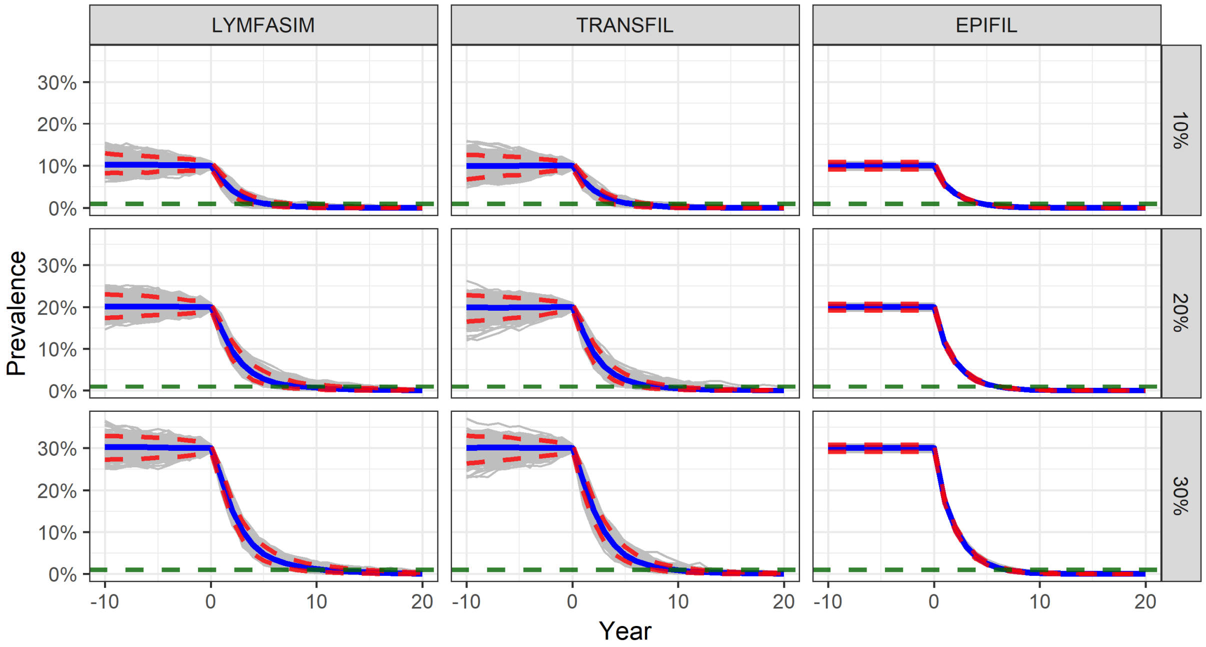

Figure 9.4. Model predicted trends in mf prevalence over time for the reference scenario. Future effort is as currently recommended: aMDA DA 65. In thin grey lines the 501 runs closest to the prevalence shown on the right. Thick blue line: median of these runs. Red dashed lines: 5th and 95th percentile of the runs respectively. Lower green line: the 1% prevalence level goal.

10. AFRICA, DEC+ALB (ONCHOCERCIASIS AND LOA LOA FREE), RECENT START OF MDA

Region: Africa. Parasite species: *Wuchereria bancrofti*. Vector: *Anopheles species*. History of control: 2 years of MDA with DEC+ALB at 65% coverage.

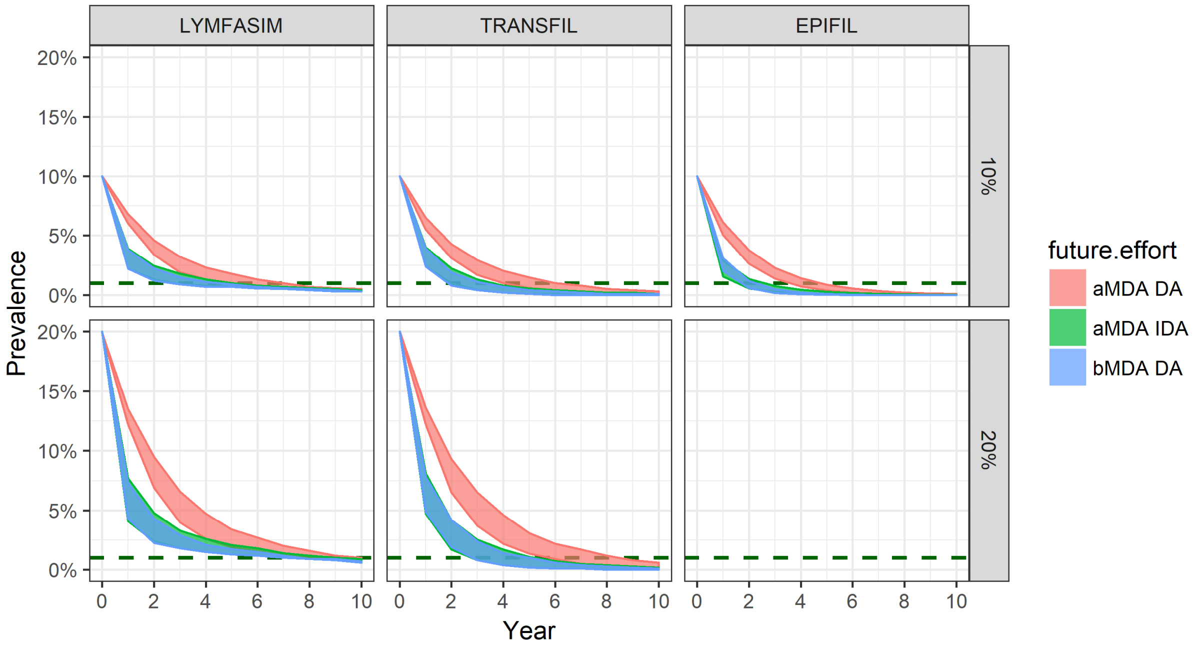

Figure 10.1. Model predicted trends in mf prevalence over time. The median mf prevalence over time is shown over the 501 runs closest to the mf prevalence given on the right. Coloured bands span the 65 to 80% intervention coverage range.

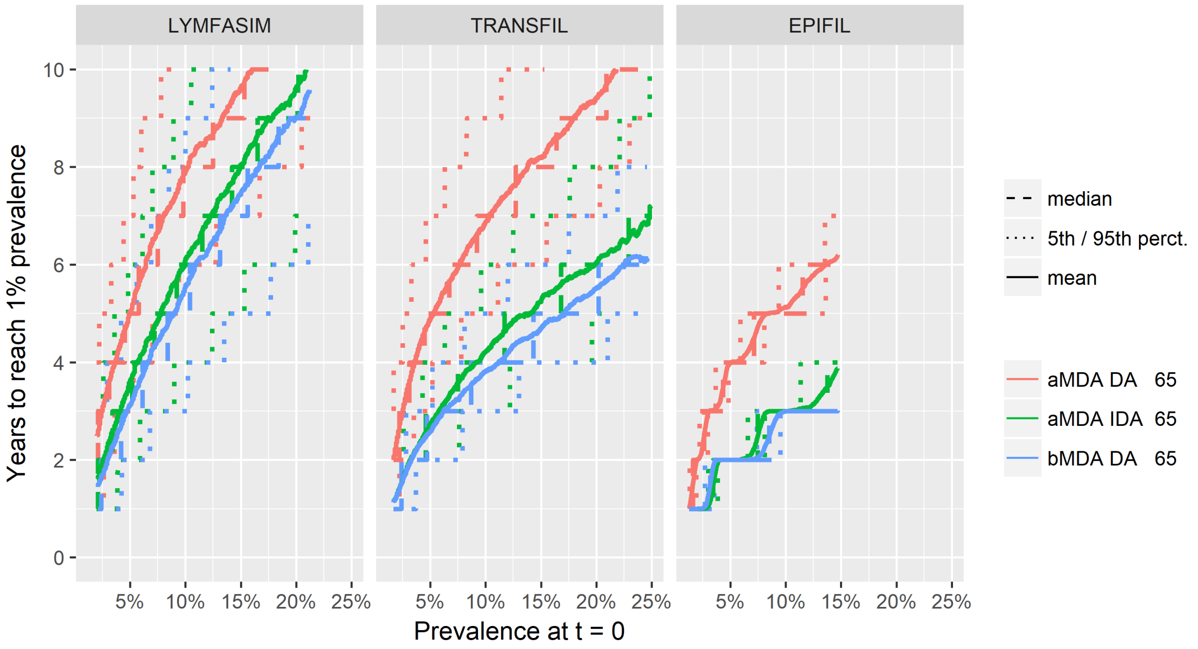

Figure 10.2. Predicted time to reach 1% mf prevalence. Shown are the moving median, the moving 5th and 95th percentile, and the moving average number of intervention years needed to bring mf prevalence below

1% (moving window size = 501. To enable calculation of the average any runs that did not achieve the 1% prevalence threshold by t=20 were set to achieving it by t=21).

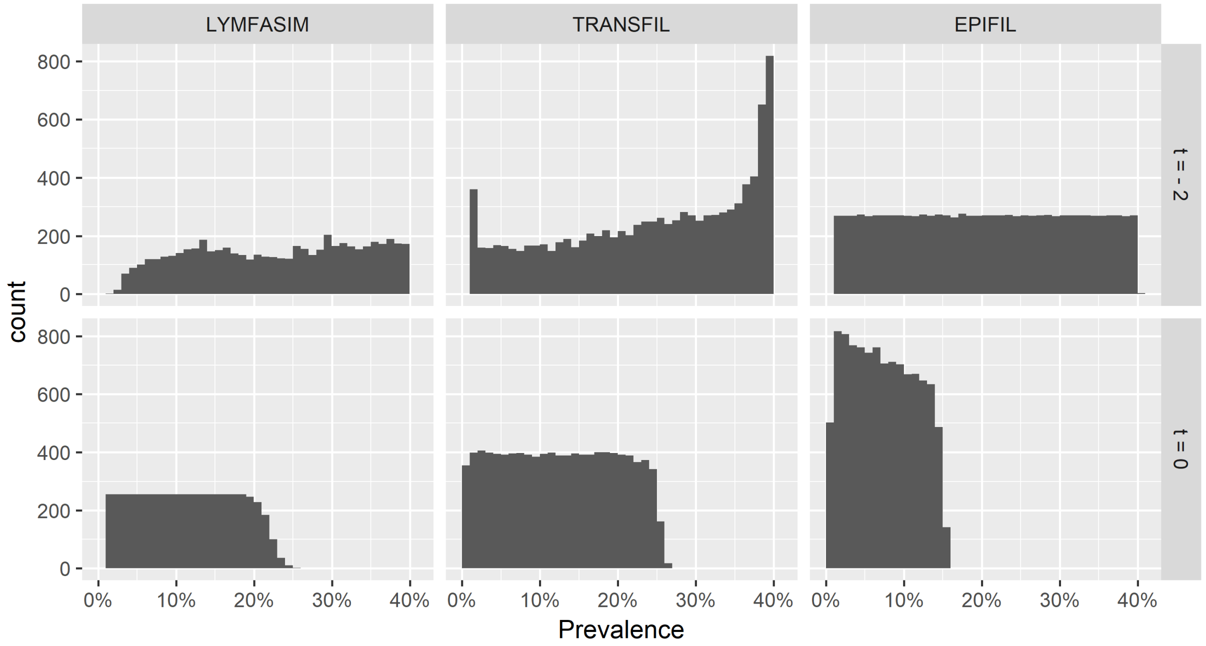

Figure 10.3. Distribution of the mf prevalence. Prevalence shown prior to any control (depending on the past intervention setting this is at *t* = -10, *t* = -2 or *t* = 0), and (if different) mf prevalence at *t* = 0. Binwidth = 1%.

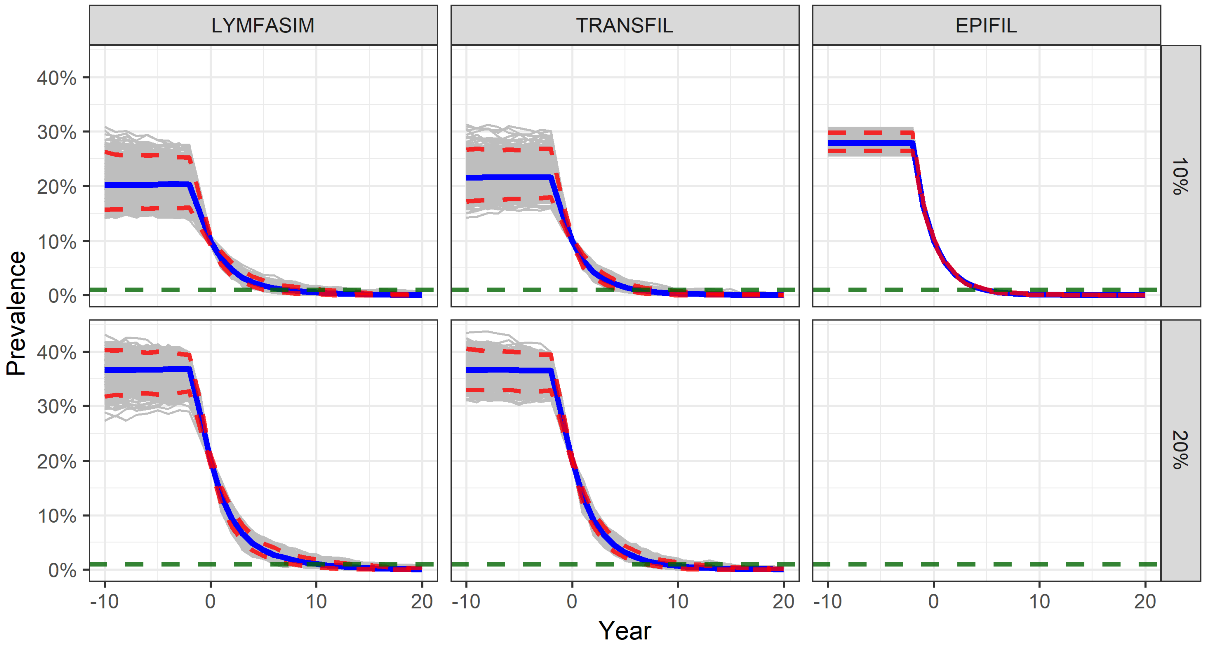

Figure 10.4. Model predicted trends in mf prevalence over time for the reference scenario. Future effort is as currently recommended: aMDA DA 65. In thin grey lines the 501 runs closest to the prevalence shown on the right. Thick blue line: median of these runs. Red dashed lines: 5th and 95th percentile of the runs respectively. Lower green line: the 1% prevalence level goal.

11. AFRICA, DEC+ALB (ONCHOCERCIASIS AND LOA LOA FREE), FAILURE SCENARIO 1

Region: Africa. Parasite species: *Wuchereria bancrofti*. Vector: *Anopheles species*.
History of control: Programme failure after 10 years of MDA with 50% coverage.

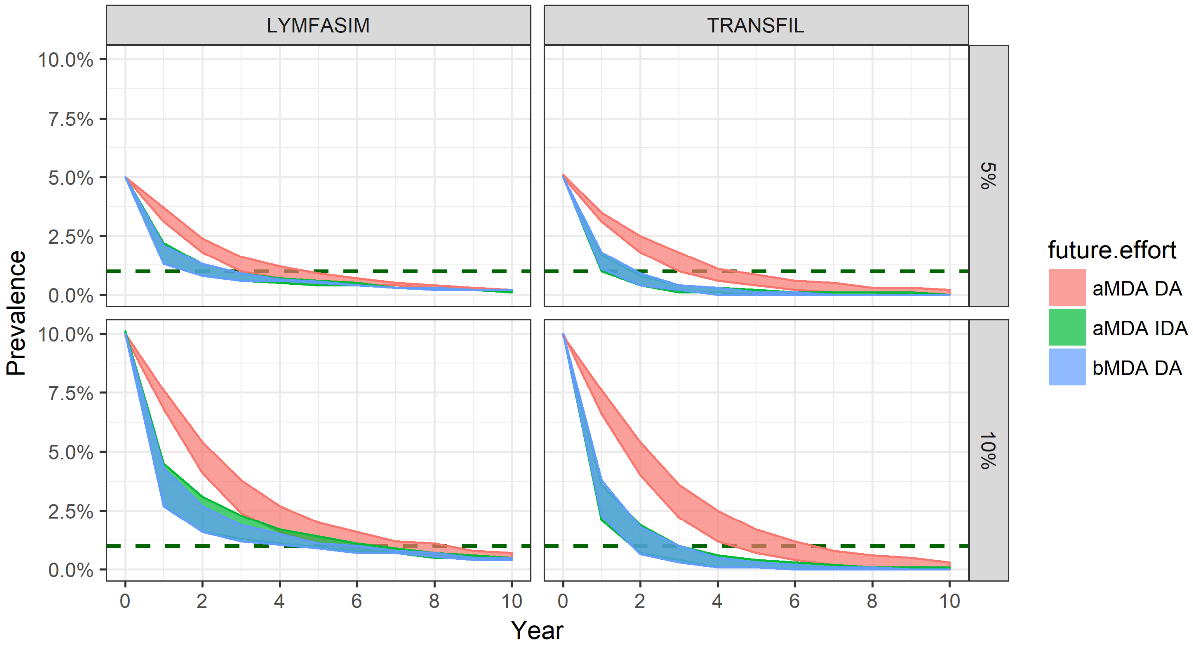

Figure 11.1. Model predicted trends in mf prevalence over time. The median mf prevalence over time is shown over the 501 runs closest to the mf prevalence given on the right. Coloured bands span the 65 to 80% intervention coverage range.

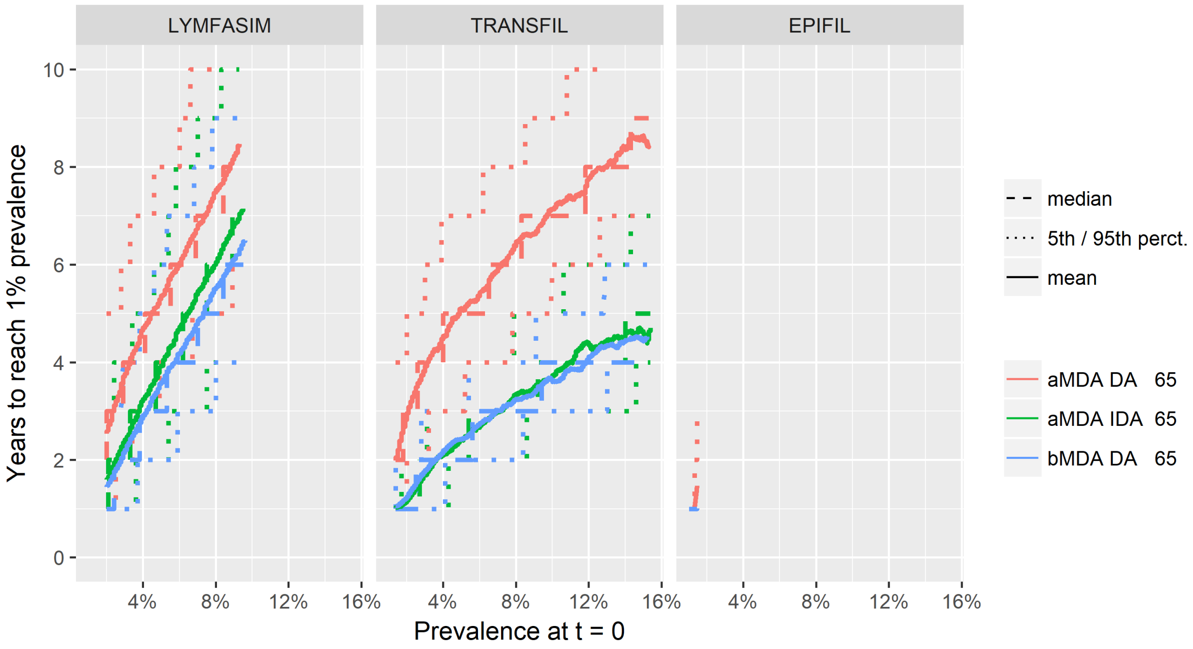

Figure 11.2. Predicted time to reach 1% mf prevalence. Shown are the moving median, the moving 5th and 95th percentile, and the moving average number of intervention years needed to bring mf prevalence below

1% (moving window size = 501. To enable calculation of the average any runs that did not achieve the 1% prevalence threshold by t=20 were set to achieving it by t=21).

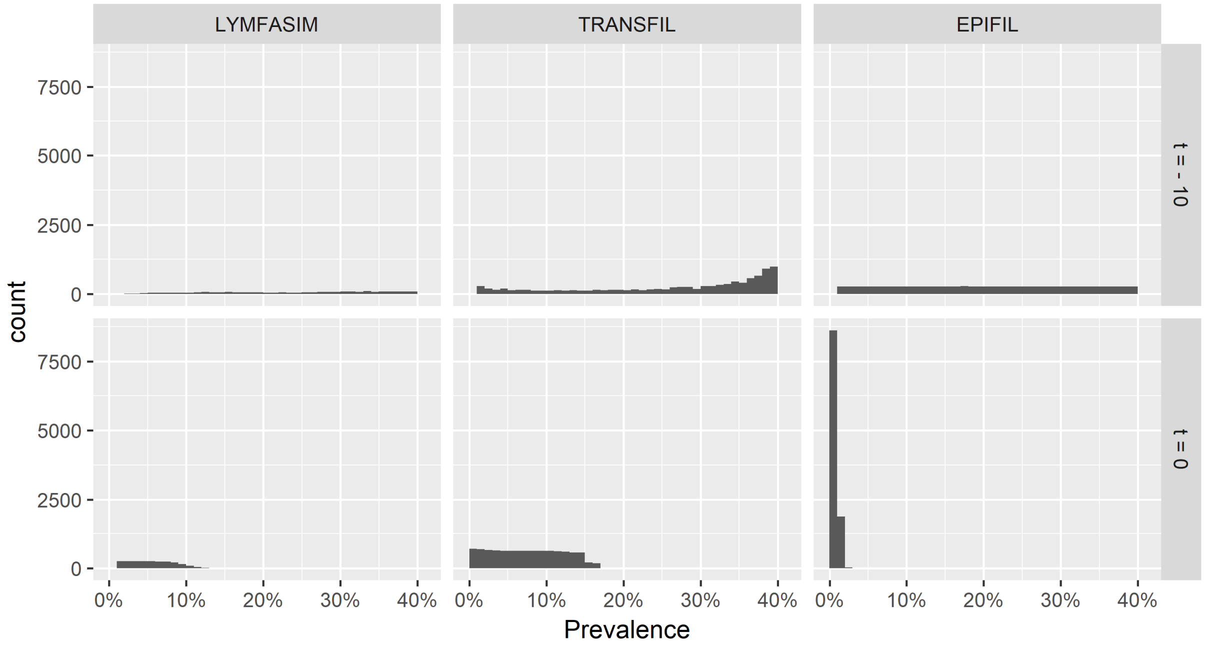

Figure 11.3. Distribution of the mf prevalence. Prevalence shown prior to any control (depending on the past intervention setting this is at *t* = -10, *t* = -2 or *t* = 0), and (if different) mf prevalence at *t* = 0. Binwidth = 1%.

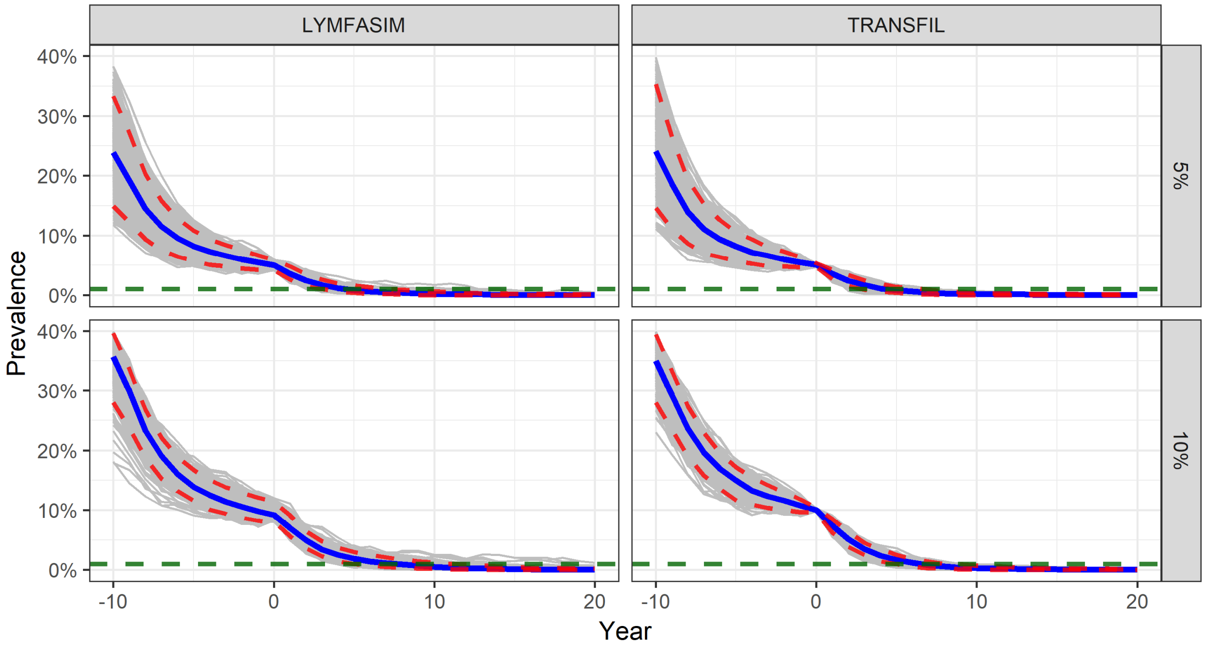

Figure 11.4. Model predicted trends in mf prevalence over time for the reference scenario. Future effort is as currently recommended: aMDA DA 65. In thin grey lines the 501 runs closest to the prevalence shown on the right. Thick blue line: median of these runs. Red dashed lines: 5th and 95th percentile of the runs respectively. Lower green line: the 1% prevalence level goal.

12. AFRICA, DEC+ALB (ONCHOCERCIASIS AND LOA LOA FREE) FAILURE SCENARIO 2

Region: Africa. Parasite species: *Wuchereria bancrofti*. Vector: *Anopheles species*.
History of control: Programme failure after 10 years of MDA with 30% coverage.

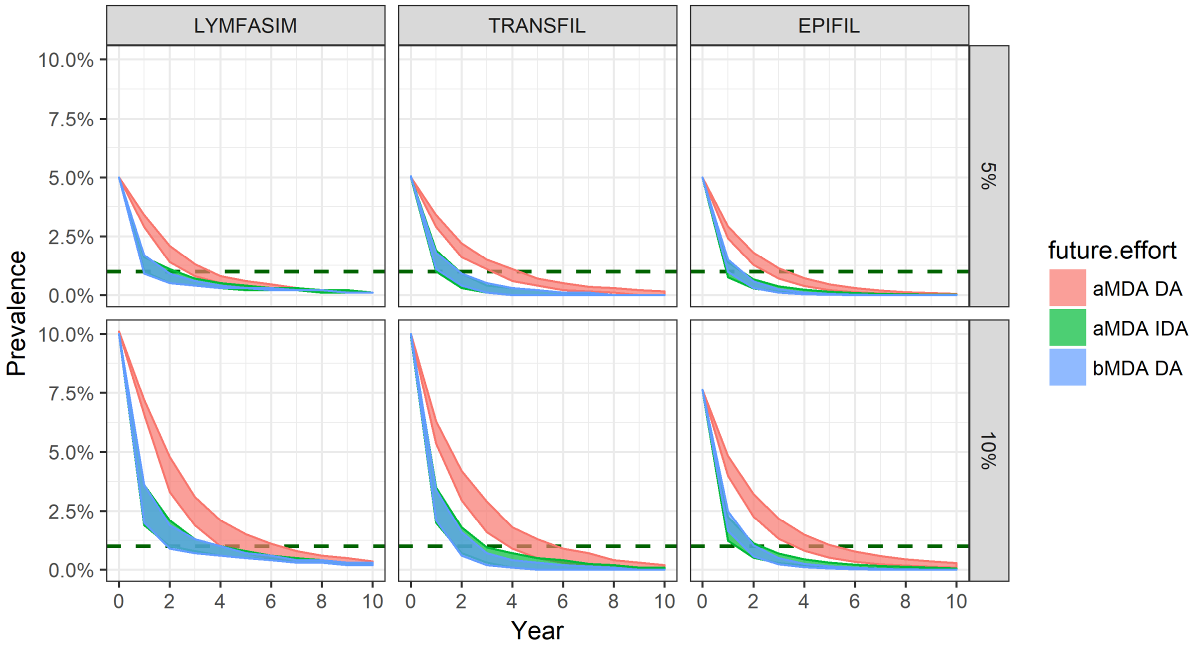

Figure 12.1. Model predicted trends in mf prevalence over time. The median mf prevalence over time is shown over the 501 runs closest to the mf prevalence given on the right. Coloured bands span the 65 to 80% intervention coverage range.

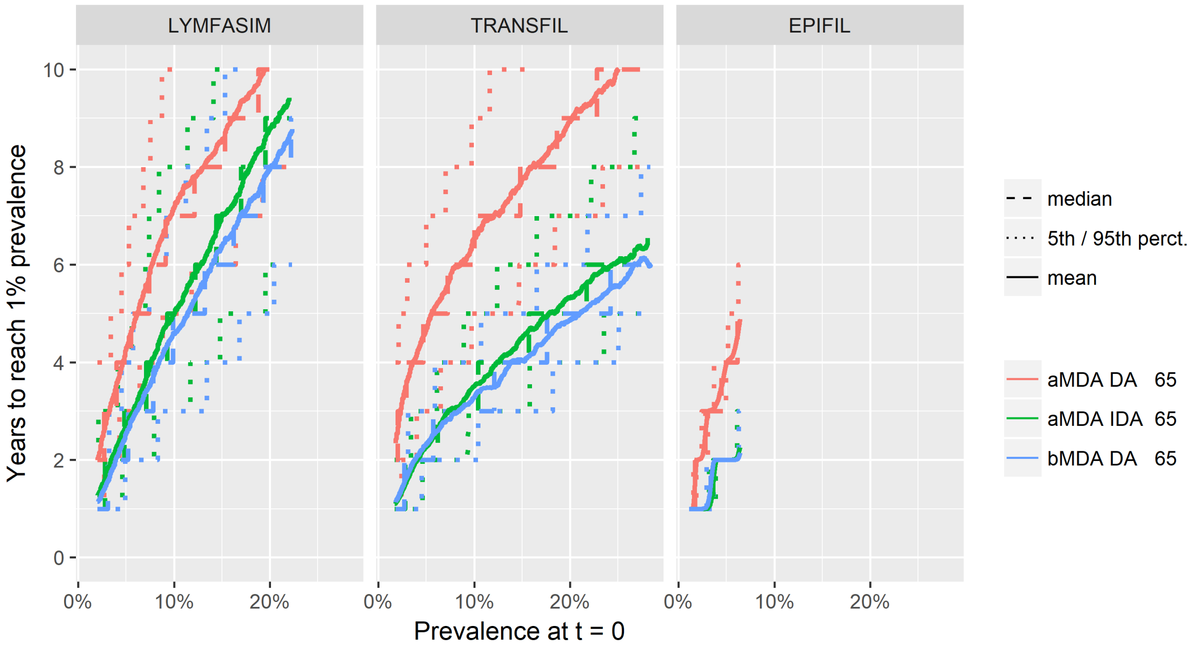

Figure 12.2. Predicted time to reach 1% mf prevalence. Shown are the moving median, the moving 5th and 95th percentile, and the moving average number of intervention years needed to bring mf prevalence below

1% (moving window size = 501. To enable calculation of the average any runs that did not achieve the 1% prevalence threshold by t=20 were set to achieving it by t=21).

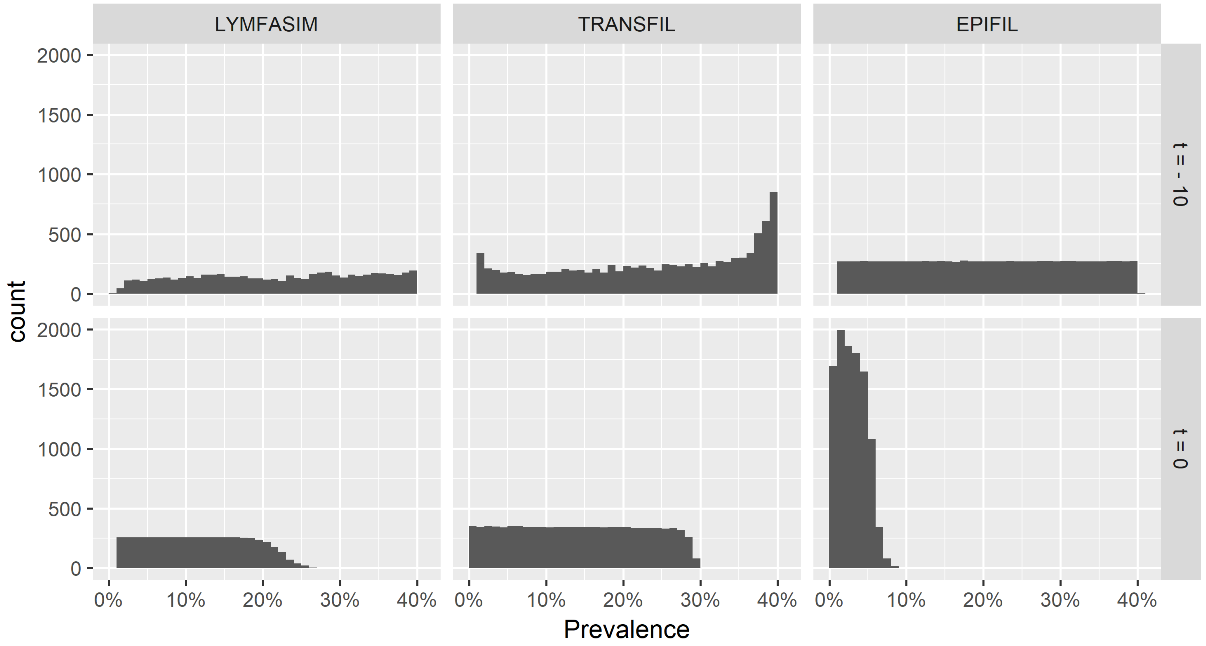

Figure 12.3. Distribution of the mf prevalence. Prevalence shown prior to any control (depending on the past intervention setting this is at *t* = -10, *t* = -2 or *t* = 0), and (if different) mf prevalence at *t* = 0. Binwidth = 1%.

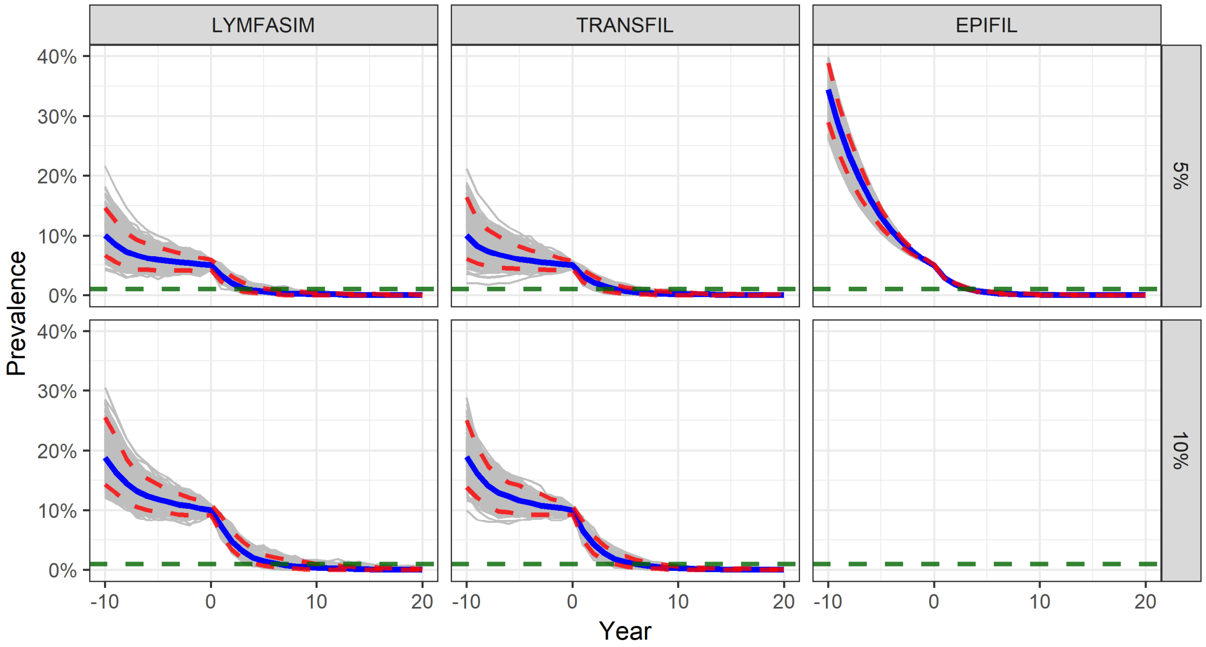

Figure 12.4. Model predicted trends in mf prevalence over time for the reference scenario. Future effort is as currently recommended: aMDA DA 65. In thin grey lines the 501 runs closest to the prevalence shown on the right. Thick blue line: median of these runs. Red dashed lines: 5th and 95th percentile of the runs respectively. Lower green line: the 1% prevalence level goal.

13. AFRICA, IVM+ALB (ONCHOCERCIASIS COENDEMIC LOA LOA FREE), TREATMENT NAIVE

Region: Africa. Parasite species: *Wuchereria bancrofti*. Vector: *Anopheles species*. History of control: no prior MDA.

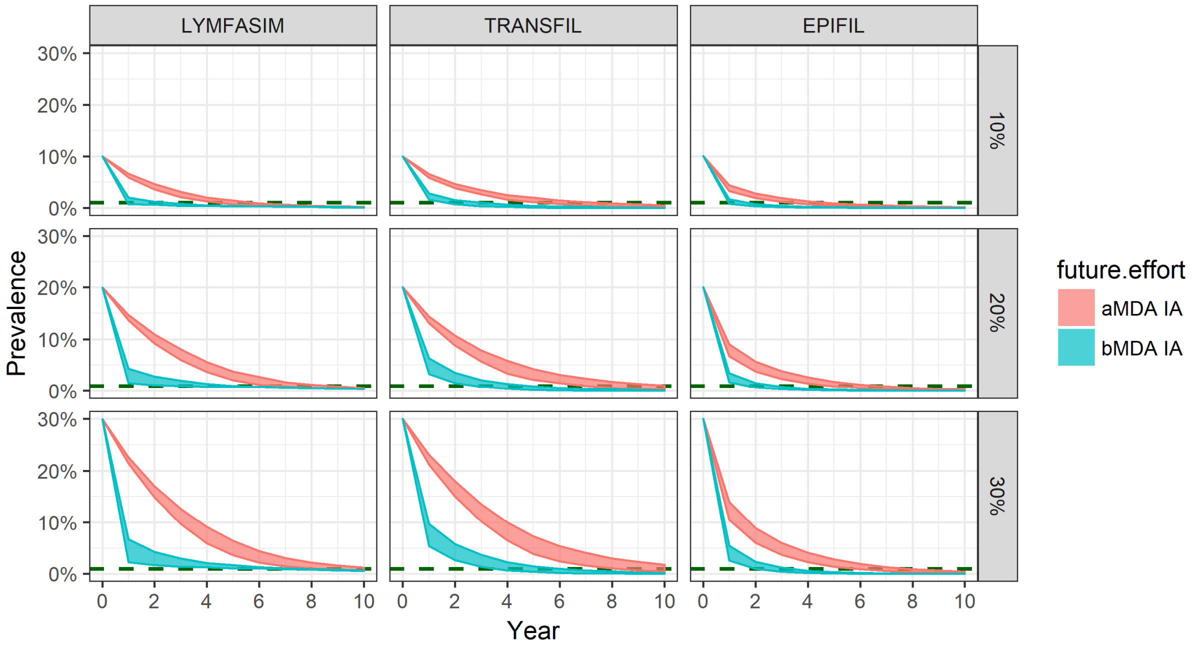

Figure 13.1. Model predicted trends in mf prevalence over time. The median mf prevalence over time is shown over the 501 runs closest to the mf prevalence given on the right. Coloured bands span the 65 to 80% intervention coverage range.

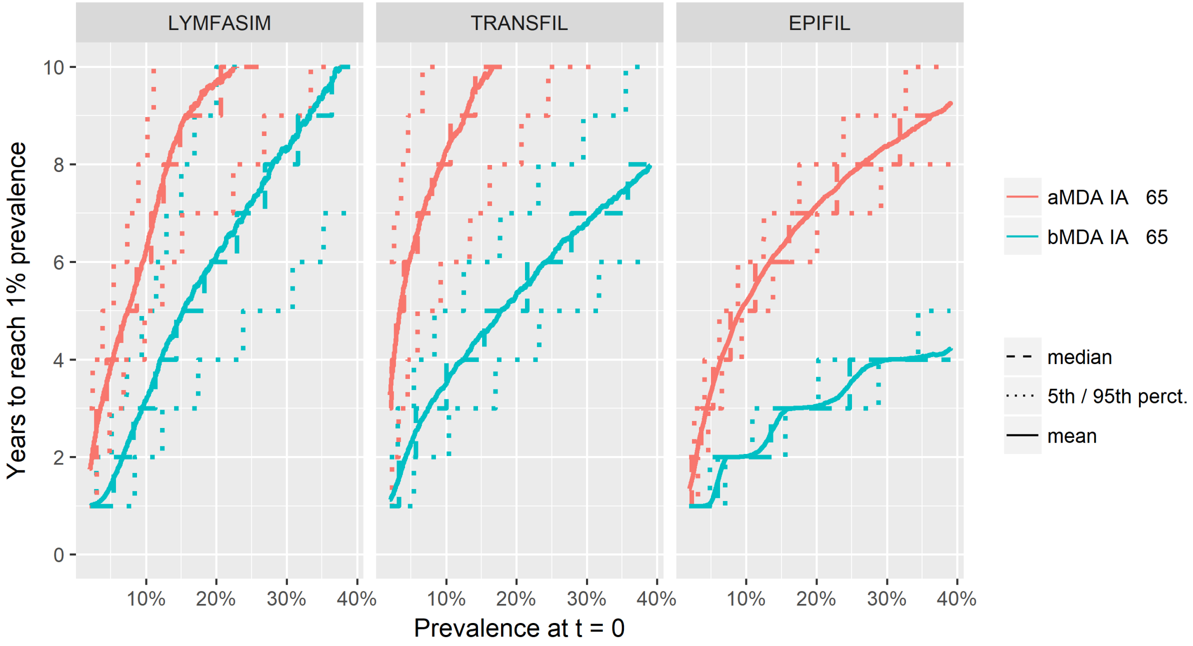

Figure 13.2. Predicted time to reach 1% mf prevalence. Shown are the moving median, the moving 5th and 95th percentile, and the moving average number of intervention years needed to bring mf prevalence below

1% (moving window size = 501. To enable calculation of the average any runs that did not achieve the 1% prevalence threshold by t=20 were set to achieving it by t=21).

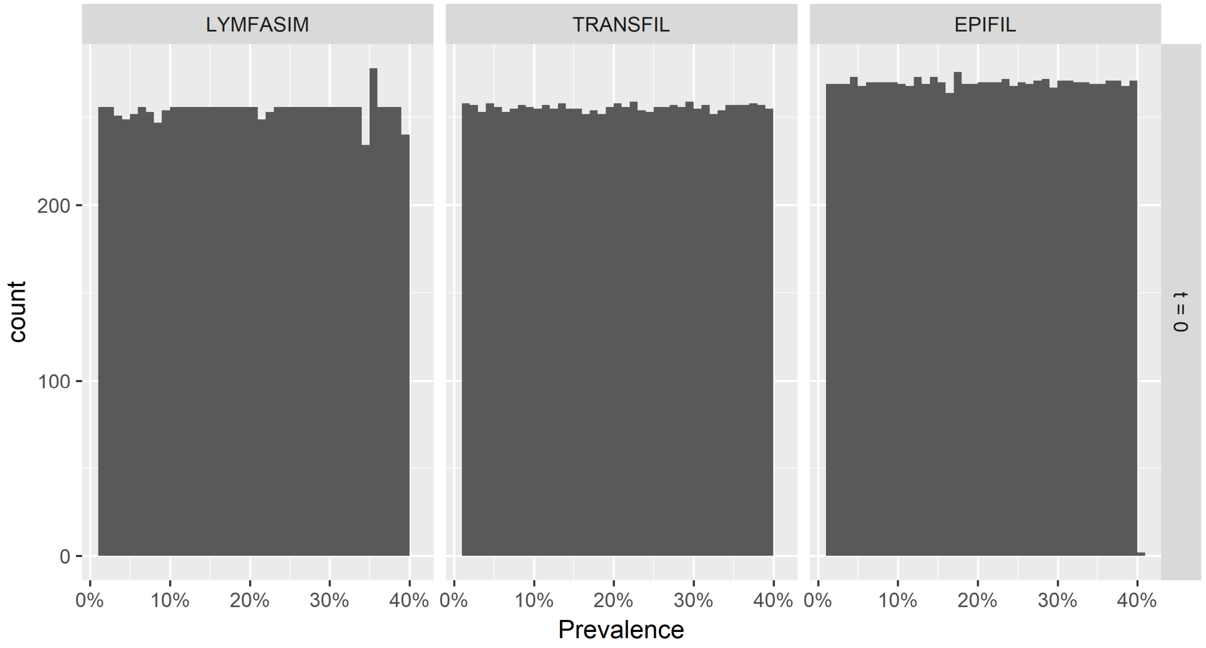

Figure 13.3. Distribution of the mf prevalence. Prevalence shown prior to any control (depending on the past intervention setting this is at *t* = -10, *t* = -2 or *t* = 0), and (if different) mf prevalence at *t* = 0. Binwidth = 1%.

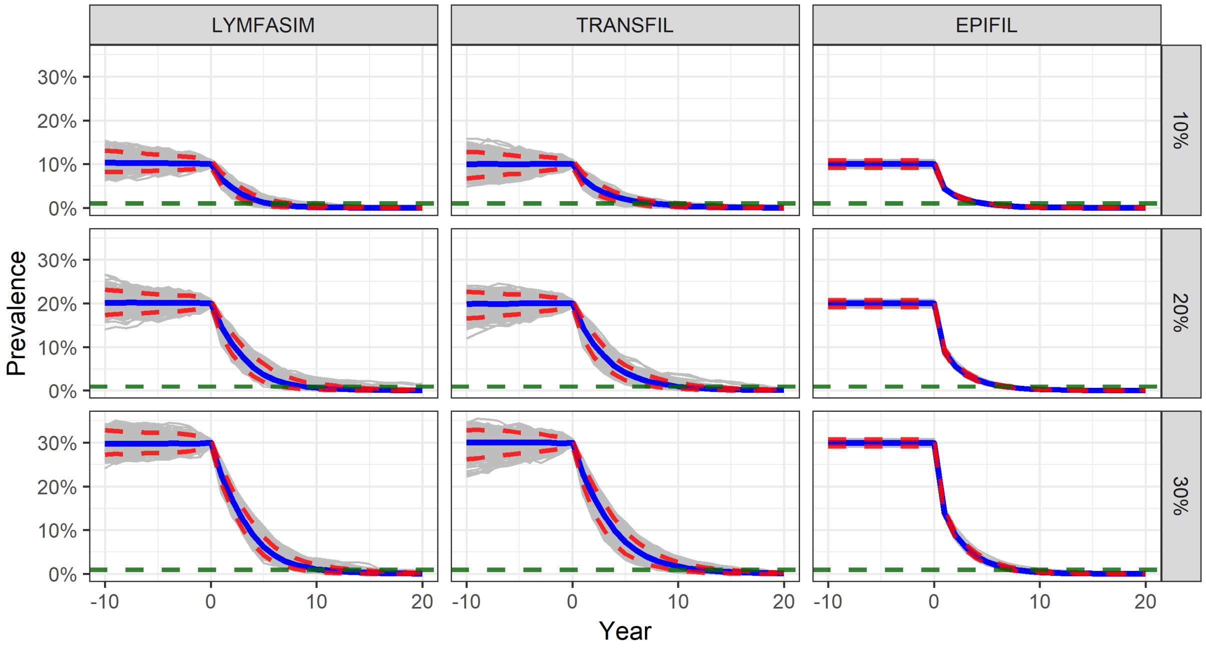

Figure 13.4. Model predicted trends in mf prevalence over time for the reference scenario. Future effort is as currently recommended: aMDA IA 65. In thin grey lines the 501 runs closest to the prevalence shown on the right. Thick blue line: median of these runs. Red dashed lines: 5th and 95th percentile of the runs respectively. Lower green line: the 1% prevalence level goal.

14. AFRICA, IVM+ALB (ONCHOCERCIASIS COENDEMIC LOA LOA FREE), RECENT START MDA

Region: Africa. Parasite species: *Wuchereria bancrofti*. Vector: *Anopheles species*. History of control: 2 years of MDA with DEC+ALB at 65% coverage.

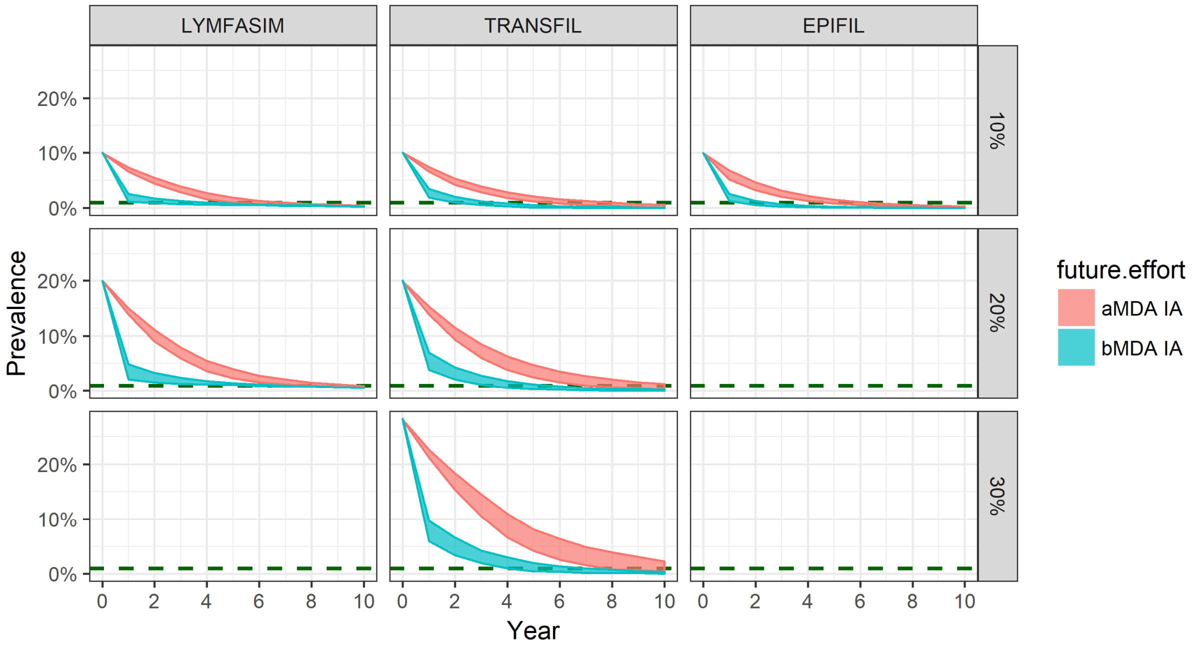

Figure 14.1. Model predicted trends in mf prevalence over time. The median mf prevalence over time is shown over the 501 runs closest to the mf prevalence given on the right. Coloured bands span the 65 to 80% intervention coverage range.

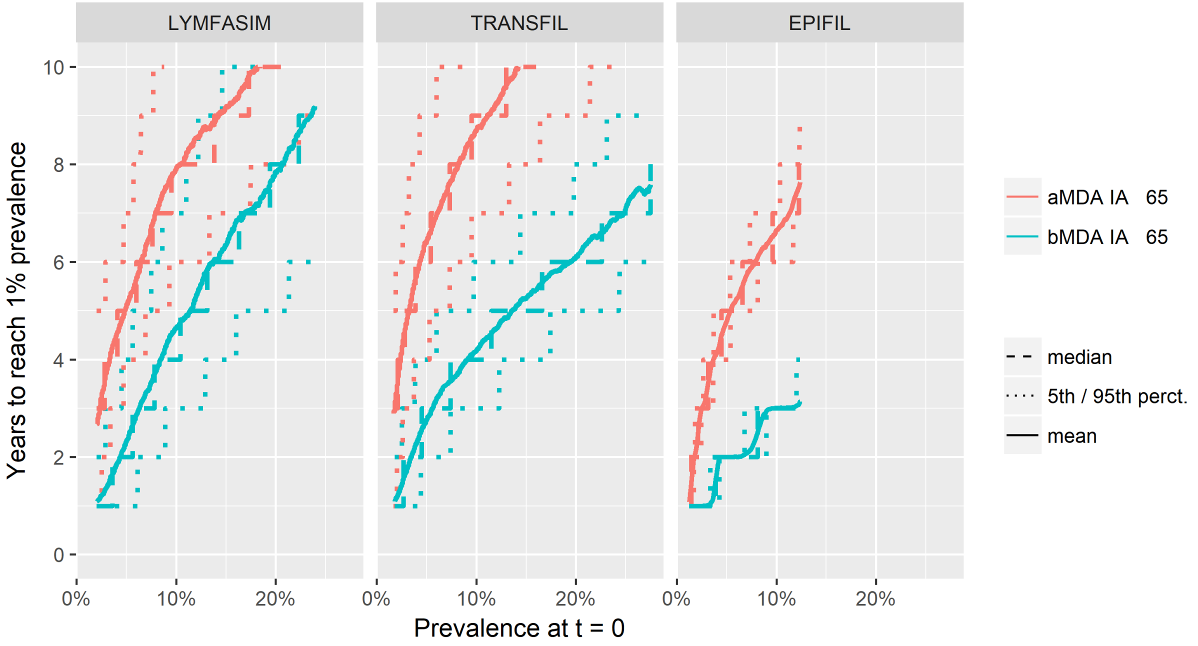

Figure 14.2. Predicted time to reach 1% mf prevalence. Shown are the moving median, the moving 5th and 95th percentile, and the moving average number of intervention years needed to bring mf prevalence below

1% (moving window size = 501. To enable calculation of the average any runs that did not achieve the 1% prevalence threshold by t=20 were set to achieving it by t=21).

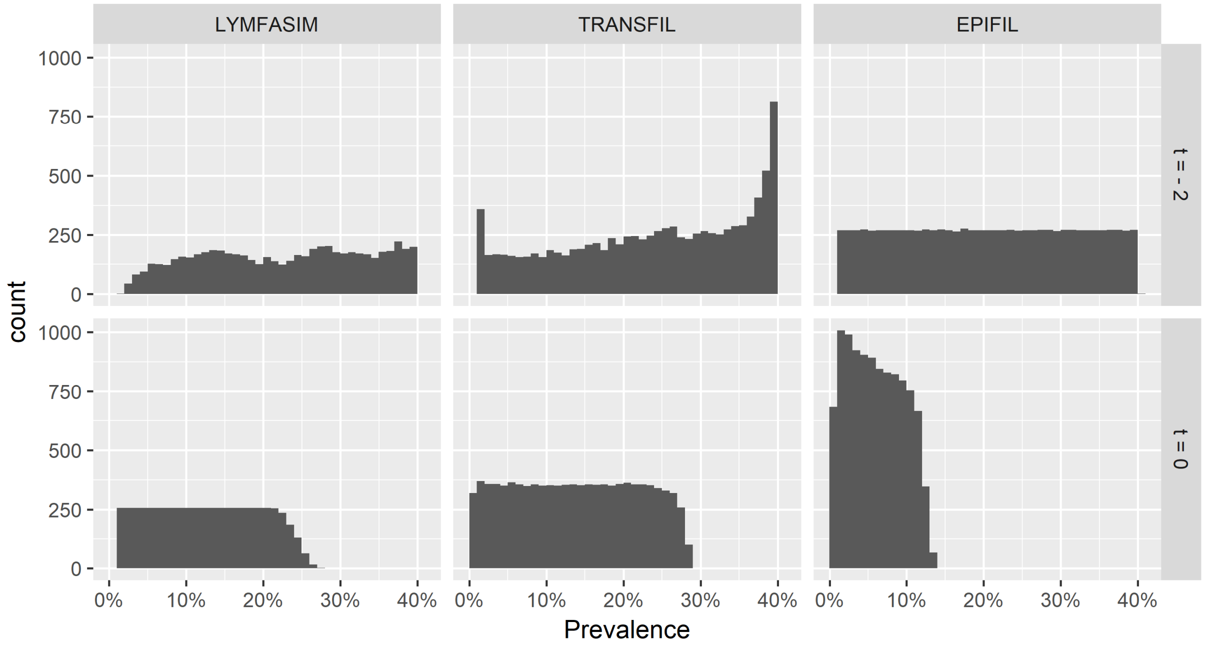

Figure 14.3. Distribution of the mf prevalence. Prevalence shown prior to any control (depending on the past intervention setting this is at t = -10, t = -2 or t = 0), and (if different) mf prevalence at t = 0. Binwidth = 1%.

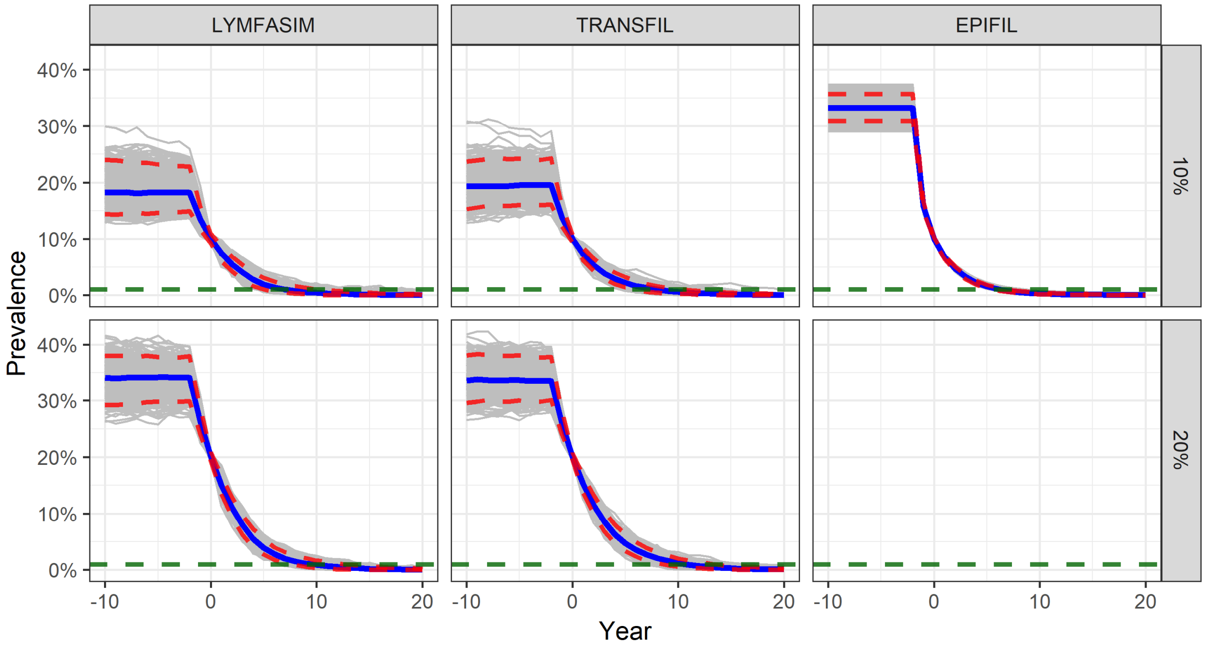

Figure 14.4. Model predicted trends in mf prevalence over time for the reference scenario. Future effort is as currently recommended: aMDA IA 65. In thin grey lines the 501 runs closest to the prevalence shown on the right. Thick blue line: median of these runs. Red dashed lines: 5th and 95th percentile of the runs respectively. Lower green line: the 1% prevalence level goal.

15. AFRICA, IVM+ALB (ONCHOCERCIASIS COENDEMIC LOA LOA FREE), FAILURE SCENARIO 1

Region: Africa. Parasite species: *Wuchereria bancrofti*. Vector: *Anopheles species*.
History of control: Programme failure after 10 years of MDA with 50% coverage.

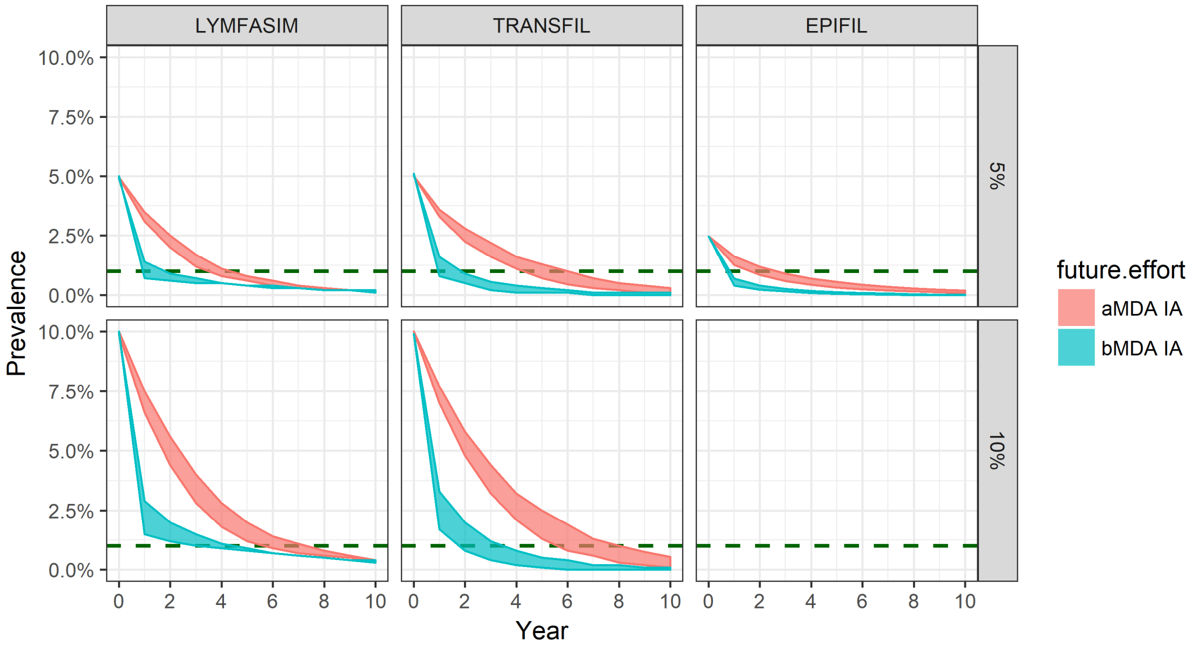

Figure 15.1. Model predicted trends in mf prevalence over time. The median mf prevalence over time is shown over the 501 runs closest to the mf prevalence given on the right. Coloured bands span the 65 to 80% intervention coverage range.

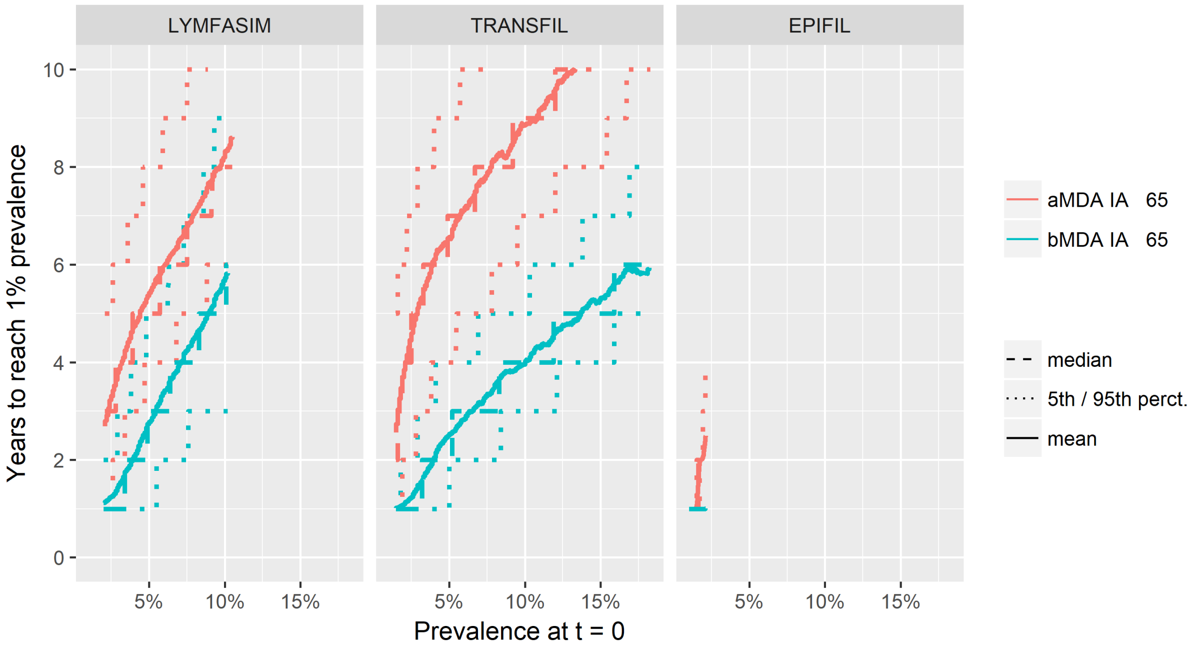

Figure 15.2. Predicted time to reach 1% mf prevalence. Shown are the moving median, the moving 5th and 95th percentile, and the moving average number of intervention years needed to bring mf prevalence below

1% (moving window size = 501. To enable calculation of the average any runs that did not achieve the 1% prevalence threshold by t=20 were set to achieving it by t=21).

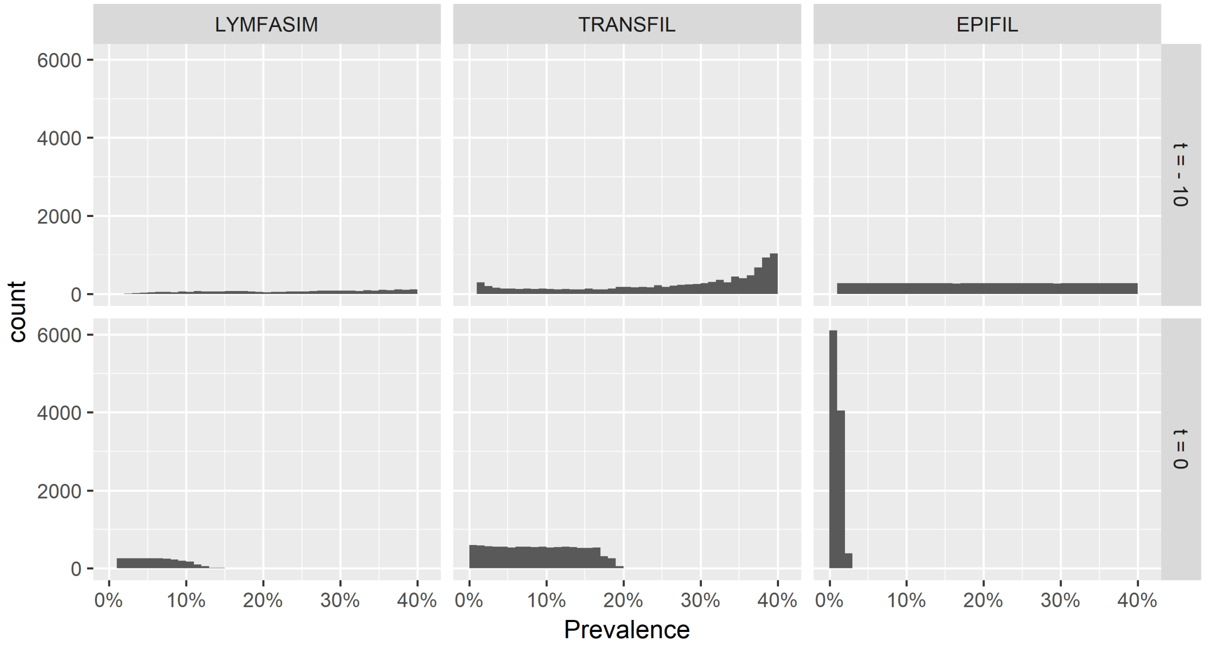

Figure 15.3. Distribution of the mf prevalence. Prevalence shown prior to any control (depending on the past intervention setting this is at t = -10, t = -2 or t = 0), and (if different) mf prevalence at t = 0. Binwidth = 1%.

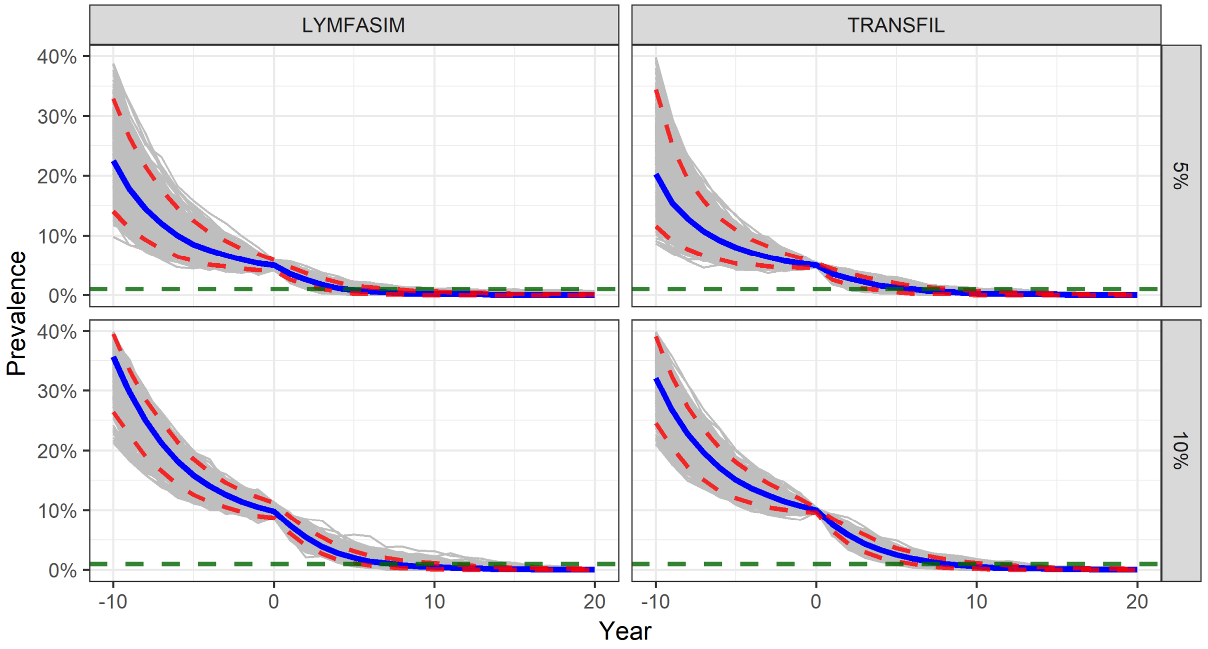

Figure 15.4. Model predicted trends in mf prevalence over time for the reference scenario. Future effort is as currently recommended: aMDA IA 65. In thin grey lines the 501 runs closest to the prevalence shown on the right. Thick blue line: median of these runs. Red dashed lines: 5th and 95th percentile of the runs respectively. Lower green line: the 1% prevalence level goal.

16. AFRICA, IVM+ALB (ONCHOCERCIASIS COENDEMIC LOA LOA FREE), FAILURE SCENARIO 2

Region: Africa. Parasite species: *Wuchereria bancrofti*. Vector: *Anopheles species*. History of control: Programme failure after 10 years of MDA with 30% coverage.

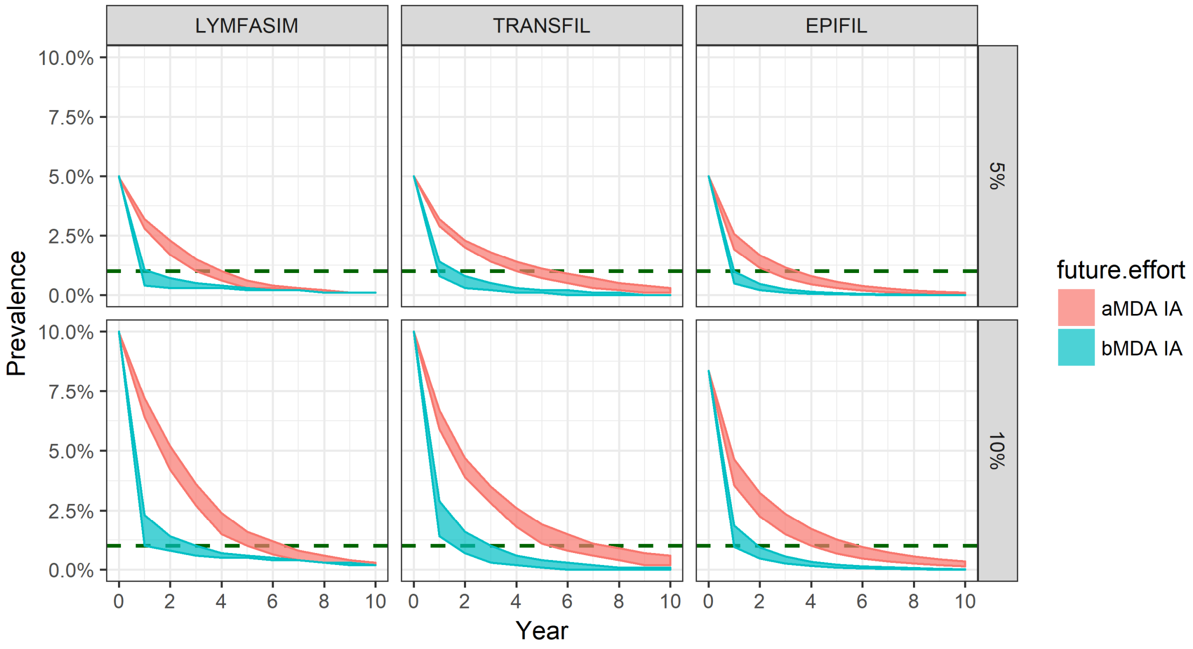

Figure 16.1. Model predicted trends in mf prevalence over time. The median mf prevalence over time is shown over the 501 runs closest to the mf prevalence given on the right. Coloured bands span the 65 to 80% intervention coverage range.

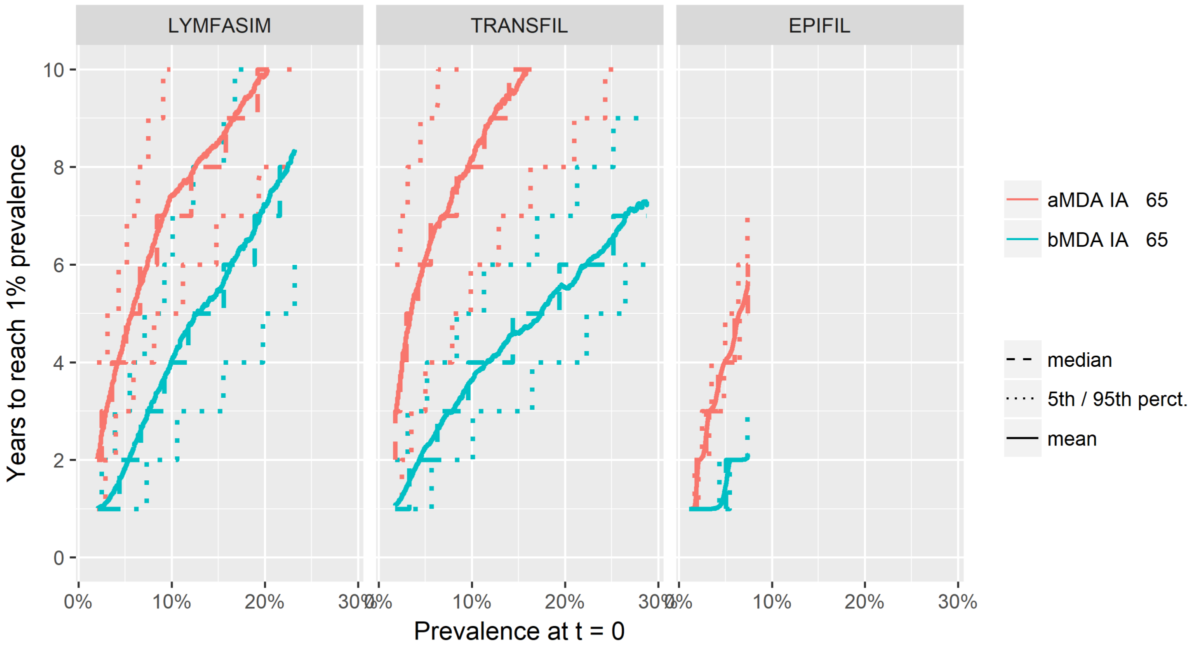

Figure 16.2. Predicted time to reach 1% mf prevalence. Shown are the moving median, the moving 5th and 95th percentile, and the moving average number of intervention years needed to bring mf prevalence below

1% (moving window size = 501. To enable calculation of the average any runs that did not achieve the 1% prevalence threshold by t=20 were set to achieving it by t=21).

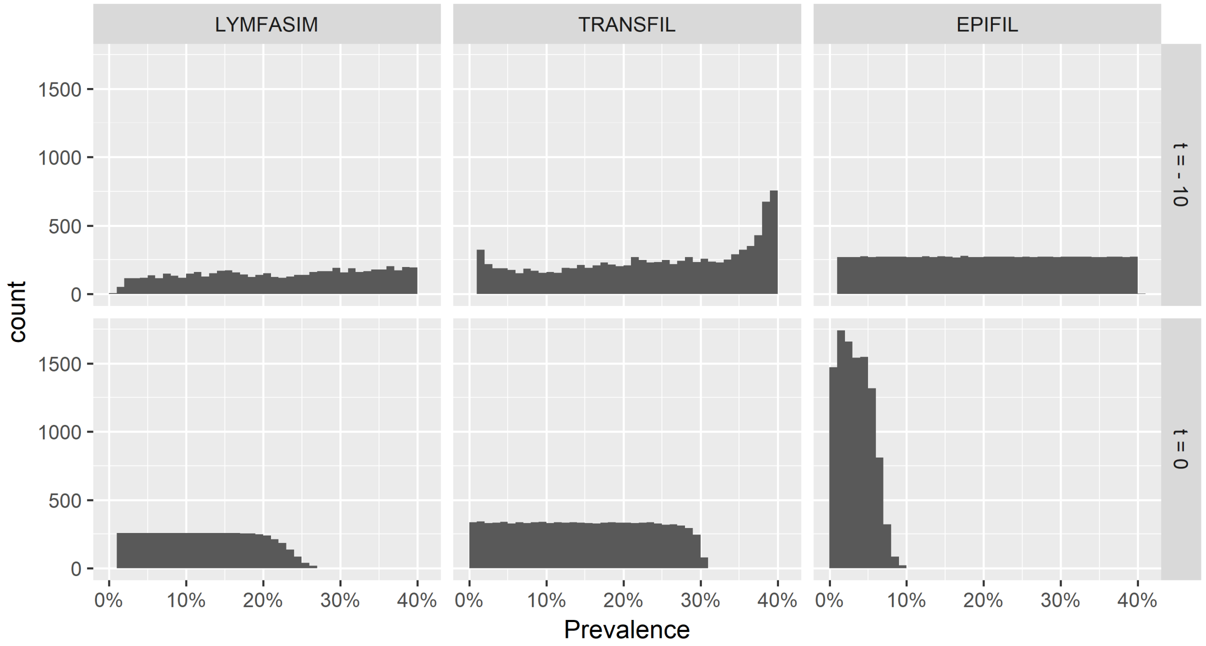

Figure 16.3. Distribution of the mf prevalence. Prevalence shown prior to any control (depending on the past intervention setting this is at *t* = -10, *t* = -2 or *t* = 0), and (if different) mf prevalence at *t* = 0. Binwidth = 1%.

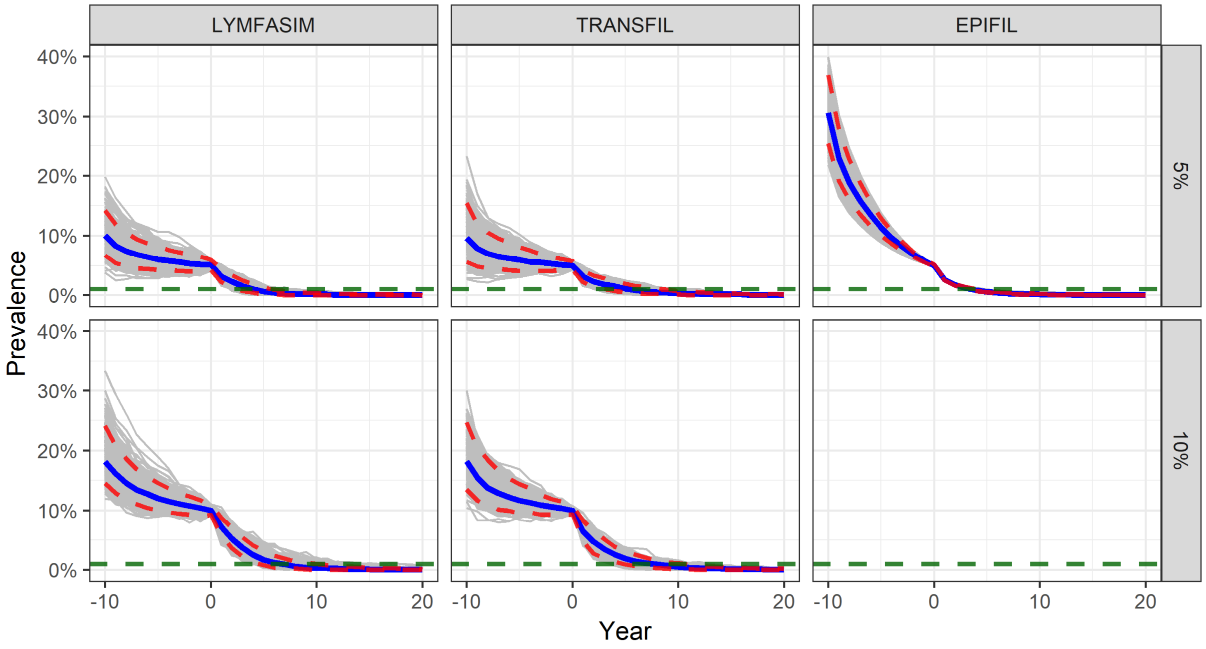

Figure 16.4. Model predicted trends in mf prevalence over time for the reference scenario. Future effort is as currently recommended: aMDA IA 65. In thin grey lines the 501 runs closest to the prevalence shown on the right. Thick blue line: median of these runs. Red dashed lines: 5th and 95th percentile of the runs respectively. Lower green line: the 1% prevalence level goal.

17. AFRICA, ALB ALONE (LOA LOA COENDEMIC), TREATMENT NAIVE

Region: Africa. Parasite species: *Wuchereria bancrofti*. Vector: *Anopheles species*.
History of control: no prior MDA.

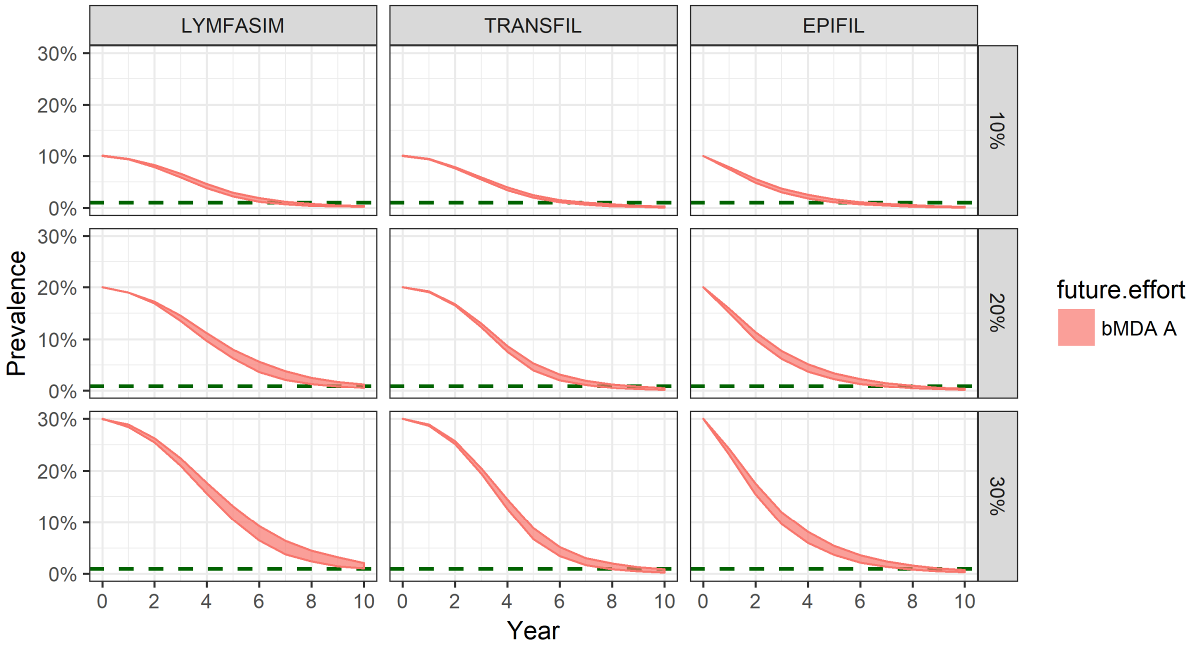

Figure 17.1. Model predicted trends in mf prevalence over time. The median mf prevalence over time is shown over the 501 runs closest to the mf prevalence given on the right. Coloured bands span the 65 to 80% intervention coverage range.

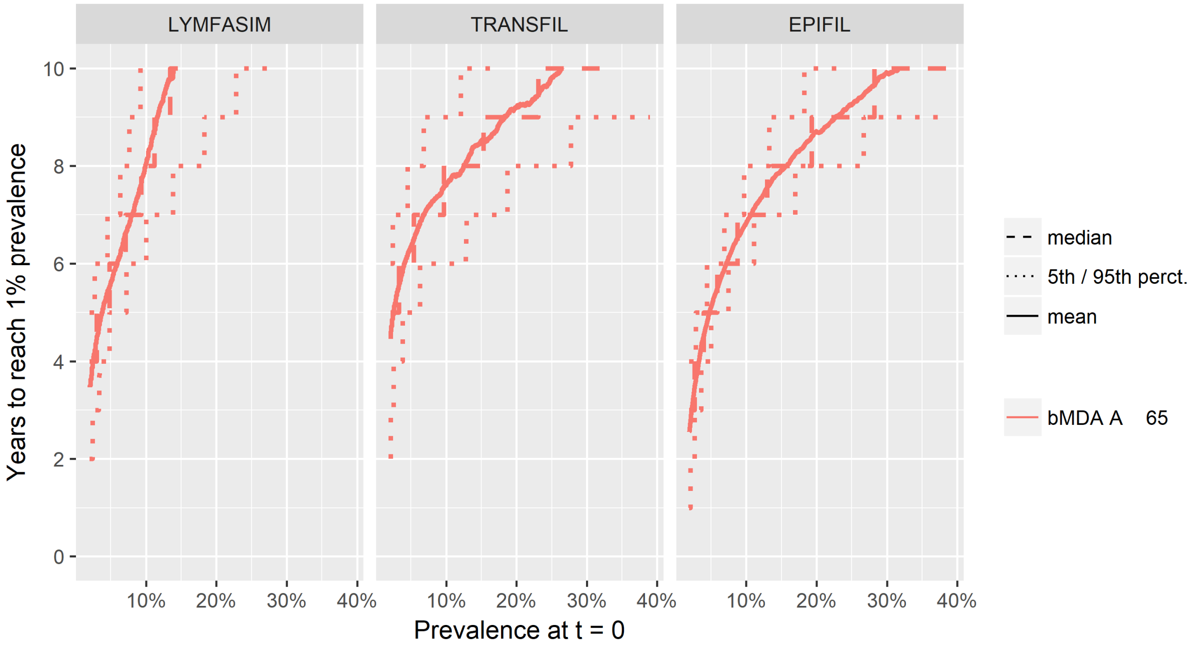

Figure 17.2. Predicted time to reach 1% mf prevalence. Shown are the moving median, the moving 5th and 95th percentile, and the moving average number of intervention years needed to bring mf prevalence below

1% (moving window size = 501. To enable calculation of the average any runs that did not achieve the 1% prevalence threshold by t=20 were set to achieving it by t=21).

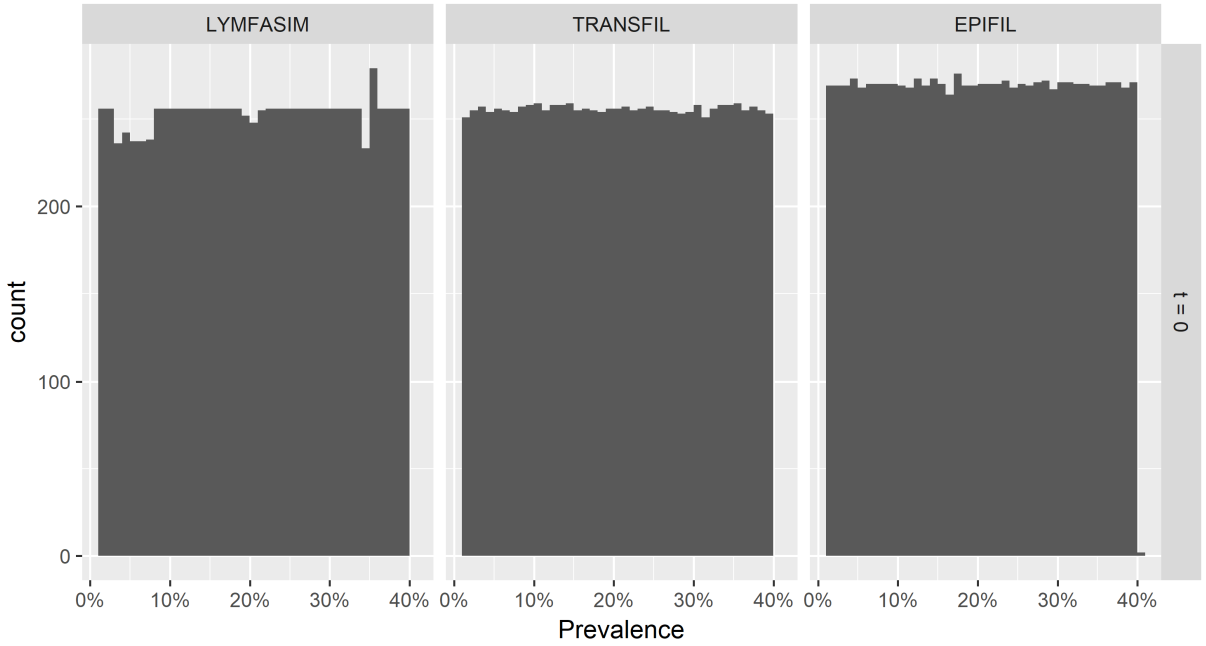

Figure 17.3. Distribution of the mf prevalence. Prevalence shown prior to any control (depending on the past intervention setting this is at t = -10, t = -2 or t = 0), and (if different) mf prevalence at t = 0. Binwidth = 1%.

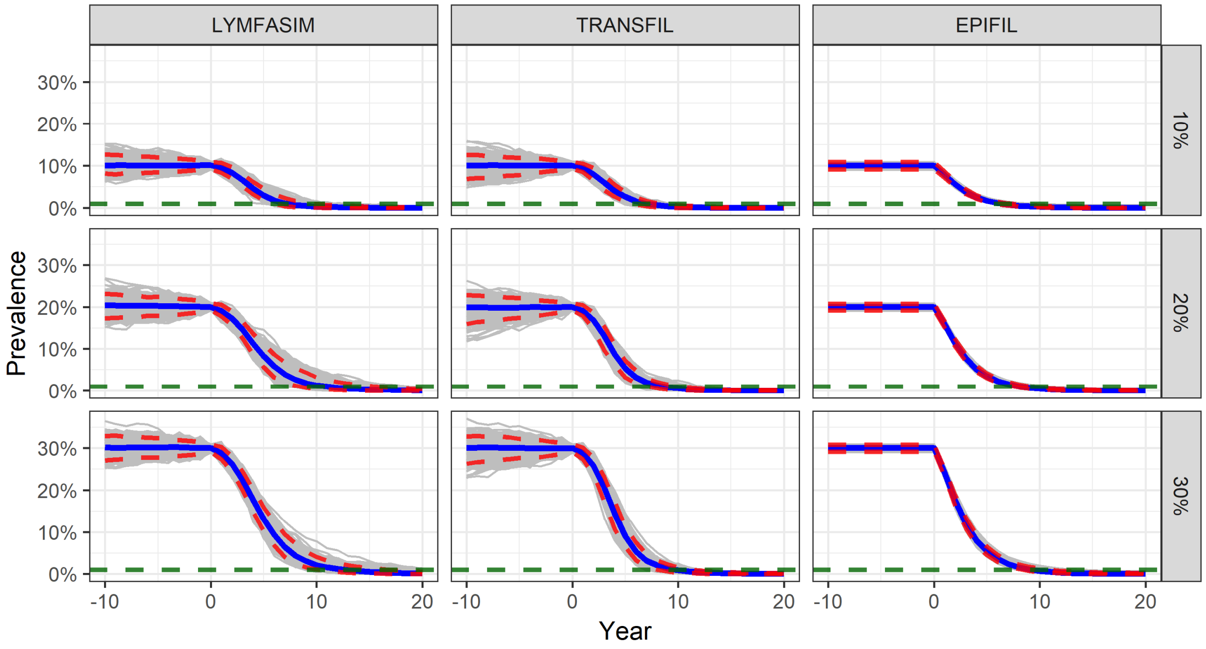

Figure 17.4. Model predicted trends in mf prevalence over time for the reference scenario. Future effort is as currently recommended: bMDA A 65. In thin grey lines the 501 runs closest to the prevalence shown on the right. Thick blue line: median of these runs. Red dashed lines: 5th and 95th percentile of the runs respectively. Lower green line: the 1% prevalence level goal.
